# Computer-Aided Discovery of Natural Compounds Targeting the ADAR2 dsRBD2-RNA Interface and Computational Modeling of Full-Length ADAR2 Protein Structure

**DOI:** 10.3390/ijms26094075

**Published:** 2025-04-25

**Authors:** Carolyn N. Ashley, Emmanuel Broni, Michelle Pena-Martinez, Chanyah M. Wood, Samuel K. Kwofie, Whelton A. Miller

**Affiliations:** 1 Department of Medicine, Loyola University Medical Center, Loyola University Chicago, Maywood, IL 60153, USA; cashley1@luc.edu (C.N.A.); ebroni@luc.edu (E.B.);; 2 Department of Molecular Pharmacology & Neuroscience, Loyola University Medical Center, Loyola University Chicago, Maywood, IL 60153, USA; 3 Department of Biomedical Engineering, School of Engineering Sciences, College of Basic & Applied Sciences, University of Ghana, Legon, Accra LG 77, Ghana; skkwofie@ug.edu.gh

**Keywords:** ADAR, full-length ADAR2, molecular dynamics simulations, homology modeling, MM/PBSA calculations, mesothelioma, computer-aided drug discovery, natural compounds, RNA editing

## Abstract

Mesothelioma is a rare and aggressive cancer linked to asbestos exposure and characterized by rapid metastasis and poor prognosis. Inhibition of adenosine deaminase acting on dsRNA 2 (ADAR2) RNA binding but not ADAR2 editing has shown antitumor effects in mesothelioma. Natural compounds from the Traditional Chinese Medicine (TCM) database were docked to the RNA-binding interface of ADAR2’s second dsRNA binding domain (dsRBD2), and their drug-likeness and predicted safety were assessed. Eight ligands (ZINC000085597263, ZINC000085633079, ZINC000014649947, ZINC000034512861, ZINC000070454124, ZINC000085594944, ZINC000085633008, and ZINC000095909822) showed high binding affinity to dsRBD2 from molecular mechanics Poisson–Boltzmann surface area (MM/PBSA) calculations. Protein–ligand interactions were analyzed to identify key residues contributing to these binding affinities. Molecular dynamics (MD) simulations of dsRBD–ligand–RNA complexes revealed that four compounds (ZINC000085597263, ZINC000085633079, ZINC000014649947, and ZINC000034512861) had negative binding affinities to dsRBD2 in the presence of the RNA substrate GluR-2. Key residues, including Val164, Met165, Lys209, and Lys212, were crucial for ligand binding, even with RNA present, suggesting these compounds could inhibit dsRBD2’s RNA-binding function. The predicted biological activities of these compounds indicate potential anticancer properties, particularly for the treatment of mesothelioma. These compounds are structurally similar to known anti-mesothelioma agents or anticancer drugs, highlighting their therapeutic potential. Current mesothelioma treatments are limited. Optimization of these compounds, alone or in combination with current therapeutics, has potential for mesothelioma treatment. Additionally, five high-quality full-length ADAR2 models were developed. These models provide insights into ADAR2 function, mutation impacts, and potential areas for protein engineering to enhance stability, RNA-binding specificity, or protein interactions, particularly concerning dimerization or complex formation with other proteins and RNAs.

## 1. Introduction

RNA editing, specifically adenosine to inosine (A-I) conversion, regulates a wide variety of molecular mechanisms implicated in cancer development and progression [1]. Primarily, A-I editing is catalyzed by adenosine deaminases acting on RNA (ADAR), a family of highly conserved modular RNA editing proteins [2]⁠. The ADAR family has three primary members, ADAR1 and ADAR2 that are catalytically active, and the third member ADAR3 that is catalytically inactive but does have RNA editing-independent functions [3]. ADAR1 and ADAR2 are present across nearly all human tissues to varying degrees, whereas ADAR3 is uniquely expressed in the brain [4]. Both ADAR1 and ADAR2 have been implicated in the pathophysiology of multiple cancers [5]⁠. ADARs have normal regulatory functions including their involvement in editing targets that contribute to regulating cellular proliferation, cell motility, cell survival, and apoptosis [6]. ADARs are involved in a variety of molecular pathways that can influence the protein diversity or gene expression levels of their targets. For some examples, A-I editing can affect pre-mRNAs resulting in changes in amino acid coding and altered protein function, as well as influence the target recognition, maturation, and stability of microRNAs (miRNA) and small interfering RNAs (siRNAs) leading to changes in specificity and targeting important for RNA interference regulatory functions [7,8,9]⁠. ADAR editing of miRNAs is one mechanism that links altered A-I editing to changes in gene expression and cancer cell growth [10]. Due to ADAR involvement in protein diversity and gene expression regulation, when ADAR editing or ADAR expression is dysregulated, this can lead to cancer promotion or the downregulation of tumor suppressor pathways, making ADAR1 and ADAR2 appealing drug targets for oncology. ADAR proteins are involved in a multitude of broader mechanisms that influence cancer. Notably, for both ADAR1 and ADAR2, there is a complicated interworking in the promotion and suppression of cancer-related proteins. Cancer therapeutics under development harness context-specific mechanisms to target tissues, cell/cancer type, and ADAR variant.

ADAR2 is in most contexts a tumor-suppressor protein, i.e., cancer progression is in many contexts associated with a loss of ADAR2 editing [10]⁠. In contrast, ADAR1 has dominantly been reported as an oncogenic protein, where overediting of targets by ADAR1 is associated with oncogenesis, tumor metastasis, and therapeutic resistance [11,12]⁠. ADAR2 editing in glioblastoma multiforme (GBM) is a clear example connecting altered ADAR2 editing with cancer progression. In GBM, loss of ADAR2 editing of specific miRNAs results in differential targeting and in turn leads to increased tumor cell growth and invasiveness [13,14]⁠. The recovery of specific ADAR2 editing sites has potential for therapeutic benefit in GBM. For example, ADAR2 editing of CDC14B is critical for Skp2 degradation to block cell cycle signaling [15]⁠. In GBM, loss of ADAR2 editing of CDC14B leads to an increase in Skp2 signaling and increased tumor growth, but this growth is inhibited with the addition of edited CDC14B transcripts [15]⁠. For most cancer types associated with ADAR2, under-editing therapeutic strategies harnessing site-directed approaches are of interest. One such strategy, Recruiting Endogenous ADAR to Specific Transcripts for Oligonucleotide-mediated RNA Editing (RESTORE), utilizes guide RNAs like antisense oligonucleotides (ASOs) to target endogenous ADARs to specific transcripts implicated in disease ultimately increasing the editing of particular target sites [16]⁠. Intriguingly, there are a few contexts where ADAR2 editing is associated with cancer promotion. ADAR2 involvement in mesothelioma is particularly of interest as ADAR2 involvement in mesothelioma represents a potentially novel mechanism.

Mesothelioma is a rare intractable cancer associated with asbestos exposure, aggressive metastasis, and poor prognosis [17]⁠. Asbestos has high toxicity and has been banned in many countries; however, in countries where asbestos is not banned, its use is ongoing in mass consumption [18]⁠. Disease related to asbestos exposure can take long terms to develop, sometimes spanning more than 40 years [19]⁠. In countries where asbestos has been banned, cases continue to occur and in countries where asbestos is still in use, associated incidences are on the rise [18]⁠. Studies focused on mesothelioma development reported that, in mice, chronic exposure to asbestos significantly increased ADAR editing in tumors [20]⁠. In mesothelioma, ADAR1 expression is homogenous, whereas ADAR2 expression is heterogeneous [21]. ADAR2 expression knockdown and recovery is supportive that ADAR2 influences mesothelioma cell growth [21]. Further, only siRNA knockdown of ADAR2 and not ADAR1 variants, p150 or p110, significantly reduced mesothelioma cell proliferation, cell invasion, and cell motility [22]. Overexpression experiments utilizing an ADAR2 mutant without RNA-binding capabilities, ADAR2-EAA, led to similar significant antitumor effects in TCC-MESO1 cells; however, overexpression of an ADAR2 mutant without RNA editing activity, ADAR2-T375A, did not [22]⁠. This suggests an RNA editing-independent mechanism that is reliant on RNA binding recognition by ADAR2’s second double-stranded RNA binding domain (dsRBD2).

**Figure 1:**
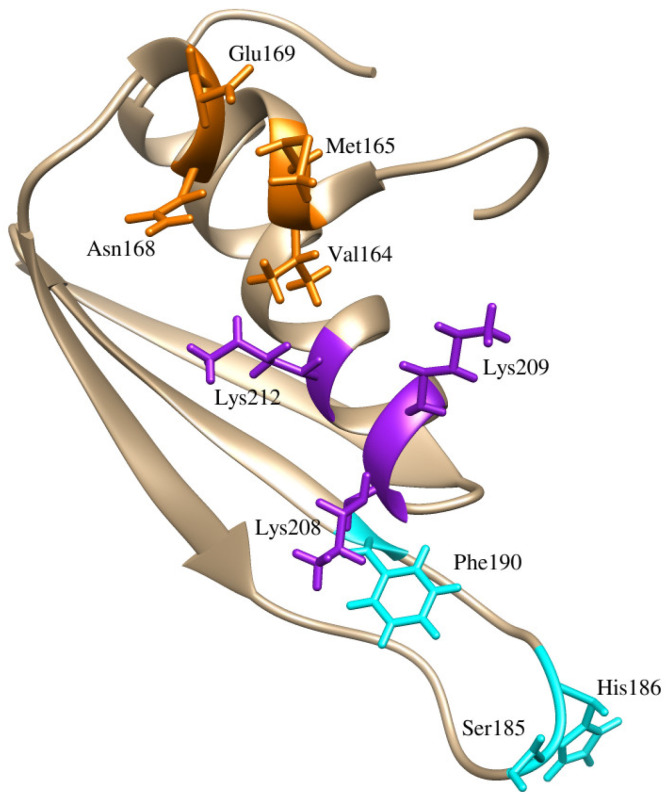
Residues involved in dsRBD2-RNA interface. Residues in orange are a part of the α1 helix, residues in cyan are in the β1-β2 loop, and residues in purple are the lysines within the KKNAK motif. Corresponding residues in PDB: 2L3J are Val237, Met238, Asn241, Glu242, Ser258, His259, Phe263, Lys281, Lys282, and Lys285.

RNA-binding proteins (RBPs) have often been considered undruggable targets. Developing small molecule inhibitors of RBPs can indeed be challenging and ADARs present their own challenges; however, targeting of RBPs and the interface of those proteins with RNA have shown therapeutic potential [23]⁠. For example, small molecules have been identified that inhibit the Lin28–let-7 interaction [24,25]⁠. The identified compounds bind Lin28, an RBP, inhibiting Lin28 binding of let-7 microRNA, an identified tumor suppressor whose maturation is inhibited by Lin28. Within multiple human cancer cell lines, one identified compound, 1632, was capable of inhibiting Lin28 by blocking binding to let-7 microRNA and led to decreased cancer cell proliferation and stemness [25]⁠. Structural characterization of the RBP-RNA binding interface is the most indispensable data prior to development of small molecule inhibitors targeting an RBP-RNA interaction. Each ADAR consists of at least two dsRBDs and a catalytic deaminase domain (CDD) [26]⁠. The CDD of ADAR1 and ADAR2 is the domain that contains the active site for the catalytic deamination of A-I [27]⁠. Mutagenesis experiments have determined that catalytically inactive forms of ADAR1 and ADAR2 also have RNA editing-independent functions that occur through protein–protein interactions, and protein–RNA interactions [28,29,30]⁠. The dsRBDs of ADAR proteins contribute to ADAR subcellular localization and are key for site-specific targeting of critical ADAR editing sites, allowing for strict regulation and meticulous editing of specific adenosines [31,32,33]⁠. ADAR2 contains two dsRBDs, whose structures have been captured by solution nuclear magnetic resonance (NMR) spectrometry [34,35]⁠. The dsRBDs of ADAR2 follow the topology α-β-β-β-α and there are three regions where the second dsRBD of ADAR2 contacts RNA. The first region includes helix α-1 that contacts the first minor groove, the second a highly conserved KKNAK motif, and the third the β1-β2 loop that interacts with the second minor groove of the RNA (Figure 1) [34]⁠. The α-1 helix and β1-β2 loop are key to mediating ADAR2 sequence-specific contacts [35]⁠. Mutagenesis studies targeting these regions result in significantly decreased editing of ADAR2 targets [35]⁠. Mutations within the β1-β2 loop, namely S258A and H259A, that are close by the editing site, affect editing loss the most [35]⁠. The KKNAK motif primarily holds non-sequence specific contacts with the RNA backbone, but mutation of lysine residues (Lys281, Lys282, and Lys285) completely abolishes ADAR2 binding to long dsRNA [32]⁠. The EAA mutation tested in mesothelioma disturbed RNA interaction with these critical lysine residues resulting in the antitumor effects [22]⁠.

ADAR2 is a critical drug target not only for cancer therapeutics but for various therapeutic fields including neurological disorders, infectious diseases, autoimmune diseases, and inflammatory diseases [36]. The regulatory mechanisms of ADAR2 are therefore of interest for understanding how ADAR2 is controlled in a wide range of molecular mechanisms. The editing roles of ADAR2 clearly influence molecular mechanisms that are largely dependent on where in the RNA transcript editing occurs. Results of editing within the coding region can directly impact protein functionality, within introns can affect alternative splicing, and within the UTR can increase transcript degradation or translation [37]. Several studies have assessed ADAR2 editing-dependent functions by looking at catalytically inactive ADAR2 mutants or via *ADAR2^−/−^/Gria2^R/R^* mice [30,38]. These studies benefit from being able to monitor the knockout and recovery of editing on sites of interest. These studies support the critical nature of ADAR2 editing in the brain; for example, loss of GluA2 editing is ultimately lethal, but survival can be altered with editing recovery [38]. Studies addressing the editing-independent mechanisms of ADAR2 are much less prevalent. Still, mechanisms by which ADAR proteins form dimers or complexes with other proteins or RNA can influence protein expression levels [39,40,41]. For example, ADAR2 binding to Cat2 transcribed nuclear RNA (Ctn RNA) acts to stabilize the RNA transcript and block association with other destabilizing RBPs [40]. In addition, ADAR dimerization is a hot topic of discussion for ADAR regulation. ADAR dimerization can impact editing efficiency [41]. Structures of an ADAR2 asymmetric dimer have been captured; however, these structures utilize ADAR monomers that only include the dsRBD2 and CDD and lack the first dsRBD of ADAR2 [41]. It is uncertain how incorporation of the full-length ADAR protein may alter potential binding interfaces. Mutants or altered isoforms may disturb dimerization or other protein–protein or protein–RNA interactions with physiological consequences. The EAA mutant in mesothelioma is an example of a context where RNA-binding deficiency rather than RNA-editing deficiency is implicated in cancer progression [22]. Full-length ADAR2 models could be used for further protein–protein modeling to assess ADAR dimerization or important ADAR–protein complexes. Identification of new ADAR interfaces will present new target sites for novel therapeutic development. Studies assessing the RNA editing-independent mechanisms or complex dependent editing/binding of ADAR would be greatly aided by both small molecules that can inhibit RNA binding and by full-length ADAR2 models.

This study will screen a natural compound library for compounds that target the RNA binding interface of ADAR2’s second dsRBD. Natural compounds have been used to modulate the activity of other RBPs; for example, azaphilone-9 targeting HuR [23]⁠. Top compounds identified here represent lead compounds with predicted safety and ability to bind the second dsRBD-RNA interface with high affinity implicated for mesothelioma treatment. In addition, there have been no full-length structures published of the ADAR2 protein; however, individual domains of ADAR2 have been determined by NMR and X-ray crystallography. Several key questions about ADAR proteins currently focus on dimerization and potential protein–protein interfaces that are involved in the many mechanisms ADAR proteins influence. Using computational modeling methods, we predict a complete ADAR2 model for further examination of ADAR2 interactions.

## 2. Results and Discussion

### 2.1. Molecular Docking to the ADAR2 dsRBD2-GluR-2 Interface

Although no prior compounds have been identified to bind the dsRBD of ADAR proteins, the three interaction regions between the 2dsRBD and GluR-2 RNA have been identified (Figure 1). From the TCM catalogue, 25,122 compounds were docked to the interface between the ADAR2 2dsRBD and the GluR-2 RNA substrate. Of these compounds, 211 had a docking score ≤ −7.0 kcal/mol. The lowest docking score was compound ZINC000085569217 at −8.1 kcal/mol (Table S1). Other top compounds reported from docking included ZINC000070450887 and ZINC000085594944 at −8.0 kcal/mol, and ZINC000085633079 at −7.9 kcal/mol (Table S1).

### 2.2. Physicochemical Properties and Safety Profiling

The 211 compounds meeting the −7.0 kcal/mol threshold then underwent ADME screening with SwissADME and were evaluated for their drug-likeness. Veber’s rule and Lipinski’s rule of five are guidelines for the prediction of drug oral bioavailability [42,43]⁠. Veber’s rule evaluates rotatable bonds, polar surface area, and hydrogen bond donors and acceptors that contribute to the prediction of a compound’s ability to reach circulation following oral administration [42]⁠. During this screen, compounds with violations to Veber’s rule were removed from consideration; 57 compounds failed Veber’s rule. Lipinski’s rule of five supports other thresholds that strong drug candidates are likely to meet, allowing the removal of compounds that may be poorly absorbed [44]⁠. Lipinski’s rule of five assesses molecular weight, lipophilicity, and hydrogen bond donors and acceptors [45]. No more than one violation to Lipinski’s rule of five was permitted; 49 compounds failed Lipinski’s rule. In total, 66 compounds failed both rules, and 138 compounds passed this screening. Compounds ZINC000085569217 and ZINC000070450887 with the lowest docking scores failed the ADME screen. Of the 138 compounds that passed the ADME screen, compounds ZINC000085594944 and ZINC000085633079 had the lowest docking scores at −8.0 kcal/mol and −7.9 kcal/mol, respectively. Additional pharmacokinetic property predictions for selected compounds are reported including blood–brain barrier (BBB) permeance, gastrointestinal (GI) absorption, and estimated solubility (ESOL) class (Table 1). ADAR2 is highly prevalent in the brain; however, mesothelioma primary tumor sites occur dominantly in the pleura, peritoneum, and pericardium [46]. Overexpression of ADAR2 within mice has displayed dysfunction in the lungs [47]. In particular, the mice have a significant increase in neutrophil infiltration, disorganization of the alveoli, and pulmonary fibrosis [47]⁠. Interestingly, inhalation of asbestos also results in pulmonary fibrosis which may be aggravated by the overexpression of ADAR2 [47,48]⁠. For delivery to the lungs, oral inhalation may be considered. Between oral bioavailability and oral inhalation, some physicochemical properties are shared. These include properties that affect permeability such as low molecular weight (<500 g/mol), lipophilicity (a logP between 1 and 5), and absorption potentials (low hydrogen bond capacity and low polar surface area).

Following the ADME screen, the 138 compounds then underwent toxicity prediction using OSIRIS DataWarrior v5.5.0. The compounds were evaluated using their chemical structure to predict toxicities related to mutagenicity, tumorigenicity, irritancy, and reproductive effects. Potential toxicity risks were classified as either none, low, or high. Compounds reporting any predicted toxicity related to mutagenicity, tumorigenicity, or irritancy were excluded from further consideration. A total of 91 compounds passed the toxicity screen with 60 compounds passing with no predicted toxicities and 47 compounds failed (Table S2). Predictions of mutagenic risks showed 10 compounds with high risk, 8 compounds with low risk, and 120 compounds with no predicted mutagenic risk (Table S3). Predictions of tumorigenic risks showed 12 compounds with high risk, 7 compounds with low risk, and 119 compounds with no predicted tumorigenic risk (Table S3). Predictions of irritancy risks showed 32 compounds with high risk, 2 compounds with low risk, and 104 compounds with no predicted mutagenic risk (Table S3). Predictions of reproductive effects resulted in 34 compounds with high risk, 12 compounds with low risk, and 92 compounds with no predicted reproductive risk (Table S3). Following the toxicity screen, the top 14 compounds had docking scores between −8.0 and −7.5 kcal/mol (Table 2).

### 2.3. Protein–Ligand Molecular Dynamics Simulations and Free Binding Energy Calculations

The protein–ligand complexes of the shortlisted compounds were then visualized to ensure proper localization to the dsRBD2-GluR-2 (protein–RNA) interface. Successful complexes then underwent 100 ns MD simulations; this included 11 total complexes and the dsRBD2 unbound. Compounds evaluated were ZINC000085594944, ZINC000085633079, ZINC000003203078, ZINC000034512861, ZINC000085597263, ZINC000095909822, ZINC000014649947, ZINC000044305204, ZINC000070454124, ZINC000085532515, and ZINC000085633008. These initial protein–ligand simulations aimed to look at the interactions between the selected ligands and the dsRBD2 at the atomic level allowing insights into the dynamics and stability of the complexes. Use of protein–ligand MD simulations has been a valuable tool for computer-aided drug design and discovery by reducing requirements for costly experimental screens and time to develop leads with desired effects [49]⁠. In addition to complex stability and dynamics, these simulations provided data for calculations of the energetics of the complexes.

#### 2.3.1. RMSD Evaluation for dsRBD2–Ligand Complexes

The RMSD was assessed during the 100 ns MD simulations to quantify the structural stability of the protein in the protein–ligand complexes. The RMSD over time was visualized using a RMSD plot (Figure S1a). RMSD plots help visualize the deviations of the protein backbone from the initial pose over time. Protein–ligand complexes with limited fluctuations of the protein backbone, i.e., RMSD values less than 3 Å, support stability of the protein and convergence of the structure [50]. All of the dsRBD2–ligand complexes had a lower average RMSD than the unbound dsRBD2 supporting the stability of the complexes (Table S4). Complex with ZINC000014649947 had the lowest average RMSD at 0.2358 nm (Table S4).

#### 2.3.2. Rg Evaluation for dsRBD2–Ligand Complexes

Rg was evaluated comparing the unbound and bound forms of the dsRBD2. Rg can help assess the impacts of ligand binding. A decrease in Rg may represent increased compaction whereas an increase in Rg upon ligand binding may represent protein unfolding or ligand-induced expansion. The unbound dsRBD2 had the lowest average Rg at 1.225 nm (Table S4). All of the protein–ligands had comparable Rg to the unbound protein and stayed relatively consistent, suggestive of stable, folded structures (Table S4 and Figure S1b). Compounds ZINC000085597263, ZINC000085633079, and ZINC000095909822 had the lowest Rg values and presented the most stable complexes (Table S4 and Figure S1b).

#### 2.3.3. RMSF Evaluation for dsRBD2–Ligand Complexes

RMSF measures the deviation of individual atoms. Looking at the RMSF plot allows for the observation of residues with high flexibility or regions with more dynamics. From RMSD, unbound dsRBD2 had an RMSD above the 3 Å threshold expected for a highly stable structure. There are two regions of variation observed for the unbound dsRBD2 by RMSF. The first region is from residue ~170 to 180 and the second is from residues 195 to 200 (Figure S1c). Compared to the holo forms of dsRBD2, these two regions have higher flexibility in the apo form suggesting that ligand binding induces stability of these flexible regions. These two regions are both loop regions of the dsRBD2 (Figure 1). Notably, in the holo complexes, a region containing the β1-β2 loop between residues 180 and 190 has a spike in fluctuation (Figure S1c). However, the regions containing the residues for the α1 helix, residues 164–169, interactions residue Phe190, and the region containing the KKNAK motif, residues 208–212, have low fluctuations indicative of their involvement in binding (Figure S1c).

#### 2.3.4. Free Binding Energy Calculations for the dsRBD2–Ligand Complexes

Binding free energy calculations are a useful tool for predicting and ranking better drug candidates. Rather than docking score alone, MM/PBSA calculations allow for more accurate computational predictions of binding affinities between protein–ligand complexes [51]. In particular, for each protein–ligand complex several important enthalpic contributions to binding affinity including van der Waals, electrostatic energy, polar solvation energy, and solvent-accessible surface area (SASA) were calculated using g_mmpbsa tool (Table 3).

From the compounds with favorable binding free energies, top compounds had free energies between −66.133 and −136.667 kJ/mol (Table 3). The compound with the most negative free energy and so the predicted strongest binding affinity for the dsRBD2 was ZINC000085633079 at −136.667 kJ/mol (Table 3). Other strong binders included ZINC000085597263 and ZINC000034512861 at −115.873 and −117.174 kJ/mol, respectively (Table 3). Three compounds, ZINC000003203078, ZINC000044305204, and ZINC000085532515, resulted in positive free energies (Table 3). Looking at snapshots from the 100 ns MD simulations, compounds ZINC000003203078 and ZINC000085532515 each leave the binding pocket. Compound ZINC000003203078 exits the pocket by 25 ns and compound ZINC000085532515 exited the binding pocket after 75 ns but before 100 ns. Due to the predicted unfavorable binding energy, these compounds were not considered further.

### 2.4. RNA–Protein–Ligand Simulations

A 100 ns MD simulation of the protein–RNA (dsRBD2-GluR2) was carried out to compare with the RNA–protein–ligand simulations. Complexes with low binding-free energies from the protein–ligand complexes were further subjected to 100 ns simulations in the presence of ADAR2 RNA substrate GluR-2. GluR-2 substrate contains a key R/G editing site that is highly edited within the central nervous system and has been well studied [35]. The presence of RNA substrate can significantly alter a protein–ligand interaction by influencing the ligand-binding affinity, altering the conformational stability, or dynamic behavior of the protein. The compounds identified target the ADAR2 dsRBD2-GluR-2 interface so we wanted to assess the competition between the RNA and ligand for the binding site. In addition, understanding the effects of RNA presence on the lead dsRBD2–ligand interactions is crucial for further drug development. The MD simulations allowed a visualization of the dynamics of the RNA–dsRBD2–ligand systems and the stability and interactions of these systems were further evaluated by RMSD, Rg, RMSF, hydrogen bonding, and energy calculations.

#### 2.4.1. RNA–Protein–Ligand RMSD Analysis

**Figure 2:**
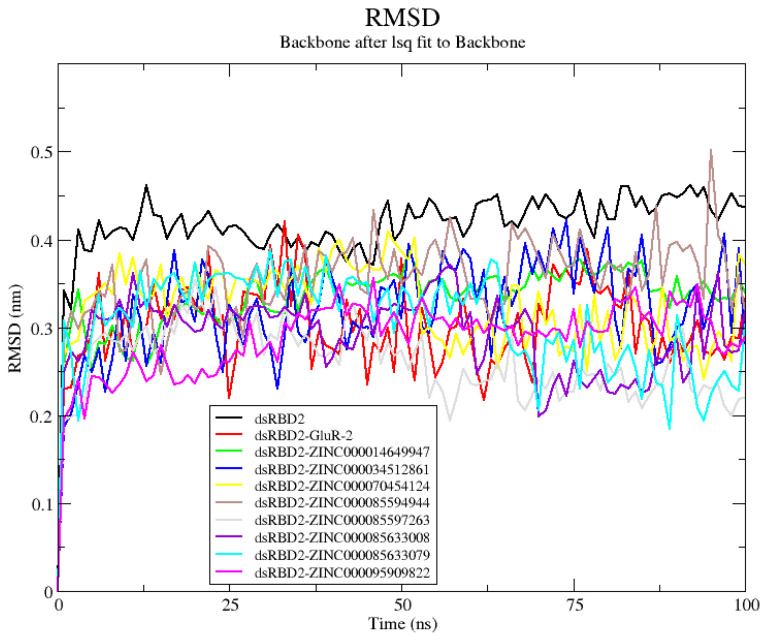
Plot of RMSDs from 100 ns RNA–protein–ligand MD simulations. Includes eight protein–ligand complexes with dsRBD2, dsRBD2-GluR-2 (protein–RNA), and dsRBD2 (unbound protein).

**Figure 3:**
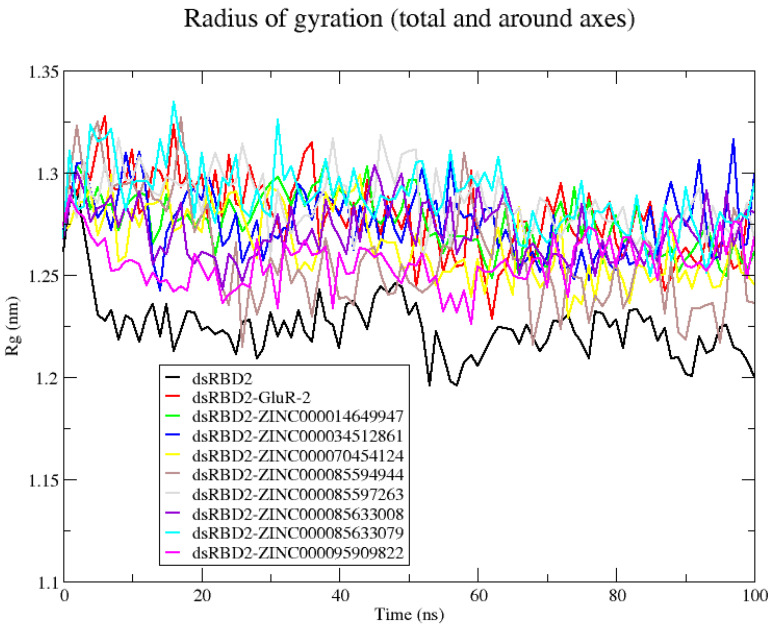
From RNA–protein–ligand MD simulations, Rg plot of dsRBD2–ligand complexes, dsRBD2-GluR-2 complex, and dsRBD2 unbound.

The average RMSD of the dsRBD2 bound to GluR-2 RNA was 0.3008 nm and this was lower than that of unbound dsRBD2 at 0.4158 nm (Table S5). All eight complexes assessed had lower RMSDs than that of unbound dsRBD2 (Figure 2 and Table S5). Of the eight complexes, complexes with ZINC000085597263, ZINC000085633008, and ZINC000095909822 had RMSD lower than the dsRBD2-GluR-2 complex and were under 3 Å, suggestive of good stability. The dsRBD2-ZINC000085597263 complex had the lowest RMSD values with an average RMSD of 0.2591 nm (Figure 3 and Table S5). Complexes ZINC000085633008 and ZINC000095909822 also had low RMSD values with averages of 0.2908 nm and 0.2863 nm, respectively (Table S5).

#### 2.4.2. RNA–Protein–Ligand Rg Analysis

The unbound dsRBD2 had the lowest Rg indicative of the most compact structure; this tends to be associated with more native-like folding and high stability (Figure 3). The dsRBD2 in the complex with GluR-2 had comparable Rg with an average Rg of 1.279 nm (Table S5). All of the eight compounds produced Rg comparable to the protein–RNA complex (Figure 3). Several complexes had average Rg lower than the protein–RNA complex, including ZINC000014649947, ZINC000034512861, ZINC000070454124, ZINC000085594944, ZINC000085633008, and ZINC000095909822 complexes (Table S5). The complexes with the lowest Rg were ZINC000085594944 and ZINC000095909822 with Rg averages of 1.259 nm and 1.256 nm, respectively (Figure 3 and Table S5).

#### 2.4.3. RNA–Protein–Ligand RMSF Analysis

**Figure 4:**
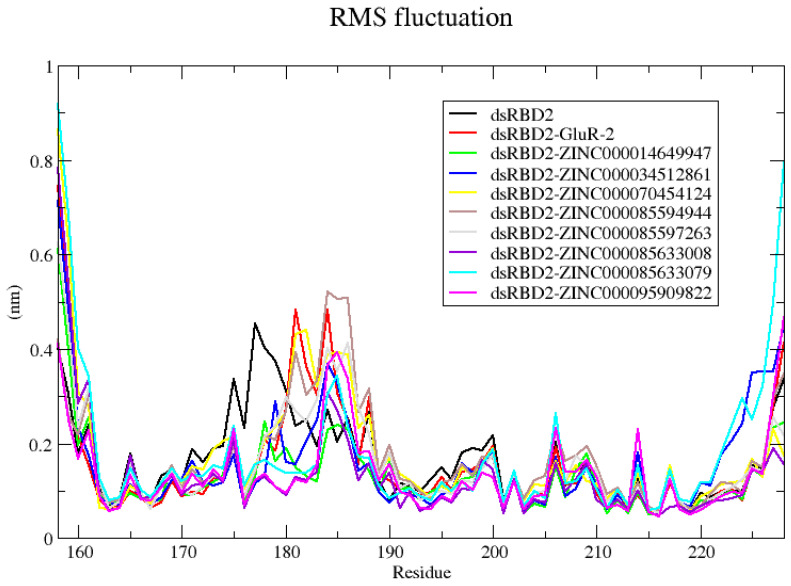
From RNA–protein–ligand MD simulations, RMSF plot of dsRBD2–ligand complexes, dsRBD2-GluR-2 complex, and dsRBD2 unbound.

For the apo dsRBD2 structure, the RMSD average of 0.4158 nm is above the 3 Å threshold expected for a highly stable protein (Table S5). Notably, the Rg observed remained the most tightly compacted (Figure 3). From RMSF, the areas with the highest fluctuation are specifically two areas with loop regions (Figure 4). The largest fluctuation is observed between residues 170–185 (Figure 4). These residues correspond to β1 sheet and the β1-β2 loop. The localized movement of this loop likely contributed to the increase in RMSD, but did not affect the overall compactness of the dsRBD, so the Rg stays low due to the stability of the overall packing. The other region of moderate fluctuation is from residues 193–200 which is a portion of loop between β3 and α2 (Figure 4). The complex with ZINC000014649947 had little fluctuation indicating a highly stable complex (Figure 4). Complexes with GluR-2, ZINC000085597263, ZINC000070454124, and ZINC000085594944 each had fluctuation around residues 180–190 (Figure 4). Complexes with ZINC000034512861, ZINC000085633079, ZINC000085633008, and ZINC000095909822 had fluctuation between residues 183 and 187 (Figure 4). These regions both include the β1-β2 loop, either the full loop or just the end of the loop. Overall, the β1-β2 loop appears to be a highly flexible region. RBPs often exhibit high flexibility and dynamics which are necessary for their function and can contribute to recognition and interaction with RNA targets [52].

#### 2.4.4. Free Binding Energy Calculations of the RNA–Protein–Ligand Complexes

The presence of RNA substrate GluR-2 can disturb protein–ligand interactions. Data from the RNA–dsRBD2–ligand MD simulations were used to calculate energy contributions identifying compounds predicted to bind strongly to the dsRBD2 even in the presence of the RNA substrate. Compound ZINC000003203078 reported a positive binding free energy during protein–ligand MD simulation and was removed from consideration as a lead compound but was checked during protein–ligand–RNA MD simulation to confirm that the binding free energy would remain negative in the presence of RNA substrate (Table 4). The GluR-2-dsRBD2 complex was used as a positive control, reporting a binding energy of −7011.460 kJ/mol (Table 4). Four compounds were predicted to bind to dsRBD2 in the presence of GluR-2 substrate (Table 4). The strongest binder was ZINC000085597263 with a predicted binding energy of −177.129 kJ/mol followed by ZINC000085633079 at −148.844 kJ/mol (Table 4). Compound ZINC000003203078 was predicted not to bind the dsRBD2 during protein–ligand evaluation; with the addition of GluR-2, the predicted binding energy was drastically unfavorable at 1377.422 kJ/mol (Table 4).

#### 2.4.5. Reproducibility of Top Four Compounds

For both the protein–ligand simulation and RNA–protein–ligand MD simulations, three technical replicates were used for the four lead compounds: ZINC000085597263, ZINC000085633079, ZINC000014649947, and ZINC000034512861, to assess the precision and repeatability of the results. In addition, compound ZINC000095909822 which was predicted to bind dsRBD2 during protein–ligand assessment but not remain bound in the presence of GluR-2, was also tested by three technical replicates. From the protein–ligand MD simulations, all four lead compounds ZINC000085597263, ZINC000085633079, ZINC000014649947, and ZINC000034512861 and ZINC000095909822 reliably reported negative binding energies and consistent results for RMSD and Rg (Tables S6 and S7). Averages for the RMSD values, Rg values, and hydrogen bonds during the 100 ns RNA–protein–ligand simulations are in Table S9. Using averages of the three technical replicates for RNA–protein–ligand MDs, the predicted binding energies for each of the four lead compounds were negative (Table S8). The presence of RNA influenced the binding of the ligands to protein. For compounds ZINC000034512861 and ZINC000014649947, there was a decrease in ligand-binding affinity for the dsRBD2. Compound ZINC000034512861 in the protein–ligand simulations had an average binding energy of −106.918 kJ/mol; however, in the presence of GluR-2, it significantly dropped to an average of −12.9827 kJ/mol (Tables S6 and S8). Compound ZINC000014649947 had an average binding energy of −93.6547 kJ/mol from protein–ligand simulations and binding affinity dropped to an average of −52.2367 kJ/mol during the RNA–protein–ligand simulations (Tables S6 and S8). In contrast, RNA presence increased the affinity of compounds ZINC000085633079 and ZINC000085597263 (Tables S6 and S8). During protein–ligand simulation, ZINC000085633079 and ZINC000085597263 had average binding energies of −127.1737 kJ/mol and −118.1693 kJ/mol, respectively (Table S6). The RNA–protein–ligand simulations had stronger affinities with −145.511 kJ/mol for ZINC000085633079 and −215.116 kJ/mol for ZINC000085597263 (Table S8). Compound ZINC000095909822 reliably reported negative binding affinities during protein–ligand simulation but a highly positive binding energy during RNA–protein–ligand simulation suggesting RNA greatly impacts the protein–ligand interactions of this compound (Tables S6 and S8).

### 2.5. Molecular Interactions

The four compounds ZINC000085597263, ZINC000085633079, ZINC000014649947, and ZINC000034512861 were calculated to remain bound to the dsRBD2 in the presence of known RNA substrate GluR-2. These compounds represent the strongest leads going forward for inhibition of dsRBD to RNA with implications for mesothelioma treatment. The compounds ZINC000070454124, ZINC000085594944, ZINC000085633008, and ZINC000095909822 were predicted to bind the dsRBD2 but did not maintain binding in the presence of RNA substrate. The protein–ligand interactions will be assessed by protein–ligand interaction plots (PLIP), per-residue energy contributions, and hydrogen bonding. Identifying the protein–ligand interactions between these eight compounds is a means to better understand how to increase the binding affinity of these ligands for better specificity. The changes in the protein–ligand interactions in the presence of RNA are also important because identifying interactions influenced by RNA substrate are residues that should be considered for further drug optimization.

#### 2.5.1. Protein–Ligand Interaction Plots (PLIP)

**Figure 5:**
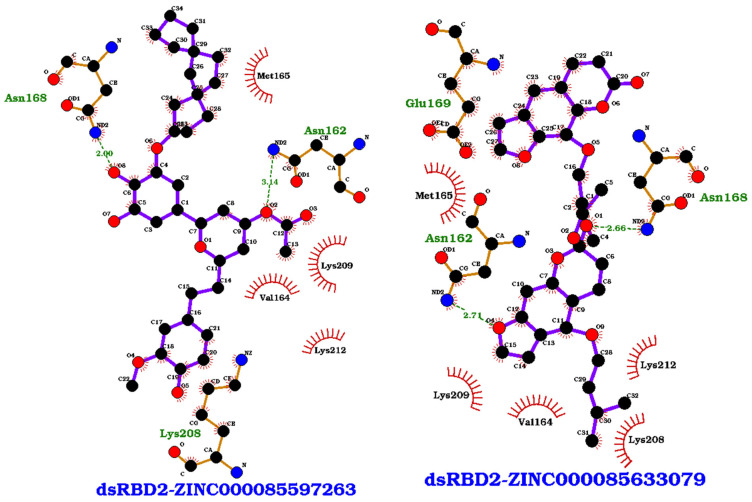
PLIP of compounds ZINC000085597263 and ZINC000085633079 with strongest predicted binding affinities. Black circles are carbons, red circles are oxygens, blue atoms are nitrogens, ligand bonds in purple, protein bonds in brown, hydrogen bonds are represented by dashed green lines, and hydrophobic bonds are red coronas.

Key residues observed in the protein–ligand interactions of the eight complexes include residues Val164, Met165, Asn168, Glu169, Lys208, Lys209, and Lys212; these are critical residues in the dsRBD2-RNA interaction (Figure 1 and Table 5). PLIPs for the eight complexes are provided in (Figure 5 and Figure S2). Small molecule binding would block these residues, interfering with dsRBD2 binding to RNA. Residue Lys209 was observed consistently in all eight of the protein–ligand interactions (Table 5). Val164 was present in all complexes but ZINC000014649947, and Lys208 was present in all complexes but ZINC000070454124 (Table 5). Key hydrogen bond compounds included Asn162 and Asn168 (Table 5). The PLIP of ZINC000085597263 and ZINC000085633079 were representative of the predicted two best binders (Figure 5). Both ZINC000085597263 and ZINC000085633079 bound to residues within the α1 helix (Val164, Met165, and Asn168) and the KKNAK motif (Lys208, Lys209, and Lys212), two of the three regions involved in RNA binding (Figure 5). ZINC000085633079 also had interactions with residues Asn162 and Glu169 (Table 5 and Figure 5). Other interaction residues included Pro172 in the ZINC000070454124 complex, Ala213 in both ZINC000014649947 and ZINC000085633008 complexes, and Leu210 in complexes ZINC000085633008 and ZINC000070454124 (Table 5). No compounds targeted residues Ser185 or His186 in the β1-β2 loop which is a highly flexible area identified from RMSF.

#### 2.5.2. Per-Residue Energy Contributions

**Figure 6:**
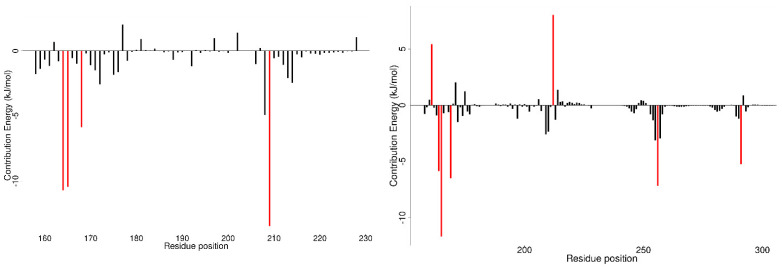
Per-residue energy contribution plots of ZINC000085633079. Left between protein–ligand and right between RNA–protein and ligand. Red bars contributed greater than 5.0 kJ/mol or less than −5.0 kJ/mol, black bars did not meet these thresholds.

The per-residue energy contributions were evaluated for both protein–ligand and RNA–protein–ligand simulations (Figure 6, Figures S3 and S4, Data S1). From MM/PBSA calculations, individual residue energy contributions could be quantified. Residues contributing above 5 kJ/mol either positively or negatively are considered important interactors. The residues contributing to the GluR-2 interaction are shown in Figure S4h and the exact energy contributions of each residue are listed in Data S1. Between GluR-2 and the dsRBD2, the experimentally identified critical residues reported energy contributions in kJ/mol as follows: Val163 (−14.7201), Met165 (−19.9771), Asn168 (−6.3131), Glu169 (883.4873), Ser185 (−1.8959), His186 (−3.0347), Phe190 (−10.1619), Lys208 (−903.6095), Lys209 (−1010.4625), and Lys212 (−929.7762) (Figure S4h and Data S1). Notably Lys208, Lys209, and Lys212 greatly contributed to binding affinity whereas residues Ser185 and His186 did not contribute above the ±5 kJ/mol threshold and residue Glu169 decreased the binding affinity.

From protein–ligand MD simulations, the key contributing residues in the majority of protein–ligand interactions were Val164, Met165, Asn168, and Lys209 (Figure S3 and Data S1). Assessing the per-residue energy contributions, RNA presence greatly decreased the binding affinity of compounds ZINC000085594944, ZINC000085633008, ZINC000070454124, and ZINC000095909822, which were the four compounds predicted to bind dsRBD2 and lost binding affinity in the presence of RNA. Specifically, for these four residues, bases in the RNA C290, C291, and C292 contributed greatly to the hindrance of ligand binding (Data S1). For example, for ZINC000070454124, RNA bases C290, C291, and C292 contributed 1019.7988, 1527.5529, and 28.3754 kJ/mol, respectfully (Figure S4c and Data S1). In contrast, ZINC000085633079, ZINC000014649947, ZINC000085597263, and ZINC000034512861 did not have large interactions with the RNA (Data S1).

From the protein–ligand simulations, compounds ZINC000085633079, ZINC000085597263, and ZINC000034512861 each had binding energies less than −100 kcal/mol (Table 3). These three compounds also maintained low binding energy during the RNA–protein–ligand simulations; therefore, the interacting residues of these compounds are of great interest. Critical compounds contributing to the binding energy in kJ/mol for ZINC000085633079 were Val164 (−10.6982), Met165 (−10.4330), Asn168 (−5.8570), and Lys209 (−13.4559) (Figure 6 and Data S1). The inclusion of RNA substrate altered some of the interaction residues and interactions with RNA were also described. An example of per-residue contribution maps for ZINC000085633079 highlight how the presence of RNA could alter and influence protein–ligand interactions (Figure 6). With the inclusion of RNA substrate, critical residues of the dsRBD2 for ZINC000085633079 became Lys161, Val164, Met165, Glu169, and Lys212 with energy contributions of 5.4243, −5.8503, −11.6759, −6.6150, and 8.0276 kJ/mol, respectively. In addition, interaction with GluR-2 RNA bases U-256 and C-291 contributed energies of −7.1725 and −5.2535 kJ/mol, respectively. Critical residues for ZINC000085597263 contributed energies in kJ/mol of Pro158 (−5.7992), Val164 (−5.8152), Met165 (−6.8187), Lys209 (−10.5241), Leu210 (−5.848), and Leu228 (6.1076). GluR-2 RNA did not inhibit ZINC000085597263 binding, and with residues C290, C291, and C292 actually contributed energies of −6.4758, −14.8362, and −5.0059 kJ/mol. Residues contributing to the ZINC000034512861 interaction included Pro158, Val164, Met165, Lys209, Leu210, and Leu228 with energies of −5.7992, −5.8152, −6.8187, −10.5241, −5.8480, and 6.1076 kJ/mol. With the presence of RNA, ZINC000034512861 had no residues contribute significantly to the interactions; however, numerous residues had small negative contributions and the RNA residues did not greatly decrease the binding affinity (Figure S4b and Data S1).

#### 2.5.3. Changes in Hydrogen Bonding

Hydrogen bonding (hbonds) is a crucial interaction between the protein and ligand that can greatly enhance the binding affinity. The presence of particular hydrogen bonds can also be key for ligand specificity and optimization of hbonds can increase ligand binding [53]. However, too many hbonds can reduce the permeability of a small molecule resulting in bad bioavailability [43]. In comparison between the protein–ligand and RNA–protein–ligand simulations, there is a reduction in hbonding. From the protein–ligand simulations, compounds ZINC000085633079 and ZINC000085597263 form four hbonds, and ZINC000095909822 forms three (Figure S5a). Other high bonding compounds include ZINC000085594944, ZINC000085633008, ZINC000014649947, and ZINC000070454124 that each make up to two hbonds during the 100 ns (Figure S5a). In the presence of GluR-2 RNA, several compounds lose hbonds (Figure S5b). ZINC000085597263 that made up to four hbonds during the protein–ligand simulation, makes up to one hbond during the RNA–protein–ligand simulation (Figure S5). ZINC000085633079 and ZINC000095909822 drop from maxes four and three to three and two, where each lose one hbond in the presence of RNA (Figure S5). Interestingly, ZINC000070454124 gains one hydrogen bond forming up to three hbonds in the presence of RNA (Figure S5b). Compounds ZINC000085594944, ZINC000085633008, and ZINC000014649947 maintain the ability to make up to two hbonds during the 100 ns (Figure S5b).

### 2.6. Lead Compound Characterization

Compounds ZINC000085597263, ZINC000085633079, ZINC000014649947, and ZINC000034512861 were predicted to remain bound to dsRBD2 in the presence of RNA substrate. To better characterize these four compounds, PASS was evaluated to predict therapeutic effects and structural similarities to assess identified key chemical features that may be involved in therapeutic activity.

#### 2.6.1. PASS, Biological Activity Prediction

PASS predicts a compound’s biological activity spectrum based on its structural features. The PASS results analyze the structure activity relationships (SARs) from a large database of known compounds and then compares identified patterns to predict the activity of the new compound. Using this SAR analysis, PASS identifies key functional groups associated with activity and then uses statistics to predict the likelihood of activity or inactivity where Pa is probability of activity and Pi is probability of inactivity [54]. During this evaluation, only Pa > 0.3 was evaluated.

Although ADAR2 can have tumor-suppressive effects, the overexpression and increased editing of ADAR2 has been identified in several types of cancer including mesothelioma, colorectal cancer, cervical cancer, breast cancer, neuroblastoma, lung carcinoma, and esophageal squamous cell carcinoma (ESCC) [21,55,56,57]. Only in mesothelioma has the disturbance of ADAR2 RNA binding been identified as a mechanism with antitumor affects [22]. Treatment for mesothelioma with early diagnosis involves surgery in combination with chemotherapy; however, there are issues with misdiagnosis related to disease rarity, the non-specificity of symptoms, and similarity to other diseases. Mesothelioma is an aggressive cancer with low survival rates particularly at 3 and 5 years post-diagnosis [58]. Numerous chemotherapy treatments have been tested covering a variety of molecular mechanisms to influence mesothelioma. First-line drugs include a combination of cisplatin, that induces DNA damage, and pemetrexed, that targets folate metabolism to block cell division and trigger apoptosis [59]. Other drugs under investigation for mesothelioma treatment have included methotrexate, a dihydrofolate reductase inhibitor; gemcitabine, a nucleoside analog; vinorelbine, a mitotic inhibitor; taurolidine, which impacts oxidative stress pathways in mesothelioma; vironostat, a histone deacetylase (HDAC) inhibitor; bevacizumab, an antiangiogenic agent; ipilimumab, a monoclonal antibody that targets human cytotoxic T-lymphocyte antigen 4 (CTLA-4); ramucirumab, an anti-vascular endothelial growth factor receptor (VEGFR)-2 antibody; and doxorubicin, which targets topoisomerase II and induces DNA damage [60,61,62]. Overall, mechanisms involving inflammation, DNA damage, DNA replication, cell growth, and metastasis are critical to mesothelioma and targets of current drugs for mesothelioma treatment; because of this, the lead compounds were evaluated for their potential to participate in these mechanisms (Tables S10, S12, S14 and S16).

All four compounds were predicted to have anticancer activities (Tables S10, S12, S14 and S16). ZINC000014649947 was predicted as a preneoplastic conditions treatment (Pa: 0.757 and Pi:0.005), antimutagenic (Pa: 0.628 and Pi:0.009), anticarcinogenic (Pa: 0.340 and Pi: 0.045), and antineoplastic (non-Hodgkin’s lymphoma) (Pa: 0.308 and Pi: 0.213). ZINC000034512861 was predicted as an antineoplastic (Pa: 0.922 and Pi: 0.005), chemopreventive (Pa: 0.915 and Pi: 0.002), antimetastatic (Pa: 0.540 and Pi: 0.012), prostate cancer treatment (Pa: 0.524 and Pi: 0.008), and anticarcinogenic (Pa: 0.310 and Pi: 0.051). ZINC000034512861 was predicted to be an antineoplastic for numerous types of cancer including melanoma, carcinoma, lung, breast, colon, colorectal, ovarian, thyroid, endocrine, cervical, and liver cancers with Pas of 0.680, 0.553, 0.826, 0.742, 0.735, 0.734, 0.726, 0.634, 0.603, 0.552, and 0.324, and corresponding Pis of 0.004, 0.004, 0.004, 0.005, 0.005, 0.005, 0.004, 0.001, 0.002, 0.005, and 0.014. ZINC000085597263 was predicted as a chemopreventive (Pa: 0.805 and Pi: 0.004), antineoplastic (Pa: 0.623 and Pi: 0.040), a prostate cancer treatment (Pa: 0.433 and Pi: 0.017), and anticarcinogenic (Pa: 0.328 and Pi: 0.048). In addition, ZINC000085597263 was also predicted as a neoplastic for breast cancer, lung cancer, carcinoma, and non-Hodgkin’s lymphoma with Pas 0.559, 0.486, 0.313, and 0.383 and corresponding Pis of 0.014, 0.014, 0.013, and 0.124. ZINC000085633079 was predicted as an antineoplastic enhancer (Pa: 0.997 and Pi: 0.001), antineoplastic (Pa: 0.927 and Pi: 0.005), chemopreventive (Pa: 0.651 and Pi: 0.008), antimetastatic (Pa: 0.382 and Pi: 0.052), and antileukemic (Pa: 0.330 and Pi: 0.037).

The four compounds each had predicted activities that matched with current therapeutics targeting mesothelioma (Tables S10, S12, S14 and S16). Blockade of DNA synthesis is a common strategy for cancer therapeutics. The combination of cisplatin–pemetrexed induces DNA damage and blocks the metabolism necessary for proper repair leading to apoptosis. Pemetrexed inhibits folate metabolism impairing thymidine and purine synthesis, and downstream methionine generation involved in proliferation [63]. Methylenetetrahydrofolate reductase is involved in folate and methionine metabolism, and its inhibition has reported decreased tumor proliferation in vivo [63]. ZINC000014649947 was predicted to inhibit methylenetetrahydrofolate reductase with a Pa of 0.597 and Pi of 0.038. ZINC000014649947 was also predicted to inhibit formate-dihydrofolate ligase and thymidylate 5′-phosphatase with Pas of 0.525 and 0.401 and Pis of 0.009 and 0.053, respectively. Vinorelbine uses another mechanism to block cell proliferation by inhibiting microtubule formation and blocking mitosis. Compound ZINC000085597263 was predicted as a microtubule formation inhibitor with Pa 0.308 and Pi 0.014.

Oxidative stress and chronic inflammation are also involved mechanisms in mesothelioma progression. ROS and RNS are involved in asbestos toxicity influencing inflammation and immune recruitment in mesothelioma [64]. However, while increased ROS is implicated in mesothelioma pathogenesis, treatment with cisplatin–pemetrexed leads to increased ROS and triggers cell death with a blockade of DNA synthesis, DNA repair, and DNA replication [64]. Glutathione (GSH) is involved in cell protection from oxidative stress and chemical stress by breaking down and removing harmful metabolites or substances. Glutathione S-transferase (GST) catalyzes GSH conjugation to electrophiles. In mesothelioma, GST interferes with activation of pro-apoptotic pathways by MAPK, commonly triggered by cisplatin–pemetrexed therapy, and high expression of GST and export pumps increase drug removal also decreasing the efficacy of cisplatin–pemetrexed therapy [65]. ZINC000014649947 was predicted to inhibit glutathione thiolesterase, which catalyzes the reaction to generate GSH, with a Pa of 0.749 and Pi of 0.012. Glutathione peroxidase-1 (GPX-1) is elevated in malignant pleural mesothelioma and induces GSH oxidation and reduced H_2_O_2_ [66]. ZINC000014649947 was predicted as a peroxidase inhibitor with Pa 0.741 and Pi 0.008. Auranofin is a thioredoxin inhibitor that increases oxidative stress, raising ROS levels, but decreases GSH in mesothelioma to cause apoptosis [67]. Compound LCS3 is a thioredoxin reductase 1 and glutathione disulfide reductase inhibitor that impacts oxidative stress pathways to induce apoptosis and has shown antitumor efficacy in lung adenocarcinoma cells [68,69]. ZINC000014649947 and ZINC000034512861 were predicted to inhibit protein–disulfide reductase (glutathione) with Pas 0.646 and 0.429 and Pis 0.029 and 0.105. ZINC000014649947 was also predicted to inhibit thioredoxin (Pa: 0.646 and Pi: 0.019), glutathione-disulfide reductase (Pa: 0.359 and Pi: 0.012), and glutathione dehydrogenase (ascorbate) (Pa: 0.344 and Pi: 0.038).

Angiogenesis is an identified mechanism for tumor progression in mesothelioma. In mesothelioma, there is abnormal vasculature that results in hypoxia-promoting tumor cell survival, cell motility, and cell invasiveness [70]. Hypoxia signaling upregulates VEGF, other growth factors, and pro-angiogenic factors that can queue mesothelial cell growth and increase cancer cell migration [70]. Mesothelial cells can be sensitive to VEGF inhibition; bevacizumab is an anti-VEGF-A monoclonal antibody that has reported efficacy as a combination therapy with cisplatin and pemetrexed [71]. ZINC000014649947 was predicted as a steroid *N*-acetylglucosaminyltransferase inhibitor with a Pa of 0.787 and Pi of 0.003). β1,6-*N*-acetylglucosaminyltransferase increased VEGF release in colon cancer cells and in vivo induced angiogenesis [72].

Some molecular pathways have been explored for cell survival of mesothelioma cells in spite of increased oxidative stress and DNA damage. Caspase 3 and 8 are important to apoptotic induction in mesothelioma cells [73,74]. ZINC000014649947, ZINC000034512861, and ZINC000085597263 were predicted to stimulate caspase 3 with Pas 0.452, 0.946, 0.312 and corresponding Pis of 0.036, 0.003, and 0.114. ZINC000014649947, ZINC000034512861, and ZINC000085597263 were also predicted to stimulate caspase 8 with Pas 0.497, 0.874, and 0.424 and corresponding Pis of 0.018, 0.001, and 0.040. All four compounds, ZINC000014649947, ZINC000034512861, ZINC000085597263, and ZINC000085633079, were predicted as apoptosis agonists with Pas 0.407, 0.911, 0.479, and 0.422, and corresponding Pis of 0.070, 0.004, 0.044, and 0.063, respectively. Tumor necrosis factor α (TNF-α) for example decreases cytotoxicity of asbestos and increases cell survival through NF-κB, overall increasing the number of damaged cells with greater malignant potential [75]. ZINC000014649947 was predicted to inhibit TNF expression with a Pa of 0.483 and a Pi of 0.033. Compound ZINC000085597263 was predicted as a free radical scavenger (Pa: 0.430 and Pi: 0.015). Free radical scavengers and antioxidants may be good chemopreventives for mesothelioma; for example, LGM2605, both a free radical scavenger and antioxidant, can abate asbestos-induced ROS generation and cell damage in peritoneal macrophages [76].

#### 2.6.2. Structural Similarity Search

To further support the potential biological activities of the identified compounds, a structural similarity search was performed to identify known molecules with similar chemical structures and established activities. This approach uses concepts of the similarity-property principle that is based on molecular interactions being dependent on compound shape and functional groups and that chemical properties drive biological activities. The similarity of compounds assessed was greater than 60% (Tables S11, S13, S15 and S17).

Compound ZINC000014649947 was most similar to 5-pentyl-2-phenoxyphenol (DB07178), an anti-tuberculosis drug with a similarity score of 0.785. The next most similar structures were 3-(3,4-dimethoxyphenyl)propanoic acid (DB04208) and terameprocol (DB12226) with similarity scores of 0.653 and 0.652. 3-(3,4-dimethoxyphenyl)propanoic acid is a thalidomide analog indicated for biliary cirrhosis. Few cases of mesothelioma progressing to the liver have been reported, but one case did report biliary tract obstruction [77,78]. Terameprocol downregulates survivin, a mitosis regulator, and CDK1 transcription blocking cell division and inducing apoptosis in non-small cell lung carcinoma [79]. Inhibition of survivin has been shown to increase mitotic arrests and radiosensitivity in mesothelioma cells [80]. Other compound similarities included CA4P (DB05284), combretastatin (DB12596), and zingerone (DB15589) with similarities of 0.644, 0.644, and 0.640. Combretastatin is an anti-tubulin drug that disturbs angiogenesis and tumor blood flow and has reported antitumor activity in mesothelioma cell lines [81]. From combretastatin a water-soluble derivative, Combretastatin A-4 Phosphate (CA4P), was developed. CA4P in vivo leads to reduced tumor blood flow and the induction of tumor necrosis and it has been used in combination treatment with cisplatin [82]. CA4P is anticipated for phase II/III trials for ovarian cancer using a triple combination that includes bevacizumab [83]. Zingerone has shown anticancer activities including suppression of angiogenesis, decreased cell proliferation, and inhibited cell invasion and metastasis [84,85,86]. In mesothelioma cell line H2452, zingerone was capable of inhibiting cell migration and colony formation, but to a lesser extent than other cancer types [84]. Additionally, ZINC000014649947 had some similarity to 3,4,5-Trimethoxyamphetamine (DB01516), 5-[2-(4-hydroxyphenyl)ethyl]benzene-1,3-diol (DB08466), and secoisolariciresinol (DB12179 with similarity scores of 0.634, 0.631, and 0.626. Secoisolariciresinol diglucoside is being investigated as a chemopreventive of mesothelioma acting to decrease damage and inflammation pathway cascades resulting from asbestos exposure in both human mesothelial cells and mice [87,88]. Both flaxseed and purified secoisolariciresinol diglucoside were also shown to have some antitumor effects in breast cancer [89].

Compound ZINC000034512861 had similarity scores greater than 70% with numerous compounds used for the treatment of breast and prostate cancer (Table S13). Hormone therapies are being explored for mesothelioma treatment, particularly antiestrogen therapies. There is crosstalk between androgen receptor and estrogen receptor pathways, and both can influence the PI3K/Akt/mTOR pathway [90]. In mesothelioma, estradiol results in PI3K/Akt signaling cascade increasing tumor cell growth, survival, and metabolism [91,92]. Targeting of aromatase CYP19A1 by aromatase inhibitor exemestane decreases estrogen synthesis and can inhibit malignant mesothelioma tumor growth [93]. Few case reports support the use of antiestrogens for mesothelioma treatment [93,94]. Additionally, progesterone can also influence mesothelioma cells, decreasing cell proliferation and inducing apoptosis [95]. Conflictingly, estrogen receptor β (ERβ) agonists can alter mitochondrial oxidative phosphorylation, decrease cell proliferation, increase apoptosis, and increase sensitivity to cisplatin treatment in mesothelioma [96,97]. Aromatase inhibitors can suppress estrogen levels but increase testosterone levels. Testosterone or testosterone replacement can indirectly activate ERβ via aromatization. Unfortunately, the impacts of testosterone levels and androgen receptor levels on mesothelioma pathology is not documented; however, due to the crosstalk between androgen and estrogen pathways, compounds that overall decrease estradiol or increase ERβ activation may be implicated in mesothelioma combination therapy.

ZINC000085597263 had similarity to ERβ agonist erteberel (DB07933) with a score of 0.618. ZINC000034512861 was most similar to trestolone acetate (DB13958), a male contraceptive, with a similarity score of 0.776. ZINC000034512861 had a similarity score of 0.761 with terpinyl acetate (DB15957); this compound is from *Boswellia serrata* whose extract has anticancer activity and is also used for inflammatory diseases [98]. The next similar structures included testosterone propionate, and acetoxolone (DB13540) with similarity scores of 0.758 and 0.754. Several compounds shared the similarity score 0.74 with ZINC00003451286 including nandrolone decanoate (DB08804), testosterone cypionate (DB13943), testosterone enanthate (DB13944), testosterone undecanoate (DB13846), testosterone decanoate (DB16001), testosterone isocaproate (DB16002), and dimethandrolone undecanoate (DB16141), all of which are used for testosterone replacement or male contraception. Additionally, ZINC000034512861 had structural similarity with synthetic progesterone medroxyprogesterone acetate (DB00603), a score of 0.724, and with synthetic progestins norgestomet (DB11440) and drospirenone (DB01395) with scores 0.724 and 0.708, respectively.

ZINC000085597263 was also structurally similar to (3AS,4R,9BR)-4-(4-HYDROXYPHENYL)-6-METHOXYMETHYL)-1,2,3,3A,4,9B-HEXAHYDROCYCLOPENTA[C] CHROMEN-8-OL (DB08020), (3AS,4R,9BR)-4-(4-HYDROXYPHENYL)-1,2,3,3A,4,9B-HEXAHYDROCYCLOPENTA[C]CHROMEN-9-OL (DB08737), and Epigallocatechin gallate (DB12116) with similarity scores of 0.606, 0.602, and 0.601, respectively. These three compounds are polyphenols and flavonoids. Polyphenols are indicated as potential chemopreventive in mesothelioma due to their abilities to alter inflammatory pathways and ROS related to asbestos damage [99]. Flavonoids have strong implications for therapeutic development in oncology due to their abilities for free radical scavenging, to modulate immune response, trigger apoptosis, and trigger cell death pathways [100]. Epigallocatechin gallate (EGCG) is an antioxidant found in green tea that affects multiple pathways in cancer including cell proliferation, angiogenesis, metastasis, and angiogenesis [101]. In mesothelioma cells, EGCG decreased cell growth and targeted oxidative pathways promoting cell death [102,103].

Compound ZINC000085633079 had the highest similarity with Elsamitrucin (DB05129) with a score of 0.64. Elsamitrucin is an antitumor antibiotic that induces single strand breaks in DNA and then inhibits topoisomerases I and II to prevent DNA replication leading to apoptosis induction. Elsamitrucin has shown efficacy in leukemia, melanoma, reticulum sarcoma, breast, and colon tumors or xenographs [104]. It has been studied in phase I and multiple phase II trials for multiple cancer types but has only shown patient efficacy in non-Hodgkin’s lymphoma [104,105,106]. ZINC000085633079 had structural similarity to Calanolide A (DB04886) at a score 0.637. Calanolide A is isolated from *Calophyllum lanigerum* and is known for targeting reverse transcription. Other calanolides have shown anticancer effects with MCF-7 cells and HL-60 cells [107]. ZINC000085633079 was structurally similar to Icariin (DB12052) with a score of 0.635. Icariin is a flavonoid obtained from the leaves and stems of *Epimedium* and Icariin and its derivatives are under evaluation for its anticancer effects in numerous cancers through a variety of mechanisms [108].

Several compounds with structural similarity to ZINC000085633079 target heat shock protein 90 (HSP90) including PEN-866 (DB16186), score 0.601, and c-terminal inhibitors including coumermycin A1 (DB13912), score 0.634, troxerutin (DB13124), score 0.626, novobiocin (DB01051), score 0.625, and clorobiocin (DB03966). PEN-866 has finished initial phase I trials exploring safety and efficacy in advanced solid tumors [109]. Both novobiocin and clorobiocin are coumarins. Novobiocin binding to the c-terminal of HSP90 interrupts dimerization and key co-proteins like HER2, Raf-1, and HSP70 [110]. Novobiocin is also a specific DNA polymerase theta inhibitor that can selectively kill cancer cells in homologous recombination-deficient tumors [111]. Clorobiocin has higher efficacy and potency than novobiocin as do other c-terminal binders coumermycin A1, another coumerin, and flavonoid troxerutin [110]. Coumermycin A1 is a scaffold of interest for breast cancer and it was tested in breast cancer cell lines MCF-7 and SkBr3 reporting IC50s of 5μM and 8.8 μM, respectively [112]. Troxerutin is a rutin derivative that displays radioprotective and antioxidant properties and is being explored as an anticancer drug for non-small cell lung cancer, prostate cancer, and hepatocellular carcinoma [113,114,115]. ZINC000085633079 also had structural similarities to other flavonoids monoxerutin (DB13764), score 0.619, hidrosmin (DB13490), score 0.606, diosmin (DB08995), score 0.605, and rutin (DB01698), score 0.603. Rutin disturbs multiple cancer mechanisms in both lung and colorectal cancer cell lines [116]. Diosmin has many biological activities that include anticancer activities in a variety of cancer cell types [117]. ZINC000085633079 also had structural similarity with vancomycin (DB00512), score 0.601, Oritavancin (DB04911), score 0.606, and 4-epi-vancosaminyl derivative of vancomycin (DB04431), score 0.605. Vancomycin in combination with stereotactic body radiation is in phase I clinical trials for treatment of oligoprogressive non-small cell lung cancer [118].

### 2.7. Full-Length ADAR2 Models

#### 2.7.1. Homology Modeling Full-Length ADAR2

**Figure 7:**
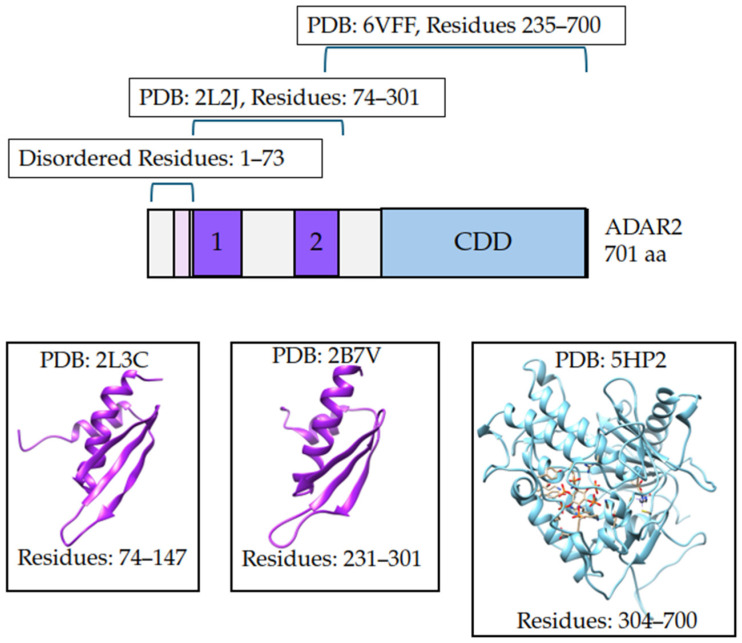
PDB structures of individual ADAR2 domains published to the RCSB PDB.

**Figure 8:**
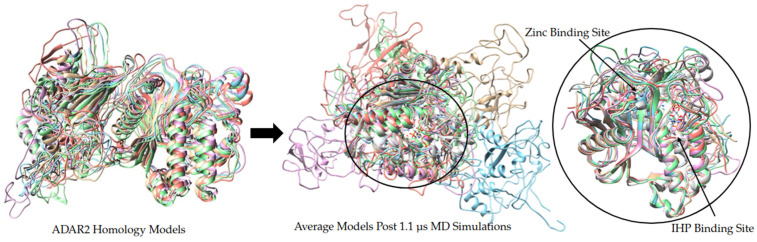
MODELLER generated full-length ADAR2 models prior to MD simulations and then the resulting average models post 1.1 µs MD simulation. The circle is highlighting the CDD and this region is magnified in the circle to the right. Model 1 is tan, model 2 is blue, model 3 is purple, model 4 is green, model 5 is orange, PDB: 5HP2 is in gray.

Homology modeling is a widely utilized computational tool for generating protein models. ADAR2 is a good candidate for homology modeling because a majority of the protein has been solved experimentally in individual domain segments (Figure 7). PDBs 1ZY7, 5HP2, and 6VFF capture the catalytic domain of ADAR2 by X-ray diffraction with resolutions of 1.70 Å, 2.98 Å, and 2.80 Å, respectively [27,41,119]. Notably, PDB: 6VFF also covers dsRBD2 and the connecting region between. Multiple PDBs exist for the two dsRBDs of ADAR2 and all of the dsRBD structures were captured by solution NMR. PDBs used for dsRBD1 were 2L3C and 2B7T and for dsRBD2 were 2L3J and 2B7V [34,35]. The sequence data from these PDBs were used to generate a multiple sequence alignment to the ADAR2, NCBI reference sequence NP_001103.1. MODELLER 10.3 generated five models of the full-length ADAR2 structure (Figure 8). These initial models can be found in the Supplementary. These models were ranked using molpdf, where smaller molpdf values are indicative of models that have fewer violations of spatial constraints. The models were ranked model 5, model 4, model 2, model 3, and then model 1 with molpdf values of 44,881.14844, 46,036.54688, 46,960.29297, 48,832.29297, and 48,846.43359, respectively (Table S18).

#### 2.7.2. Model Quality Assessment and Refinement

ProSA was used to evaluate the overall quality of the predicted ADAR2 models. ProSA compares the energy profiles of a protein in pdb format and compares to experimental structures of comparable size obtained by NMR or X-ray crystallography [120]. The z-scores for the five ADAR2 models fell within the range of z-scores for experimental structures of similar sizes (Figure S6a,c,e,g,i). The generated homology models showed the expected structures for the known domains (Figure S7). The local model quality graphs in ProSA plot the energy value assigned to each residue to the residue index [120]. The majority of residues should have negative energies and areas with positive energies are regions with potential for instability, misfolding, steric clashes, or poorly packed residues. For all five models, residues for the catalytic domain have negative energies; areas with positive energies are in the first two hundred residues (Figure S6b,d,f,h,j). Comparison of the PDB: 5HP2 of the CDD to the average pdb of each ADAR2 model after MD simulation shows highly similar structures supporting the stability of the CDD (Figure 8). In contrast, the dsRBD move around much more during MD simulation, resulting in different average structures (Figure 8).

Quality of the predicted ADAR2 models were then evaluated using SAVESv6.1 for ERRAT score, VERIFY3D score, and Ramachandran plots generated with PROCHECK prior to MD simulation and post 1.1 μs MD simulation for refinement (Table 6 and Table S18). Prior to MD simulation, the ERRAT scores of the five ADAR2 models were low, the highest being model 5 with a score of 63.0592 (Table S18). Following 1.1 μs MD simulation, ERRAT scores were much improved at 82.4348, 81.5972, 78.5211, 79.2096, and 84.0555 for models 1, 2, 3, 4, and 5, respectively (Table 6). The area contributing most to the error of all five models was residues 222–231, a region covering the connecting loop between dsRBD1 and dsRBD2. Additionally, residues 107–111 had error values; this region is the loop before dsRBD1 and beginning the α1 helix of dsRBD1. Model 3 had the largest number or error regions and model 5 had the least amount of error regions.

VERIFY3D analyzes the amino acid sequence with the 3D structure, comparing the structural classifications of residues such as buried or exposed to experimental structures [121]. VERIFY3D is reported as a percentage that represents the fraction of residues passing the 0.2 (3D-1D score) over the total residues [121]. A percentage of 80% or higher is considered a high-quality model as the majority of residues are well placed [120]. The pre-MD simulation only ADAR2 model 2 passed VERIFY3D with a score of 82.45%, although the other models were close ranging from 76.89% to 79,89%, representing acceptable models (Table S18). Following MD simulation, models 1, 3, and 4 passed VERIFY3D with scores of 84.59%, 82.88%, and 80.46%; models 2 and 5 were close at 79.32% and 78.03%, respectively (Table 6).

**Figure 9:**
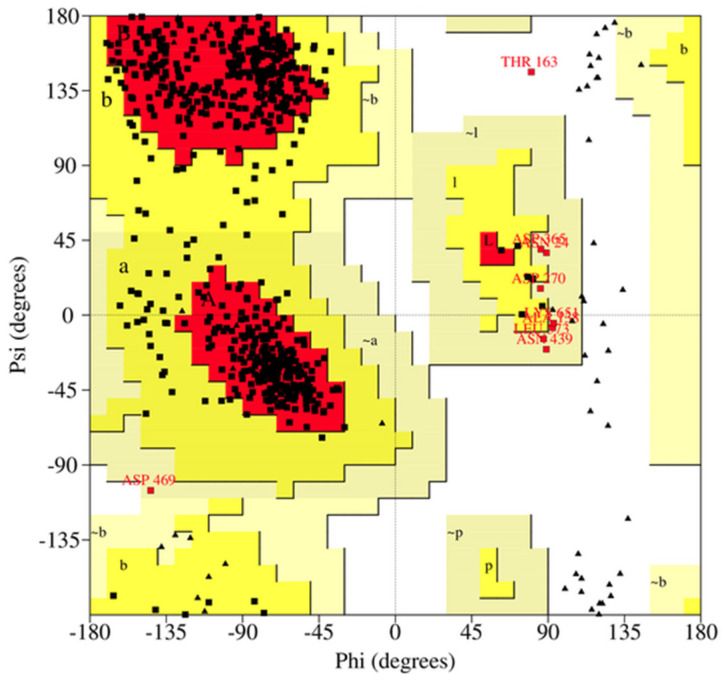
Ramachandran plot generated from PROCHECK following 1.1 μs MD simulation of model 5; plot statistics reported in Table 6. Favored regions (A, B, and L) in red, additional allowed regions (a, b, l, and p) in yellow, generously allowed regions (~a, ~b, ~l, and ~p) in tan, and disallowed regions in white. Squares represent non-glycine residues and triangles represent non-end glycine residues.

Using PROCHECK, Ramachandran plots were generated for each model before and after MD simulation (Figure 9 and Figure S8). Ramachandran plots look at the backbone torsion phi ϕ and psi ψ angles and evaluate the stereochemical quality of the models by comparing the predicted torsion angles to torsion angles common from experimental structures [122]. There was a high majority of residues in the favored region in the ADAR2 models prior to MD simulation with the lowest percentage being model 1 at 84.1% and the highest being model 4 at 86.7% (Table S18). Post-MD simulations, models had favorable percentages of 79.6%, 83.0%, 79.6%, 80.9%, and 84.8% for models 1–5, respectively (Table 6). MD simulation refinement reduced the percentage of residues in the generously allowed and disallowed regions primarily increasing the number of residues in the allowed region (Table 6 and Table S18). Model 5 had the most favorable plot with only one residue in the disallowed region, excluding glycines and prolines, 8 residues in generously allowed regions, 81 residues in allowed regions, and 503 residues in the favorable regions (Figure 9).

#### 2.7.3. Evaluation of Model Stability Post 1.1 μs MD Simulations

Each model underwent 1.1 μs of MD simulation. The models post MD simulation can be found in the Supplementary section. MD simulation is one method for model refinement. Adding dynamics to the protein model can allow the predicted structure to relax into a more energetically favorable state, which can sometimes correct areas with poor geometry, poor side-chain orientations, and aid in refining flexible regions [123].

The models from 800 to 1.1 μs stayed about the same with low deviations observed by alignment of the final structure at point 800 ns to the final structure at 1.1 μs which had RMSD values of 4.834 nm for model 1, 4.500 nm for model 2, 2.633 nm for model 3, 2.858 nm for model 4, and 3.378 nm for model 5. Over the course of 1.1 μs MD simulation, the overall RMSD stayed below 2.5 supporting the stability of the predicted models (Figure S9). During the first 100 ns of simulation, models 1, 4, and 5 had little variation with RMSD values less than 1 nm (Figure S9a). Model 2 had a spike in RMSD at 400 ns and model 1 had a spike in RMSD at 700 ns; each of these spikes jumped from about 1 nm to about 2 nm which is not extreme and may correspond to a conformational change (Figure S9b). During the last 200 ns of simulation all models had high stability with RMSD below 0.8 nm supporting that the models did converge with a highly stable structure (Figure S9c). Model 4 maintained the lowest RMSD values over the course of the 1.1 μs (Figure S9). The Rg of the ADAR2 models was from around 2.8 tp 3.5 nm which is within the expected range for a large modular protein (Figure S10). Model 4 maintained the most compact structure throughout the 1.1 μs simulation and model 3 also stayed relatively compact (Figure S10).

RMSF evaluation supports the intrinsically disordered region within residues 1–75 that maintained high RMSF throughout the 1.1 μs staying around 1–2.5 nm in fluctuation (Figure S11). Model 1 has high RMSF values, greater than 1 nm, in the intrinsically disordered region, residues 2–75, and in the span of the two dsRBDs (74–300) (Figure S11b). This localized flexibility observed by RMSF may correlate to the spike in RMSD observed around 700 ns. Model 2 also spikes in RMSD, but the RMSF changes increase across the protein (Figure S11b). The Rg of model 2 does not increase and instead stays stable during the period of the RMSD spike, suggesting that the increase in flexibility is not due to a change in global unfolding (Figure S10b). During the final 200 ns of MD simulation, the intrinsically disordered region and dsRBDs continue to maintain flexibility; however, the catalytic domain region appears to stay more stable with RMSF values below 0.5 nm (Figure S11c).

## 3. Materials and Methods

This study followed a computer-aided drug discovery approach to shortlist compounds predicted to bind the interface of dsRBD2 of ADAR2 with RNA. Molecular docking of the TCM database to the dsRBD2 was used to rank natural compounds by docking score. The top 1% of compounds from molecular docking then underwent an evaluation for predicted safety and drug-likeness. Lead compounds were found to bind with high affinity to the dsRBD2 via molecular mechanics Poisson–Boltzmann surface area (MM/PBSA) calculations were conducted post 100 nanosecond (ns) molecular dynamics (MD) simulations. The binding of top compounds was then further assessed by noting key protein–ligand interactions and per residue energy contributions. Of compounds predicted to have high affinity during protein–ligand MD simulations, these underwent further MD simulation in the presence of RNA substrate. Lead compounds retain negative delta-free energy in the presence of RNA substrate. These lead scaffolds were assessed for predicted biological activity and structural similarities. In addition, this paper describes five ADAR2 models generated by homology modeling.

### 3.1. Receptor and RNA Substrate Preparation

Multiple structures of ADAR dsRBD were made publicly available on the Research Collaboratory for Structural Bioinformatics Protein Data Bank (RCSB PDB) [124]⁠. Structures for dsRBD2 of ADAR2 included the following PDBss: 2B7V, 2L3J, and 6VFF [34,35,41]⁠. The PDB: 2B7V was captured by solution NMR with 50 calculated conformers and 20 submitted conformers. PDB: 2B7V covered 71 residues from 231 to 301 that covers the 2dsRBD of ADAR2 [34]. The structure of 2L3J was captured by solution NMR with 40 conformers calculated, and 19 submitted conformers. PDB: 2L3J covered the span of ADAR2 residues 74–301 and included both the first dsRBD and second dsRBD of ADAR2 in a complex with a dsRNA substrate [35]⁠. The PDB: 6VFF was captured by X-ray diffraction at a resolution of 2.80 Å and chain B covered the most portion of the dsRBD2 going from ADAR2 residues: 235–463, 476–495, and 512–700 [41]. Additionally, chain A of PDB: 6VFF spanned ADAR2 residues: 319–700 [41]. The PDB: 2L3J covered the largest portion of the dsRBD2 and was captured in a complex with the RNA substrate. The RNA substrate captured in this structure was of the pre-mRNA of the α-hydroxyl-5-methyl-4-isoxazole-propionate (AMPA) glutamate receptor 2 (GluR-2). Specifically, the 2L3J structure uses GluR-2 pre-mRNA which is a 71 nt dsRNA stem loop of the transcript holding the R/G site. The GluR-2 R/G site is a known ADAR2 editing site that influences desensitization of the AMPA receptor but not calcium permeability [125]⁠. Overall, the 2L3J structure was utilized for extraction of residues 158–228, corresponding to ADAR2 residues 231–301, containing the second dsRBD of ADAR2, as well as the GluR-2 dsRNA. Using the GROningen Machine for Chemical Simulations (GROMACS) v 5.1.5, a 100 ns molecular dynamics simulation was performed on the second dsRBD protein structure using the OPLS/AA forcefield [126]⁠. The average pdb structure following the 100 ns simulation was then used for molecular docking after being converted to the acceptable protein data bank, partial charge, and atom type (.pdbqt) formats using the make macromolecule function provided by PyRx 0.9.2 [127]⁠.

### 3.2. Screening Library Preparation

No prior compounds have been reported to bind the interface of dsRBD2-RNA. A library of 35,161 natural compounds was collected from the ZINC15 database Traditional Chinese Medicine (TCM) [128,129]⁠. The TCM compounds were filtered for molecular weights between 150 and 600 g/mol as performed previously, leaving 25,212 ligands [130]⁠. These ligands were energy minimized using the Universal Force Field (UFF) [131]⁠. The minimized ligands were then converted to pdbqt format via PyRx 0.9.2

### 3.3. Molecular Docking to the Protein–RNA Interface

The RNA interface between the RNA and dsRBD2 structure had been previously validated by mutagenesis experiments to confirm the importance of specific residues in the contact interface [35]⁠. Key residues within this interface included Val237, Met238, Asn241, Glu242, Ser258, His259, Phe263, Lys281, Lys282, and Lys285. Mutation of Met238 could drop RNA editing by around 30%, mutation of Ser258 and His259 could almost abolish editing, and mutation of the lysine residues could completely block RNA binding and editing of specific transcripts dependent on the second dsRBD [34,35]⁠. A grid box dimension of 9.12982238375 × 24.5221426245 × 12.8583348177 was created, centered at x = 70.6080305435 Å, y = 13.3315499285 Å, z = 64.6079256357 Å, containing all of the identified interface residues. For each docking run, the exhaustiveness was limited to 8. Compounds with top docking scores were visualized with PyMol to verify the location of the ligand to the protein–RNA interface.

### 3.4. ADMET Prediction

Compounds predicted to have good binding affinities to the dsRBD2-RNA interface were then shortlisted for ADMET properties by screening with SwissADME [132]⁠ and OSIRIS Data Warrior v5.5.0 [133]. The selected compounds were evaluated based on Veber’s Rule and Lipinski’s rule of five, which assess their drug-likeness and pharmacokinetic properties. Compounds must have no violations to Veber’s rule and no more than one violation to Lipinski’s rule of five. To further refine the selection, toxicity risks such as mutagenicity, tumorigenicity, irritancy, and potential effects on reproduction were analyzed. Compounds displaying low or high risks for mutagenicity, tumorigenicity, or irritancy were removed from consideration. This comprehensive analysis aimed to enhance the understanding of the pharmacokinetic characteristics and safety profiles of the compounds, providing critical insights into their viability as therapeutic candidates. Additionally, these evaluations were instrumental in identifying and excluding compounds that posed significant toxicity risks or exhibited poor safety profiles, thereby streamlining the selection process and ensuring a focus on candidates with a higher likelihood of clinical success. From leads that passed ADMET prediction, 6 of 14 had an identical docking score. Among them, three were excluded due to high reproductive toxicity. Reproductive effects were only used to filter compounds with identical docking scores. Compounds with stronger binding affinities than the −7.5 kcal/mol were retained to support SAR analysis.

### 3.5. Molecular Dynamics Simulations

Compounds passing ADMET screening were then subjected to 100 ns protein–ligand MD simulations via GROMACs version 5.1.5 with dt of 0.002 (2 fs) and the saved conformations every 10 ps [126]⁠. For preparation of the protein–ligand complexes, each of the selected ligands had all hydrogen atoms added and their charges ascertained by UCSF Chimera. These ligands were then submitted to ANTECHAMBER for generation of GROMACS topology file and AMBER forcefield parameter file [134]⁠. The protein, dsRBD2, and protein–ligand complexes were centered in boxes with 1 nm spacial edges. Compounds determined to have high binding affinities during the protein–ligand complexes were then assessed for their binding affinities during protein–ligand–RNA MD simulations. The protein–RNA, dsRBD2–GluR-2, and protein–ligand–RNA complexes were centered in boxes with 1.5 nm edges. All simulations were prepared with the TIP3P water model compatible with the AMBER forcefield [135,136]⁠. An intracellular sodium concentration of 12 mM was obtained and systems neutralized by the addition of sodium or chlorine atoms. The systems underwent energy-minimization followed by NVT and NPT equilibration steps, for controlling constants of number of particles (N), volume (V), temperature (T), or pressure (P), prior to running the 100 ns MD simulations. These simulations were conducted to investigate and compare the dynamic behavior, structural integrity, and intermolecular interaction patterns of the dsRBD2 under three distinct conditions: the unbound state, when complexed with the shortlisted ligands, and when complexed with RNA substrate and the lead compounds. The root mean squared deviation (RMSD), root mean squared fluctuation (RMSF), radius of gyration (Rg), and hydrogen bonding was analyzed. These analyses aimed to uncover differences in the conformational flexibility and stability of the proteins upon ligand binding, shedding light on how ligand interaction influences the protein’s structural dynamics.

### 3.6. MM/PBSA Calculations

MM/PBSA calculations were implemented using the g_mmpbsa tool [137]⁠. These calculations were employed to perform energy decomposition analyses and provide detailed insights into the energetic contributions within the studied complexes. This approach allowed for the evaluation of various energy components critical to protein–ligand interactions. Specifically, the analyzed energy terms included van der Waals (vdW), electrostatic interactions, polar solvation energy, and solvent-accessible surface area (SASA)-based contributions, along with total calculation of estimated binding free energy. These calculations offered a quantitative understanding of the energetic factors stabilizing the complexes [137]⁠. Compounds reporting negative free energies from MM/PBSA calculations from the protein–ligand complexes were then further evaluated in protein–ligand–RNA MD simulations. Additionally, the per-residue energy contributions for each system were calculated, identifying critical residues that make significant contributions to ligand binding and play a dominant role in ligand stabilization.

### 3.7. Technical Replicates

The same input structure can generate slightly variable results. The variation in result when evaluating the binding energy can be important when the binding energy is close to zero. Technical replicates were included for top compounds to assess the consistency of the results. The top compounds were predicted to bind dsRBD2 during both protein–ligand MD simulation and during protein–ligand–RNA MD simulation. The reproducibility of the four lead compounds, and ZINC000003203078, predicted not to bind the dsRBD22, was tested by running three technical replicates for each MD run. The technical replicates were the same initial input structures, same forcefield, and same parameters. Each MD simulation includes a velocity generation where a random seed is used to generate the initial velocity, and altering the initial velocity can influence the final simulation trajectory. The average binding energies from MM/PBSA calculations, RMSD, and Rg were evaluated by taking the average results from the three replicate simulations.

### 3.8. Prediction of Biological Activity for Lead Compounds

Lead compounds were then evaluated for potential biological activities. This process included a structural similarity search via DrugBank [138]⁠ and a Prediction of Biological Activity Spectra of Substances (PASS) search via Way2Drug [139]⁠. The structural similarity search aimed to use chemical structure knowledge of comparable compounds meeting a threshold of greater than 60% similarity to identify potential biological activities related to cancer therapeutics. The PASS search similarly uses a computational algorithm to define structure–activity relationships and statistically predict the likelihood of a biological activity. Both methods focus on the premise that a compound’s biological activity is linked to its molecular structure [140]. The relevance of biological activities predicted guided which compounds should be prioritized for further study.

### 3.9. Protein–Ligand Interactions

Using LigPlot+ [141], protein–ligand interaction plots were generated for analysis focused on identifying key non-covalent interactions, such as hydrogen bonds and hydrophobic contacts that stabilize the protein–ligand complexes. Hydrogen bonds during the MD simulations were also assessed using gmx hbond. These visualizations in addition to the per-residue energy contributions provide data for the prediction of chemical modifications that could be made to lead compounds to aid in binding and stability.

### 3.10. Generating Full-Length ADAR2 Models

#### 3.10.1. Homology Modeling

Homology modeling was used to predict the full-length models of ADAR2. Individual protein domains of ADAR2 have been captured by NMR spectrometry and X-ray crystallography. Currently, no full-length ADAR2 models have been published to the RCSB PDB. Primary sequence data for ADAR2 isoform 701 aa were collected from NCBI, reference sequence NP_001103.1 [142]⁠. For templates, structures from ADAR2 individual domains published to the RCSB PDB were assessed for resolution and number of missing residues. Ultimately, PBBs used for comparative modeling of the ADAR2 full-length structure included 2L3C and 2L3J [35]⁠, 2B7V [34]⁠, 6VFF [41]⁠, 1ZY7 [27], and 5HP2 [119]⁠. The sequence data for these PDB structures were then used to generate a multiple sequence alignment using COBALT in MODELLER. Due to repetitive sequences, the alignments of the templates had to be manually fixed in the query-mult.ali files for use in AutoModel within MODELLER. MODELLER 10.3 was used to generate five models of full-length ADAR2 protein using multi-template modeling. Molecular probability density function (molpdf) score was used to rank the comparative protein models.

#### 3.10.2. Simulation-Based Model Refinement

The five predicted ADAR2 models then underwent energy minimization to resolve steric clashes and improve bond geometry. Each model underwent a total of 1.1 µs MD simulation using GROMACS 2023.3. The OPLS/AA forcefield previously resulted in the lowest potential energies and smallest RMSDs, and the CHARMM36 forcefield held comparable results [143]. The OPLS/AA forcefield was used for the initial 100 ns MD simulation followed by an additional 800 ns simulation using the CHARMM36 forcefield and the TIP3P water model. Following the net 900 ns simulation, the output protein model was run for an additional 200 ns using the OPLS forcefield and TIP4P water model. The outputs from the 900 ns and 1.1 µs end points were compared to support that use of the CHARMM36 forcefield did not greatly differ from the end result of the OPLS run simulations. Ultimately, the end protein models from the net 1.1 µs MD simulation were used for evaluating model quality.

#### 3.10.3. Model Quality Assessment

Validation tools to assess model quality and reliability of the predicted models included Protein Structure Analysis program (ProSA) [120]⁠ and Structure Analysis and Verification Server v6.1 (SAVEs). SAVES contains multiple tools for the assessment of quality based on information from known structures’ geometry and stereochemistry. Model quality was assessed by Verify3D score [121]⁠ and ERRAT [144] score. In addition, analysis of Ramachandran plots created by PROCHECK were used to compare changes in the favorable, additional allowed, generously allowed, and disallowed regions between the initial models and the refined models [122]⁠. These quality assessments aimed to identify regions of concern for steric clashes, erroneous bond geometry, and outlier residues.

## 4. Conclusions

Eight ligands, ZINC000085597263, ZINC000085633079, ZINC000014649947, ZINC000034512861, ZINC000070454124, ZINC000085594944, ZINC000085633008, and ZINC000095909822, were successfully docked to the RNA binding interface of ADAR2 dsRBD2, had acceptable drug-likeness and predicted safety, and were calculated to have high binding affinity with dsRBD2 from MM/PBSA (Figure S12). The protein–ligand interactions of these eight complexes were assessed to identify key residues and interactions contributing to their binding affinities. Additional dsRBD–ligand–RNA MD simulations were carried out for the eight identified ligands. Only four compounds, ZINC000085597263, ZINC000085633079, ZINC000014649947, and ZINC000034512861, were calculated to have negative binding affinity to the dsRBD2 in the presence of RNA substrate GluR-2. Residues Val164, Met165, Lys209, Lys212 were key residues in ligand binding even in the presence of RNA substrate. Ligand binding to these key recognition and interaction residues is predicted to inhibit dsRBD2 from binding RNA. The predicted biological activities of the compounds ZINC000014649947, ZINC000034512861, ZINC000085597263, and ZINC000085633079 for anticancer activities related to inflammation, DNA damage, DNA replication, cell proliferation, and metastasis support their potential as anticancer candidates for the treatment of mesothelioma. Compounds ZINC000014649947, ZINC000034512861, ZINC000085597263, and ZINC000085633079 have identified structurally similar compounds with supported anti-mesothelioma activities or related anticancer activities. While these compounds have potential for binding to the dsRBD2, their true activity in mesothelioma cell lines should be further evaluated. These scaffolds may have valuable therapeutic potential worthy of further exploration in vitro. Further study of these compounds in vitro should focus on validation of ligand-binding affinities to the dsRBD2, inhibition of RNA binding by ADAR2, and whether these compounds can significantly decrease mesothelioma cell proliferation, motility, and invasiveness. Current therapeutics for mesothelioma are poor. Optimization of these compounds may provide a new line of therapeutics for mesothelioma or the use of these compounds in combination with existing treatments like cisplatin–pemetrexed may increase current treatment efficacy. Additionally, five full-length ADAR2 models with good quality are reported here. These models can be used for gaining insights into how ADAR2 functions may be impacted by mutations, areas for protein engineering to increase ADAR2 stability or RNA binding specificity, and insights into protein interactions like how dimerization may alter global protein structure or changes in protein structure upon complex formation with other proteins or RNAs of varying structures.

## Figures and Tables

**Table 1:** Summary of key physicochemical properties of selected compounds.

Compound	MW (g/mol)	logP o/w	TPSA (Å^2^)	BBB Permeant	GI Absorption	ESOL Solubility Class	Lipinski’s Rule of Five	Veber’s Rule
ZINC000085594944	566.62	3.89	103.65	No	High	Moderately	1	0
ZINC000085633079	556.56	5.46	102.64	No	Low	Poor	1	0
ZINC000003203078	376.36	3.58	67.13	Yes	High	Moderately	0	0
ZINC000034512861	468.75	7.64	26.3	No	Low	Poor	1	0
ZINC000085594687	488.57	4.75	104.65	No	High	Poor	0	0
ZINC000095909822	446.66	4.59	74.6	No	High	Moderately	0	0
ZINC000085571242	488.66	4.59	52.6	No	High	Moderately	0	0
ZINC000085597263	594.73	5.84	114.68	No	Low	Poor	1	0
ZINC000044305204	582.64	5.05	136.68	No	Low	Poor	1	0
ZINC000014649947	426.5	5.55	69.92	No	High	Poor	1	0
ZINC000059589174	486.52	2.96	82.39	No	High	Moderately	0	0
ZINC000102943567	404.46	4.29	72.83	No	High	Moderately	0	0
ZINC000085633008	494.53	5.3	100.13	No	Low	Poor	0	0
ZINC000014690589	488.57	5.78	100.13	No	Low	Poor	0	0
ZINC000042807177	494.62	4.15	89.9	No	High	Moderately	0	0
ZINC000085545309	374.6	7.04	0	No	Low	Poor	1	0
ZINC000085504706	550.86	7.04	17.07	No	Low	Poor	1	0
ZINC000085571230	526.69	7.04	64.96	No	Low	Poor	1	0
ZINC000085532515	533.7	5.05	68.23	No	High	Poor	1	0
ZINC000070454124	564.58	3.83	119.34	No	High	Moderately	1	0

Molecular weight (MW), consensus logP, topological polar surface area (TPSA), blood–brain barrier (BBB) permeance, gastrointestinal (GI) absorption, and estimated solubility ESOL class.

**Table 2:** Representation table for top 14 compounds ranked by docking score following ADMET screening.

Compound	Docking Score	Mutagenicity	Tumorigenicity	Irritant	Reproductive Effects
ZINC000085594944	−8.0	none	none	none	high
ZINC000085633079	−7.9	none	none	none	high
ZINC000003203078	−7.8	none	none	none	high
ZINC000034512861	−7.8	none	none	none	none
ZINC000085597263	−7.7	none	none	none	none
ZINC000095909822	−7.7	none	none	none	none
ZINC000014649947	−7.6	none	none	none	none
ZINC000044305204	−7.6	none	none	none	none
ZINC000070454124	−7.5	none	none	none	none
ZINC000085532515	−7.5	none	none	none	none
ZINC000085633008	−7.5	none	none	none	none
ZINC000014690589	−7.5	none	none	none	high
ZINC000059589174	−7.5	none	none	none	high
ZINC000085504706	−7.5	none	none	none	high

Toxicity risks are classified by “none”, “low”, and “high” for the categories of mutagenicity, tumorigenicity, irritant, and reproductive effects.

**Table 3:** Energy components contributing to protein–ligand interactions as determined by MM/PBSA calculations.

Compound	Van der Waals	Electrostatic Energy	Polar Solvation Energy	SASA Energy	Binding Energy
ZINC000003203078	−71.024 ± 2.193	58.918 ± 7.742	53.296 ± 4.731	−8.056 ± 0.244	33.283 ± 4.599
ZINC000014649947	−126.183 ± 1.619	−39.692 ± 1.800	88.947 ± 2.380	−14.967 ± 0.160	−91.971 ± 2.264
ZINC000034512861	−129.574 ± 1.785	−1.690 ± 0.843	28.921 ± 2.291	−14.823 ± 0.188	−117.174 ± 1.991
ZINC000044305204	2515.289 ± 8.456	−31.312 ± 1.848	163.827 ± 3.099	−19.991 ± 0.145	2647.642 ± 8.562
ZINC000070454124	−131.220 ± 2.212	−57.121 ± 2.408	113.517 ± 3.493	−14.962 ± 0.213	−89.821 ± 2.037
ZINC000085532515	−85.434 ± 3.705	174.586 ± 6.171	5.388 ± 4.444	−10.714 ± 0.469	83.573 ± 3.013
ZINC000085594944	−121.825 ± 1.886	−33.966 ± 1.737	73.172 ± 3.440	−14.313 ± 0.196	−96.990 ± 3.440
ZINC000085597263	−145.826 ± 3.688	−40.985 ± 3.693	87.528 ± 4.872	−16.874 ± 0.358	−115.873 ± 3.902
ZINC000085633008	−110.509 ± 2.538	−17.199 ± 1.943	58.462 ± 2.771	−12.723 ± 0.280	−82.006 ± 2.708
ZINC000085633079	−176.138 ± 1.693	−55.252 ± 1.604	112.556 ± 2.150	−17.904 ± 0.128	−136.667 ± 1.779
ZINC000095909822	−95.872 ± 2.116	−27.994 ± 2.310	70.238 ± 3.996	−12.336 ± 0.228	−66.133 ± 2.909

Each energy value is in kJ/mol.

**Table 4:** Energy components contributing to protein–RNA interactions and RNA–protein–ligand interactions as determined by MM/PBSA calculations.

Compound	Van der Waals	Electrostatic Energy	Polar Solvation Energy	SASA Energy	Binding Energy
dsRBD2-GluR-2	−383.563 ± 0.493	−8050.498 ± 4.241	1463.402 ± 2.193	−40.719 ± 0.042	−7011.460 ± 3.184
ZINC000003203078	−123.797 ± 1.756	1377.269 ± 8.971	137.191 ± 5.025	−12.923 ± 0.187	1377.422 ± 7.839
ZINC000014649947	−155.167 ± 1.519	−56.383 ± 2.260	180.212 ± 3.513	−17.598 ± 0.185	−48.847 ± 2.416
ZINC000034512861	−34.158 ± 4.893	−2.938 ± 0.881	38.383 ± 9.318	−4.058 ± 0.560	−3.185 ± 9.674
ZINC000070454124	4877.875 ± 11.057	−81.655 ± 1.459	264.516 ± 2.752	−26.988 ± 0.178	5034.415 ± 11.006
ZINC000085594944	1889.835 ± 7.258	−67.286 ± 2.597	213.467 ± 4.104	−24.662 ± 0.253	2011.413 ± 7.829
ZINC000085597263	−230.554 ± 2.862	−35.540 ± 3.200	109.973 ± 3.586	−21.324 ± 0.211	−177.129 ± 3.290
ZINC000085633008	1822.482 ± 6632	−17.916 ± 1.836	153.393 ± 2.634	−21.525 ± 0.118	1936.952 ± 7.130
ZINC000085633079	−253.380 ± 3.058	−32.051 ± 3.911	161.361 ± 3.202	−24.988 ± 0.168	−148.844 ± 3.463
ZINC000095909822	1491.132 ± 6.301	−31.553 ± 1.759	109.608 ± 3.027	−16.087 ± 3.027	1552.963 ± 6.200

Each energy values are in kJ/mol.

**Table 5:** Protein–ligand interactions plots of eight dsRBD2–ligand complexes.

Compound	Hydrophobic Bonds	Hydrogen Bonds (Bond Length Å)
ZINC000014649947	Ala213, Lys208, and Lys209	Asn162 (2.35)
ZINC000034512861	Asn162, Val164, Lys208, Lys209, and Lys212	
ZINC000085633079	Val164, Met165, Glu169, Lys208, Lys209, and Lys212	Asn162 (2.71), Asn168 (2.66)
ZINC000085597263	Val164, Met165, Lys208, Lys209, and Lys212	Asn162 (3.14), Asn168 (2.00)
ZINC000085594944	Asn162, Val164, Met165, Asn168, Lys208, Lys209, and Lys212	
ZINC000085633008	Asn162, Val164, Met165, Asn168, Asn207, Lys208, Lys209, Leu210, and Ala213	
ZINC000070454124	Val164, Asn168, Pro172, Lys209, Leu210, and Lys212	Asn162 (2.81)
ZINC000095909822	Val164, Met165, Asn168, Lys208, Lys209, and Lys212	

**Table 6:** Summary table of results for ERRAT, VERIFY3D, and PROCHECK from SAVESv6.1for models 1–5 of ADAR2 post 1.1 μs MD simulation.

Post 1.1 µs MD Simulations ADAR2						
Model	ERRAT	VERIFY3D	PROCHECK			
			Favored	Allowed	Generously Allowed	Disallowed
Model 1	82.4348	84.59%	79.6%	18.9%	1.3%	0.2%
Model 2	81.5972	79.32%	83.0%	15.2%	1.5%	0.3%
Model 3	78.5211	82.88%	79.6%	18.7%	1.2%	0.5%
Model 4	79.2096	80.46%	80.9%	16.9%	1.7%	0.5
Model 5	84.0555	78.03%	84.8%	13.7%	1.3%	0.2%

## Data Availability

The original contributions presented in this study are included in the article. Further inquiries can be directed to the corresponding author, wmiller6@luc.edu, and https://github.com/WAMillerLab/ADAR/tree/ADAR2. Accessed on 21 April 2025.

## Supplementary Materials

The following supporting information can be downloaded at: https://www.mdpi.com/article/10.3390/ijms26094075/s1. ADAR2 protein structure files (PDB format) can be downloaded at: https://github.com/WAMillerLab/ADAR/tree/ADAR2. Accessed on 21 April 2025.

## Abbreviations

A-I	Adenosine to Inosine
ADAR	Adenosine deaminases acting on RNA
GBM	Glioblastoma multiforme
dsRBD	Double-stranded RNA binding domain
RBP	RNA binding proteins
NMR	Nuclear magnetic resonance
MM/PBSA	Molecular mechanics Poisson–Boltzmann surface area
MD	Molecular dynamics
AMPA	α-hydroxyl-5-methyl-4-isoxazole-propionate
GluR-2	Glutamate receptor 2
GROMACS	GROningen Machine for Chemical Simulations
TCM	Traditional Chinese Medicine
UFF	Universal Force Field
ADMET	Absorption, Distribution, Metabolism, Excretion, and Toxicity
RMSD	Root mean squared deviation
RMSF	Root mean squared fluctuation
Rg	Radius of gyration
vdW	van der Waals
SASA	Solvent-accessible surface area
PASS	Prediction of biological activity spectra of substances
RCSB PDB	Research collaboratory for structural bioinformatics protein data bank
Molpdf	Molecular probability density function
ProSA	Protein structure analysis program
SAVES	Structure analysis and verification server
SAR	Structure activity relationships
ROS	Reactive oxygen species
RNS	Reactive nitrogen species
GSH	Glutathione
GST	Glutathione S-transferase
GPX-1	Glutathione peroxidase-1
