# Supplementary material for: Computer-Aided Discovery of Natural Compounds Targeting the ADAR2 dsRBD2-RNA Interface and Computational Modeling of Full-Length ADAR2 Protein Structure
Source: Int J Mol Sci. 2025 Apr 25;26(9):4075. doi: 10.3390/ijms26094075 (PMC12072074; doi:10.3390/ijms26094075)
Supplement: Supplementary file 1 [file ijms-26-04075-s001.zip › Supplementary_Final.pdf]

Supplementary Table 1. Docking scores of top 25 compounds from molecular docking to the 2dsRBD-GluR-2 interface. Each docking score is reported in kcal/mol.

Compound	Docking Score
ZINC000085569217	-8.1
ZINC000070450887	-8
ZINC000085594944	-8
ZINC000085633079	-7.9
ZINC000003203078	-7.8
ZINC000034512861	-7.8
ZINC000085594687	-7.7
ZINC000085597263	-7.7
ZINC000095909822	-7.7
ZINC000085571242	-7.7
ZINC000044305204	-7.6
ZINC000095910044	-7.6
ZINC000014649947	-7.6
ZINC000095909571	-7.5
ZINC000059589174	-7.5
ZINC000085545309	-7.5
ZINC000085504706	-7.5
ZINC000085571230	-7.5
ZINC000085532515	-7.5
ZINC000102943567	-7.5
ZINC000070454124	-7.5
ZINC000085633008	-7.5
ZINC000014690589	-7.5
ZINC000042807177	-7.5

Supplementary Table 2. Toxicity prediction results for 138 compounds. Compounds are listed in order by their docking score (kcal/mol). Compounds with no predicted toxicities are highlighted in green. Compounds that failed the screen are highlighted in red.

Compound	Docking Score	Mutagenic	Tumorigenic	Reproductive Effects	Irritant
ZINC000085594944	-8	none	none	high	none
ZINC000085633079	-7.9	none	none	high	none
ZINC000003203078	-7.8	none	none	high	none
ZINC000034512861	-7.8	none	none	none	none
ZINC000085597263	-7.7	none	none	none	none
ZINC000095909822	-7.7	none	none	none	none
ZINC000085571242	-7.7	none	none	none	high
ZINC000085594687	-7.7	none	none	none	high
ZINC000014649947	-7.6	none	none	none	none
ZINC000044305204	-7.6	none	none	none	none

ZINC000014690589	-7.5	none	none	high	none
ZINC000059589174	-7.5	none	none	high	none
ZINC000085504706	-7.5	none	none	high	none
ZINC000070454124	-7.5	none	none	none	none
ZINC000085532515	-7.5	none	none	none	none
ZINC000085633008	-7.5	none	none	none	none
ZINC000102943567	-7.5	none	low	none	none
ZINC000042807177	-7.5	none	none	none	high
ZINC000085571230	-7.5	none	none	none	high
ZINC000085545309	-7.5	none	high	none	high
ZINC000095911783	-7.4	none	none	high	none
ZINC000085594066	-7.4	low	low	high	high
ZINC000014692098	-7.4	none	none	low	none
ZINC000085547091	-7.4	none	none	low	none
ZINC000070451259	-7.4	none	none	none	none
ZINC000085571535	-7.4	none	none	none	high
ZINC000095909662	-7.4	none	none	none	high
ZINC000015148357	-7.3	none	none	high	none
ZINC000014558326	-7.3	none	none	low	none
ZINC000085571488	-7.3	none	high	low	high
ZINC000003978348	-7.3	none	none	none	none
ZINC000014768158	-7.3	none	none	none	none
ZINC000070455627	-7.3	none	none	none	none
ZINC000085531496	-7.3	none	none	none	none
ZINC000085592968	-7.3	none	none	none	none
ZINC000070454205	-7.3	low	low	none	low
ZINC000095908883	-7.3	high	none	none	high
ZINC000095913533	-7.3	high	high	none	high
ZINC000014712269	-7.2	none	none	high	none
ZINC000014825188	-7.2	none	none	high	none
ZINC000095919373	-7.2	none	none	high	none
ZINC000004097766	-7.2	high	none	high	none
ZINC000070454678	-7.2	high	none	high	none
ZINC000070454986	-7.2	high	high	high	high
ZINC000014825201	-7.2	none	none	low	none
ZINC000085547151	-7.2	none	none	low	none
ZINC000085571500	-7.2	none	high	low	high
ZINC000004722964	-7.2	none	none	none	none
ZINC000013375648	-7.2	none	none	none	none
ZINC000014679177	-7.2	none	none	none	none
ZINC000033833348	-7.2	none	none	none	none
ZINC000033834133	-7.2	none	none	none	none
ZINC000085543571	-7.2	none	none	none	none
ZINC000085568150	-7.2	none	none	none	none

ZINC000085597267	-7.2	none	none	none	none
ZINC000095915077	-7.2	none	none	none	none
ZINC000103559648	-7.2	none	none	none	none
ZINC000085569450	-7.2	high	none	none	none
ZINC000095910492	-7.2	low	none	none	high
ZINC000006050189	-7.1	none	none	high	none
ZINC000085506245	-7.1	none	none	high	none
ZINC000095912674	-7.1	none	none	high	none
ZINC000005836649	-7.1	high	none	high	none
ZINC000085569194	-7.1	none	high	high	none
ZINC000085596478	-7.1	none	high	high	none
ZINC000085594057	-7.1	none	low	high	high
ZINC000085594065	-7.1	low	low	high	high
ZINC000095919343	-7.1	none	none	low	high
ZINC000003815415	-7.1	none	none	none	none
ZINC000004027981	-7.1	none	none	none	none
ZINC000005518250	-7.1	none	none	none	none
ZINC000014594730	-7.1	none	none	none	none
ZINC000014768164	-7.1	none	none	none	none
ZINC000014819255	-7.1	none	none	none	none
ZINC000033829730	-7.1	none	none	none	none
ZINC000038894226	-7.1	none	none	none	none
ZINC000070454501	-7.1	none	none	none	none
ZINC000070454886	-7.1	none	none	none	none
ZINC000085532524	-7.1	none	none	none	none
ZINC000085543530	-7.1	none	none	none	none
ZINC000085543640	-7.1	none	none	none	none
ZINC000085592360	-7.1	none	none	none	none
ZINC000085592957	-7.1	none	none	none	none
ZINC000085631264	-7.1	none	none	none	none
ZINC000085642854	-7.1	none	none	none	none
ZINC000095619883	-7.1	none	none	none	none
ZINC000095909490	-7.1	none	none	none	none
ZINC000095911782	-7.1	none	none	none	none
ZINC000095914466	-7.1	none	none	none	none
ZINC000095914537	-7.1	none	none	none	none
ZINC000085597473	-7.1	low	none	none	none
ZINC000004097695	-7.1	high	none	none	low
ZINC000070454041	-7.1	none	none	none	high
ZINC000085490795	-7.1	none	none	none	high
ZINC000085571553	-7.1	none	none	none	high
ZINC000085571573	-7.1	none	none	none	high
ZINC000095918818	-7.1	none	none	none	high
ZINC000103526876	-7.1	none	none	none	high

ZINC000004098749	-7	none	none	high	none
ZINC000013299300	-7	none	none	high	none
ZINC000014594241	-7	none	none	high	none
ZINC000014637370	-7	none	none	high	none
ZINC000014883690	-7	none	none	high	none
ZINC000059735698	-7	none	none	high	none
ZINC000085593564	-7	none	none	high	none
ZINC000095910037	-7	none	none	high	none
ZINC000095912799	-7	none	none	high	none
ZINC000085569197	-7	none	high	high	none
ZINC000085569198	-7	none	high	high	none
ZINC000014585792	-7	none	none	low	none
ZINC000014727603	-7	none	none	low	none
ZINC000085547185	-7	none	none	low	none
ZINC000014558323	-7	low	low	low	none
ZINC000004098004	-7	none	none	none	none
ZINC000015147753	-7	none	none	none	none
ZINC000015205964	-7	none	none	none	none
ZINC000038733772	-7	none	none	none	none
ZINC000059586604	-7	none	none	none	none
ZINC000059774507	-7	none	none	none	none
ZINC000085546354	-7	none	none	none	none
ZINC000085626999	-7	none	none	none	none
ZINC000085631294	-7	none	none	none	none
ZINC000095911347	-7	none	none	none	none
ZINC000095912453	-7	none	none	none	none
ZINC000095912875	-7	none	none	none	none
ZINC000095918872	-7	none	none	none	none
ZINC000102943573	-7	none	none	none	none
ZINC000013378521	-7	low	low	none	none
ZINC000000900926	-7	high	high	none	none
ZINC000059585735	-7	none	none	none	high
ZINC000085571198	-7	none	none	none	high
ZINC000085571239	-7	none	none	none	high
ZINC000085571585	-7	none	none	none	high
ZINC000085594631	-7	none	none	none	high
ZINC000085921663	-7	none	none	none	high
ZINC000014966151	-7	low	none	none	high
ZINC000100901395	-7	none	high	none	high
ZINC000095913553	-7	high	high	none	high

Supplementary Table 3. Summary table of toxicity prediction results.

	Mutagenicity	Tumorigenicity	Irritant	Reproductive Effects

None	120	119	104	92
Low	8	7	2	12
High	10	12	32	34

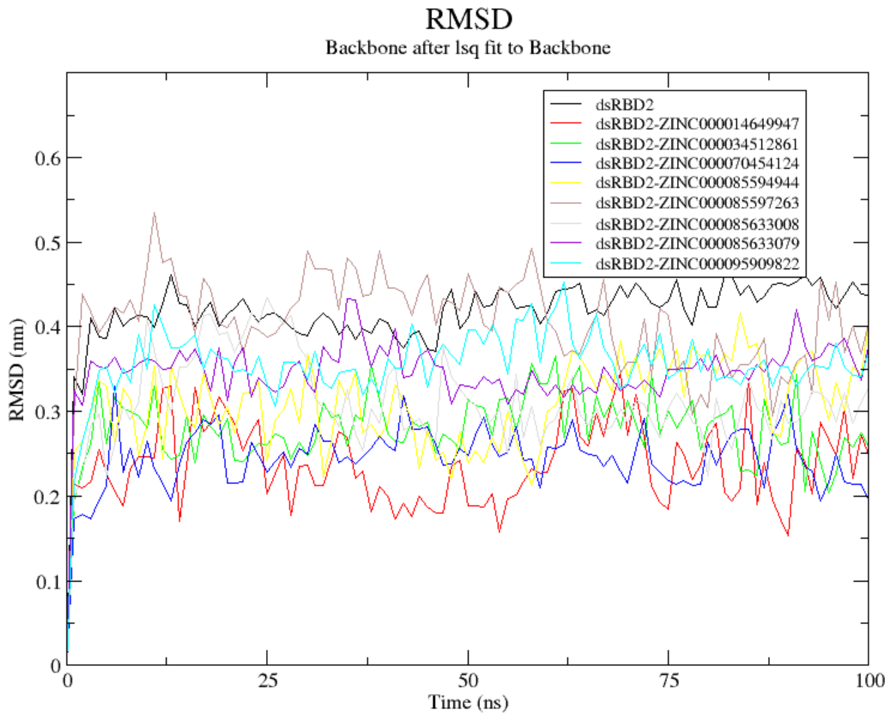

a)

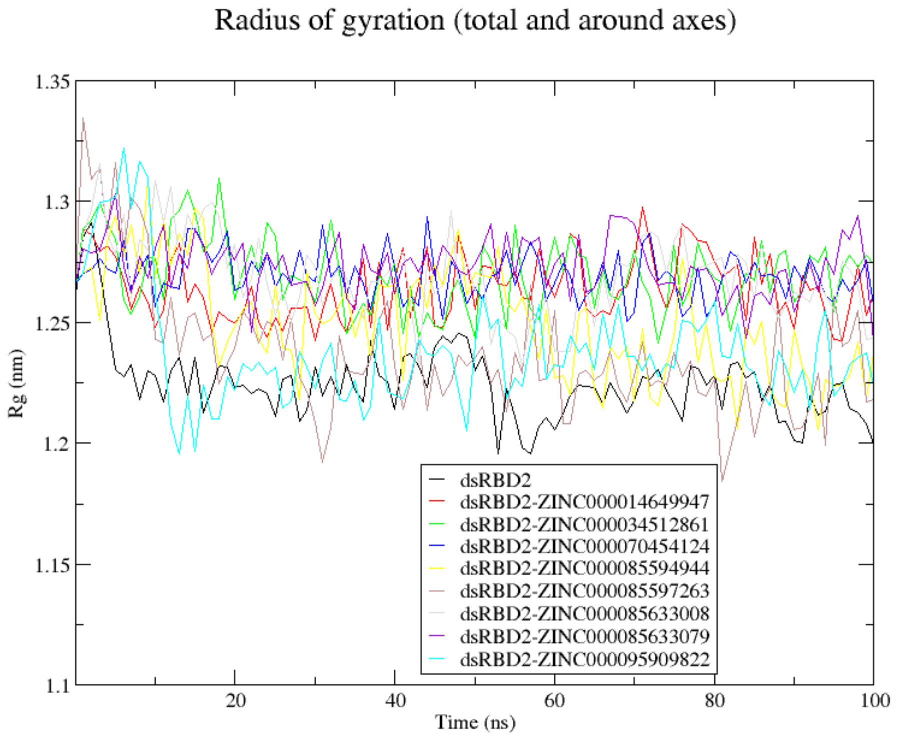

b)

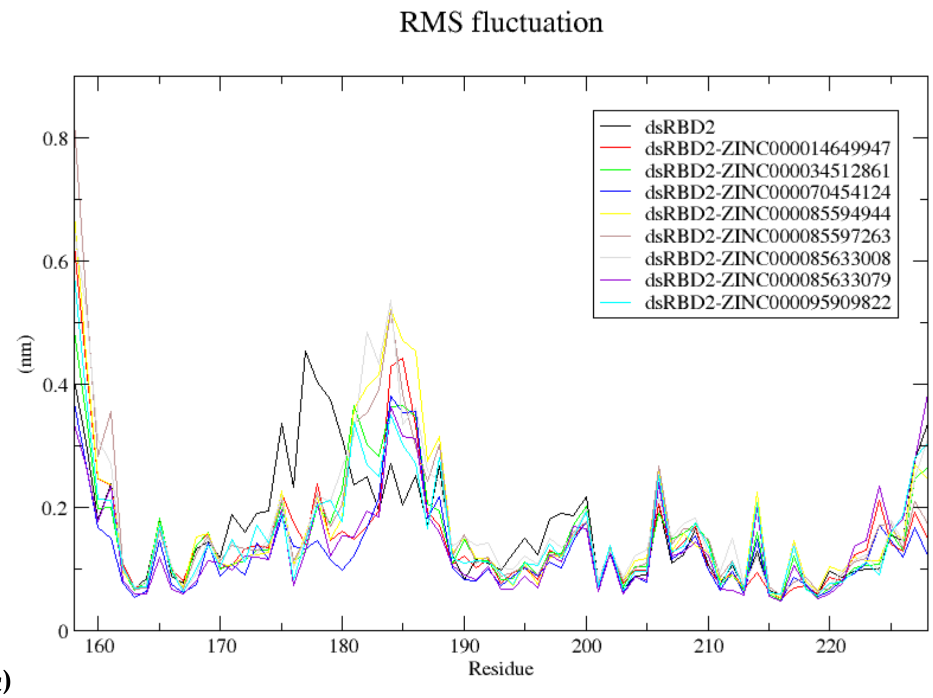

c)

Supplementary Figure 1. a) RMSD, **b)** Rg, **c)** RMSD plots of unbound ADAR2 dsRBD2 (protein) and protein-ligand complexes of 8 identified compounds with dsRBD2.

Supplementary Table 4. From protein-ligand MD simulations, RMSD and Rg averages of 11 compounds and unbound ADAR2 dsRBD2. Measurements in nanometers.

Compound	RMSD	Rg
dsRBD2	0.4158 ± 0.0485	1.225 ± 0.0156
ZINC000003203078	0.2455 ± 0.0509	1.266 ± 0.0133
ZINC000014649947	0.2358 ± 0.0513	1.264 ± 0.0131
ZINC000034512861	0.2787 ± 0.0442	1.270 ± 0.0145
ZINC000044305204	0.2529 ± 0.0419	1.270 ± 0.0132
ZINC000070454124	0.2440 ± 0.0391	1.269 ± 0.0093
ZINC000085532515	0.3155 ± 0.0484	1.243 ± 0.0338
ZINC000085594944	0.3086 ± 0.0552	1.250 ± 0.0228
ZINC000085597263	0.4044 ± 0.0627	1.237 ± 0.0265
ZINC000085633008	0.3105 ± 0.0566	1.269 ± 0.0168
ZINC000085633079	0.3461 ± 0.0421	1.238 ± 0.0113
ZINC000095909822	0.3554 ± 0.0479	1.238 ± 0.0241

Supplementary Table 5. From RNA-protein-ligand MD simulations, RMSD and Rg averages of 8 compounds, dsRBD2-GluR-2, and unbound ADAR2 dsRBD2. Measurements in nanometers.

Compound	RMSD	Rg
dsRBD2	0.4158 ± 0.0485	1.225 ± 0.0156
dsRBD2-GluR-2	0.3008 ± 0.0443	1.279 ± 0.0188
ZINC000014649947	0.3324 ± 0.0443	1.276 ± 0.0137
ZINC000034512861	0.3203 ± 0.0590	1.276 ± 0.0159
ZINC000070454124	0.3231 ± 0.0526	1.265 ± 0.0174
ZINC000085594944	0.3541 ± 0.0535	1.259 ± 0.0257
ZINC000085597263	0.2591 ± 0.0456	1.286 ± 0.0141
ZINC000085633008	0.2908 ± 0.0484	1.271 ± 0.0133
ZINC000085633079	0.3050 ± 0.0586	1.288 ± 0.0162
ZINC000095909822	0.2863 ± 0.0445	1.256 ± 0.0118

Supplementary Table 6. Binding energies from MM/PBSA calculations of 3 technical replicates of protein-ligand MD simulations for four lead compounds ZINC000085597263, ZINC000085633079, ZINC000014649947, and ZINC000034512861 and control ZINC000095909822.

Compound	Binding Energy 1	Binding Energy 2	Binding Energy 3	Average
ZINC000014649947	-91.971 ± 2.264	-89.541 ± 2.516	-99.452 ± 2.109	-93.65466667 ± 2.296333333
ZINC000034512861	-117.174 ± 1.991	-113.793 ± 2.119	-89.787 ± 2.720	-106.918 ± 2.2766667
ZINC000085597263	-115.873 ± 3.902	-136.121 ± 2.434	-102.514 ± 2.964	-118.16933 ± 3.1
ZINC000085633079	-136.667 ± 1.779	-117.433 ± 2.712	-127.421 ± 2.134	-127.17367 ± 2.2083333
ZINC000095909822	-66.133 ± 2.909	-65.104 ± 2.863	-84.082 ± 2.903	-71.773 ± 2.8916667

Supplementary Table 7. RMSD and Rg averages from protein-ligand MD simulations of four lead compounds ZINC000085597263, ZINC000085633079, ZINC000014649947, and ZINC000034512861 and control ZINC000095909822. Measurements in nanometers.

	RMSD				Rg			
Compound	1	2	3	Average	1	2	3	Average
ZINC000014649947	0.2358 ± 0.0513	0.3039 ± 0.0636	0.2378 ± 0.0473	0.2592 ± 0.0540	1.264 ± 0.0131	1.271 ± 0.0112	1.274 ± 0.0134	1.270 ± 0.0126
ZINC000034512861	0.2787 ± 0.0442	0.2616 ± 0.0442	0.2810 ± 0.0489	0.2738 ± 0.0473	1.270 ± 0.0145	1.266 ± 0.0134	1.2732 ± 0.0137	1.270 ± 0.0139
ZINC000085597263	0.4044 ± 0.0627	0.3417 ± 0.0564	0.2709 ± 0.0581	0.3390 ± 0.0591	1.237 ± 0.0265	1.279 ± 0.0170	1.272 ± 0.0172	1.263 ± 0.0202
ZINC000085633079	0.3461 ± 0.0421	0.2978 ± 0.0494	0.2699 ± 0.0455	0.3046 ± 0.0457	1.273 ± 0.0113	1.279 ± 0.0210	1.280 ± 0.0121	1.278 ± 0.0148
ZINC000095909822	0.3554 ± 0.0479	0.2954 ± 0.0786	0.2718 ± 0.0515	0.3076 ± 0.0593	1.238 ± 0.0241	1.2670 ± 0.0125	1.273 ± 0.0129	1.260 ± 0.0165

Supplementary Table 8. Binding energies from MM/PBSA calculations of 3 technical replicates of RNA-protein-ligand MD simulations for four lead compounds ZINC000085597263, ZINC000085633079, ZINC000014649947, and ZINC000034512861 and control ZINC000095909822.

Compound	Binding Energy 1	Binding Energy 2	Binding Energy 3	Average
ZINC000014649947	-48.847 ± 2.416	-50.350 ± 2.598	-57.501 ± 2.565	-52.23266667 ± 2.523333333
ZINC000034512861	-3.185 ± 9.674	10.930 ± 10.277	-46.693 ± 6.987	-12.982667 ± 8.9793333
ZINC000085597263	-177.129 ± 3.290	-235.013 ± 2.929	-233.206 ± 2.562	-215.116 ± 2.927
ZINC000085633079	-148.844 ± 3.463	-135.491 ± 2.998	-152.198 ± 2.410	-145.511 ± 2.957
ZINC000095909822	1552.963 ± 6.200	1493.020 ± 5.772	1542.187 ± 6.469	1529.39 ± 6.147

Supplementary Table 9. RMSD, Rg, Hydrogen bond averages from RNA-protein-ligand MD simulations of four lead compounds ZINC000085597263, ZINC000085633079, ZINC000014649947, and ZINC000034512861 and control ZINC000095909822. Measurements for RMSD and Rg in nanometers. Hbond cutoff is 0.35 nm.

	RMSD				Rg				Hbond			
Compound	1	2	3	Average	1	2	3	Average	1	2	3	Average
ZINC000014649947	0.3324 ± 0.0443	0.3471 ± 0.0677	0.2387 ± 0.0514	0.3061 ± 0.0545	1.276 ± 0.0137	1.244 ± 0.0255	1.269 ± 0.0131	1.263 ± 0.0174	0.0792 ± 0.2701	0 ± 0	0.0396 ± 0.1950	0.0396 ± 0.1550
ZINC000034512861	0.3203 ± 0.0590	0.2879 ± 0.0554	0.3035 ± 0.0498	0.3039 ± 0.0547	1.276 ± 0.0159	1.274 ± 0.0115	1.265 ± 0.0125	1.272 ± 0.0133	0.8812 ± 0.6791	1.604 ± 0.9019	1.089 ± 0.7459	1.191 ± 0.7756
ZINC000085597263	0.2591 ± 0.0456	0.2253 ± 0.0336	0.3945 ± 0.0841	0.2929 ± 0.0544	1.286 ± 0.0141	1.258 ± 0.0149	1.285 ± 0.0201	1.276 ± 0.0164	0.0198 ± 0.1393	0 ± 0	0 ± 0	0.0066 ± 0.0464
ZINC000085633079	0.3050 ± 0.0586	0.3767 ± 0.0625	0.3195 ± 0.0655	0.3337 ± 0.0622	1.288 ± 0.0162	1.319 ± 0.0196	1.278 ± 0.0136	1.295 ± 0.0165	0.5248 ± 0.5557	0.9010 ± 0.2987	0.1980 ± 0.3985	0.5413 ± 0.4176
ZINC000095909822	0.2863 ± 0.0445	0.2866 ± 0.0585	0.3114 ± 0.0446	0.2937 ± 0.0492	1.256 ± 0.0118	1.263 ± 0.0110	1.293 ± 0.0103	1.270 ± 0.0110	0.6040 ± 0.6760	0.8911 ± 0.7948	1.149 ± 0.9269	0.8812 ± 0.7992

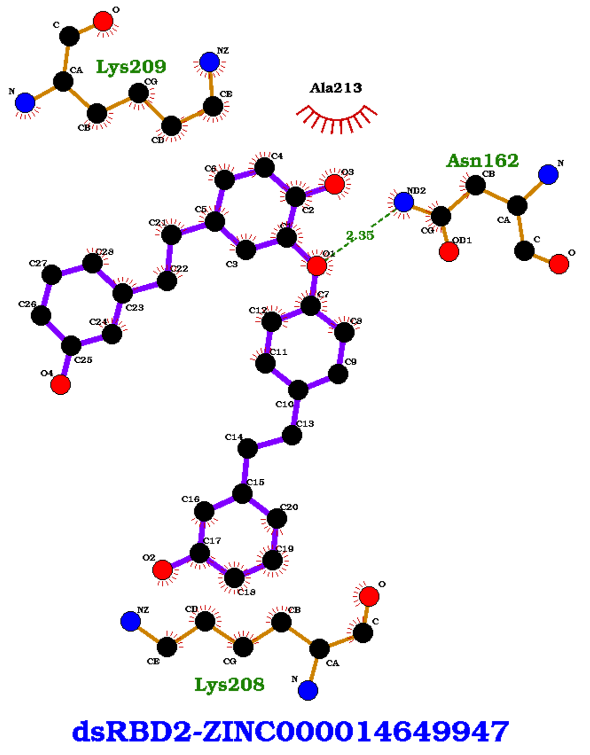
Lys209
Ala213
Asn162
2.35
Lys208
dsRBD2-ZINC000014649947

a)

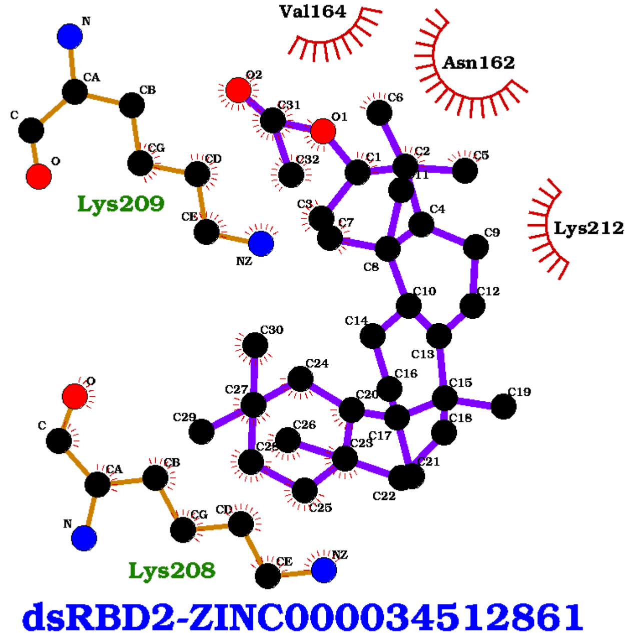
Val164
Asn162
Lys209
Lys212
Lys208
dsRBD2-ZINC000034512861

b)

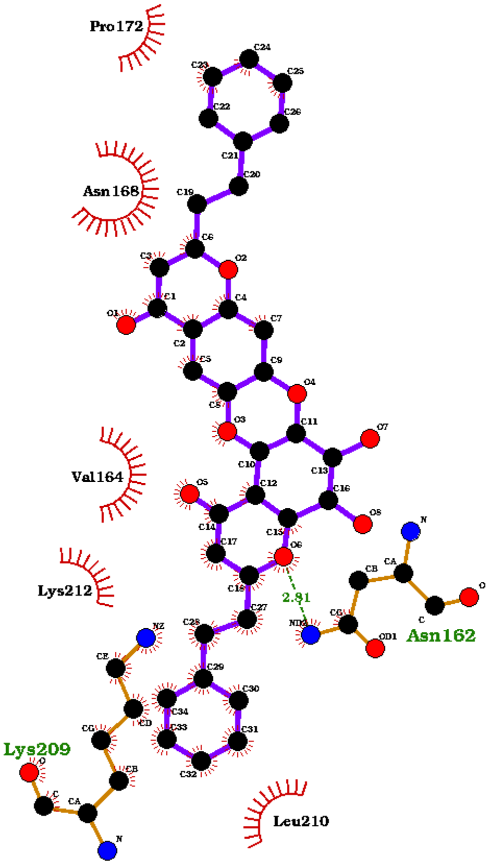

c) **dsRBD2-ZINC000070454124**

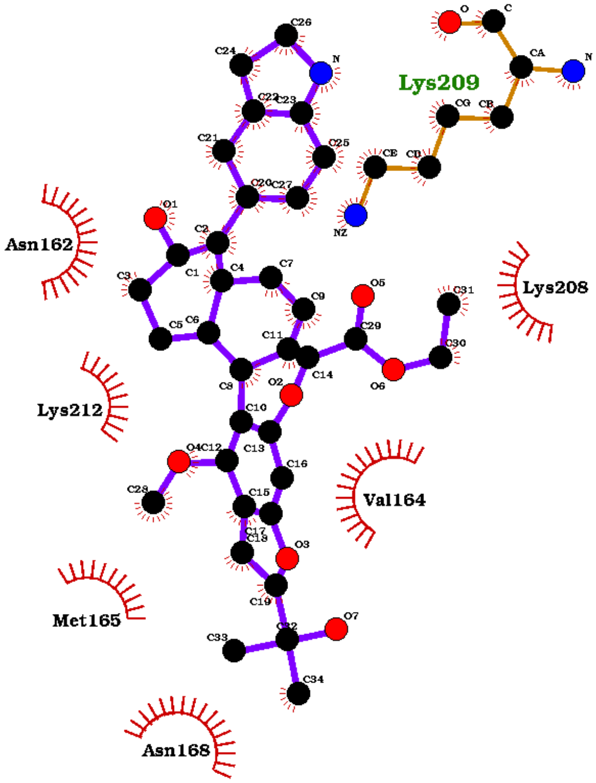

d) **dsRBD2-ZINC000085594944**

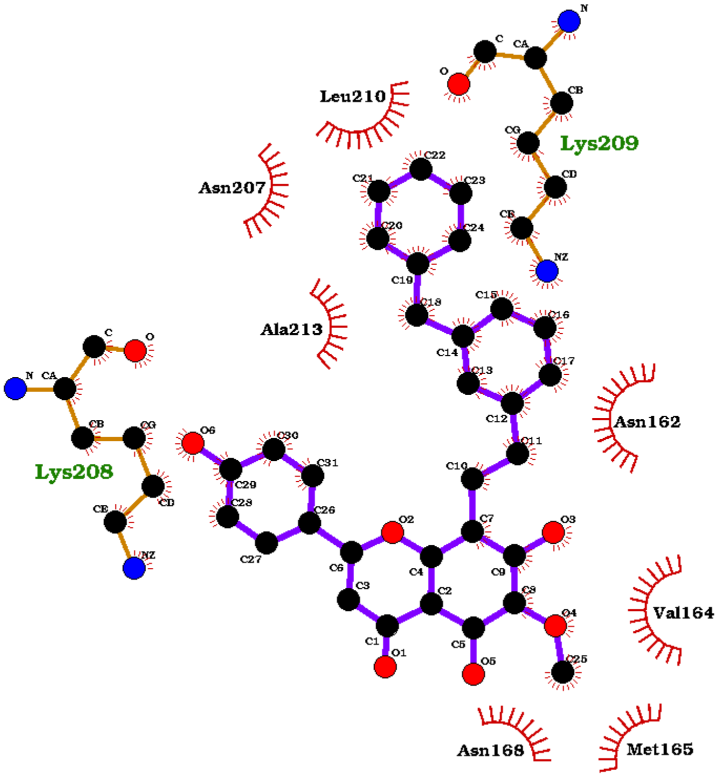

e) dsRBD2-ZINC000085633008

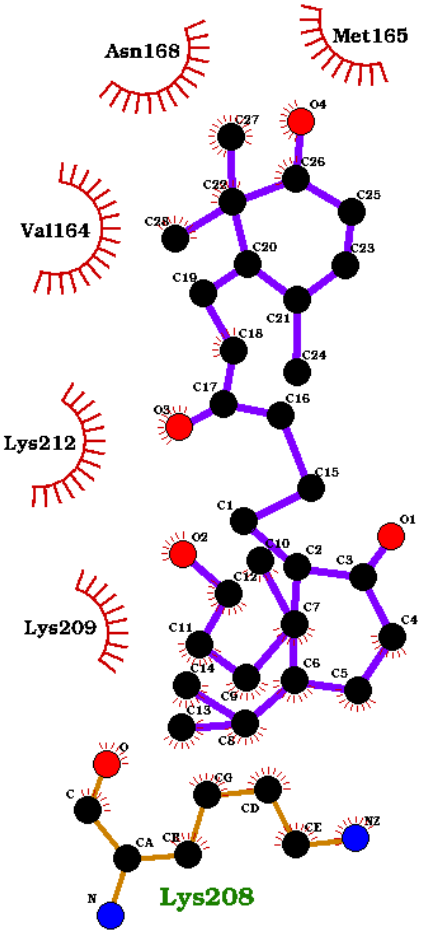

f) dsRBD2-ZINC000095909822

Supplementary Figure 2. Protein ligand interaction plots between dsRBD2 and compounds **a)** ZINC000014649947, **b)** ZINC000034512861, **c)** ZINC000070454124, **d)** ZINC000085594944, **e)** ZINC000085633008, and **f)** ZINC000095909822. Color code for PLIPs are: carbon (black), oxygen (red), nitrogen (blue), sulfur (yellow). Dashed green lines represent hbonds with the length listed. Hydrophobic bonds correspond to red coronas.

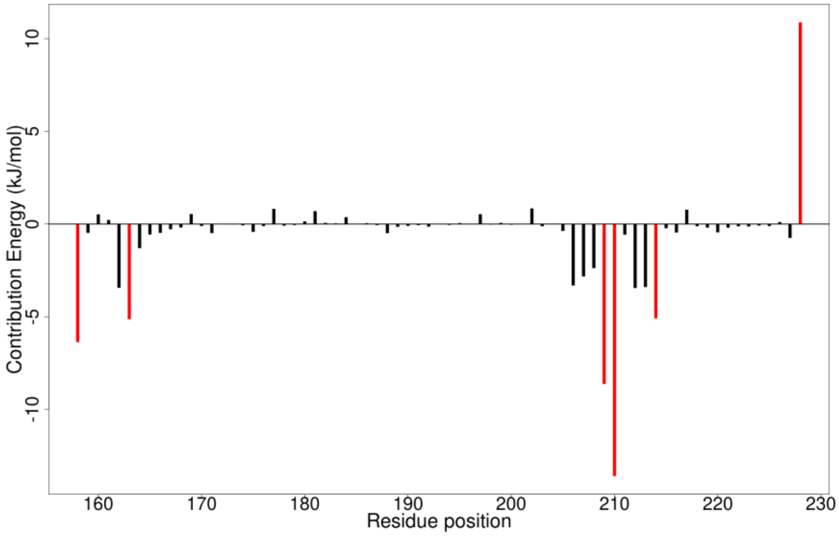

a)

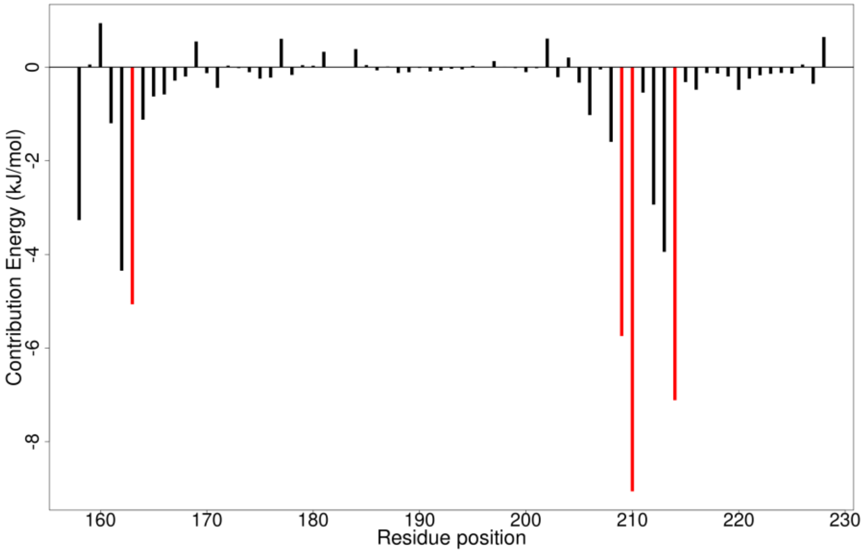

b)

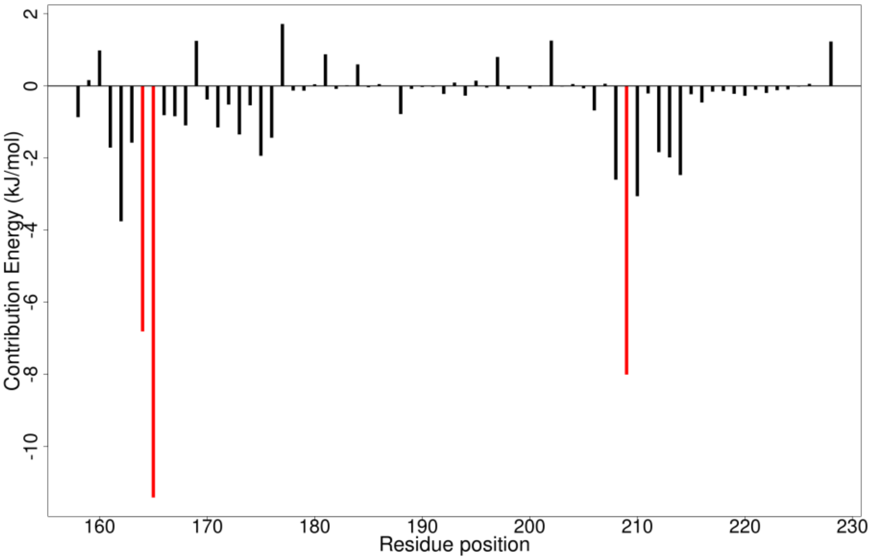
Contribution Energy (kJ/mol)
Residue position
2
0
-2
-4
-6
-8
-10
160
170
180
190
200
210
220
230
c)

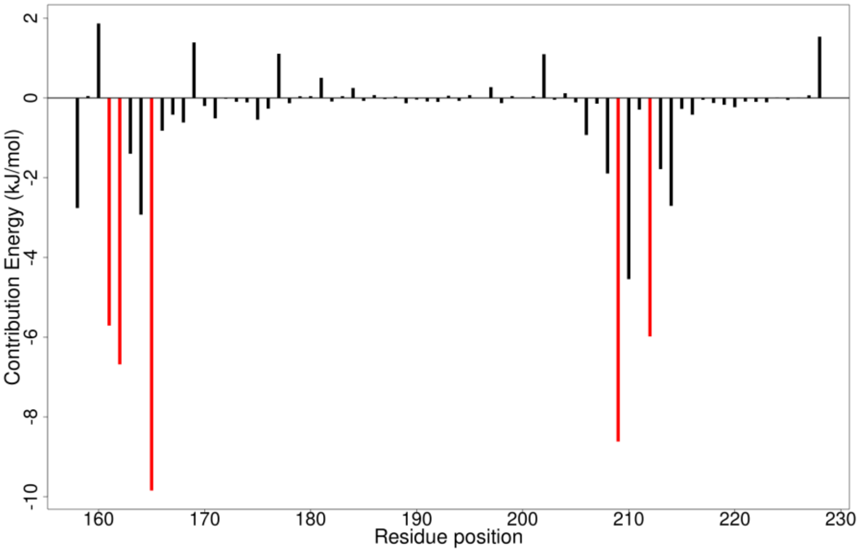
Contribution Energy (kJ/mol)
Residue position
2
0
-2
-4
-6
-8
-10
160
170
180
190
200
210
220
230
d)

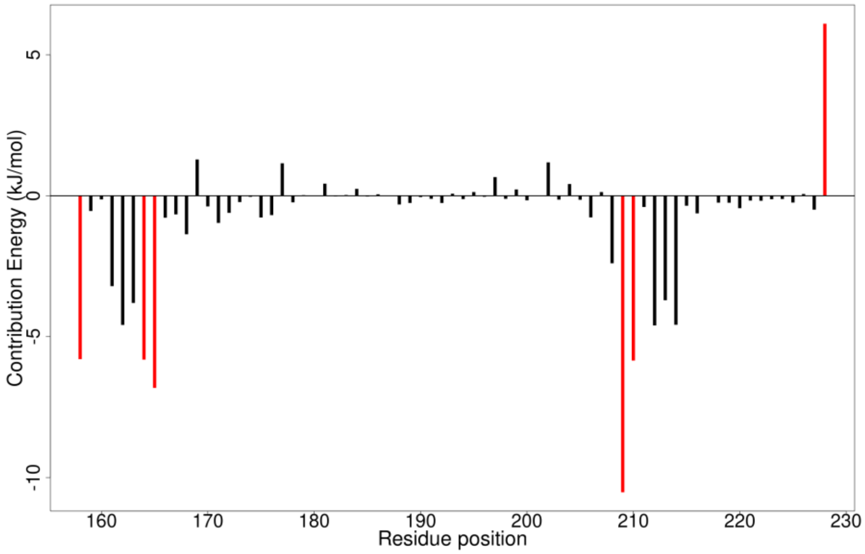

Contribution Energy (kJ/mol)
5
0
-5
-10
160
170
180
190
200
210
220
230
Residue position

e)

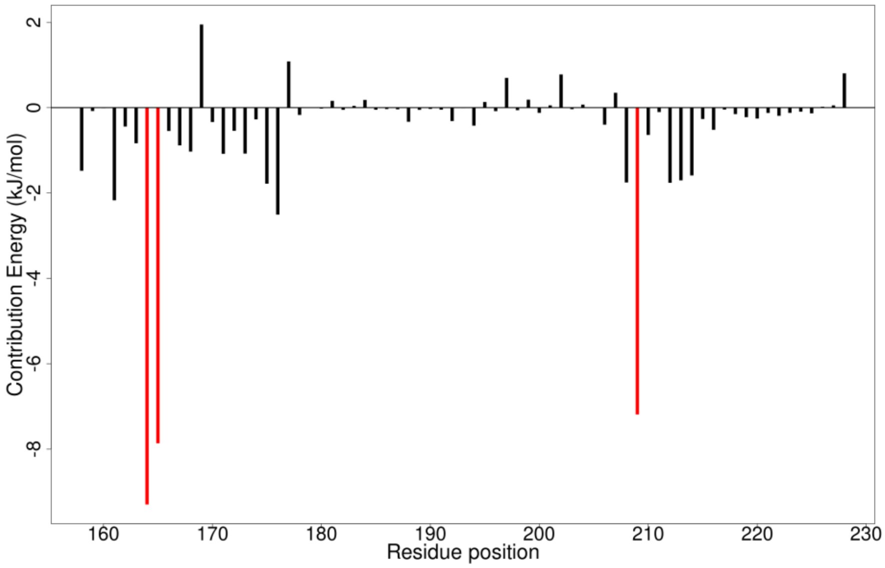

Contribution Energy (kJ/mol)
2
0
-2
-4
-6
-8
160
170
180
190
200
210
220
230
Residue position

f)

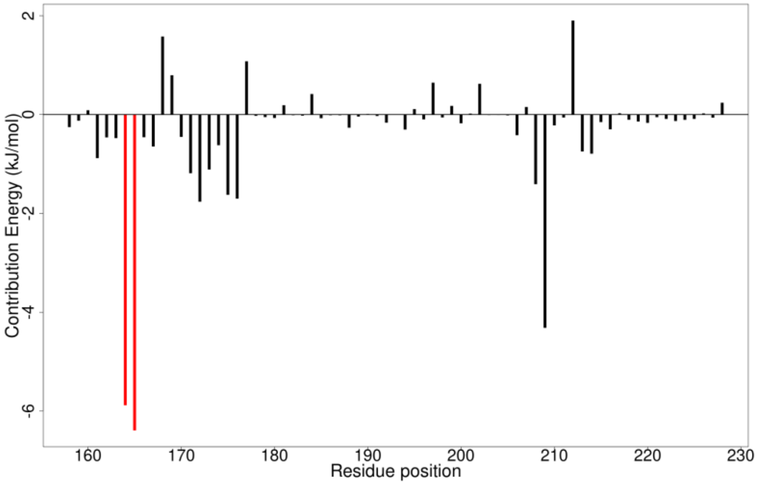

g)

Supplementary Figure 3. Per residue energy contribution maps from protein-ligand MD simulations between dsRBD2 and compounds **a)** ZINC000014649947, **b)** ZINC000034512861, **c)** ZINC000070454124, **d)** ZINC000085594944, **e)** ZINC000085597263, **f)** ZINC000085633008, and **g)** ZINC000095909822.

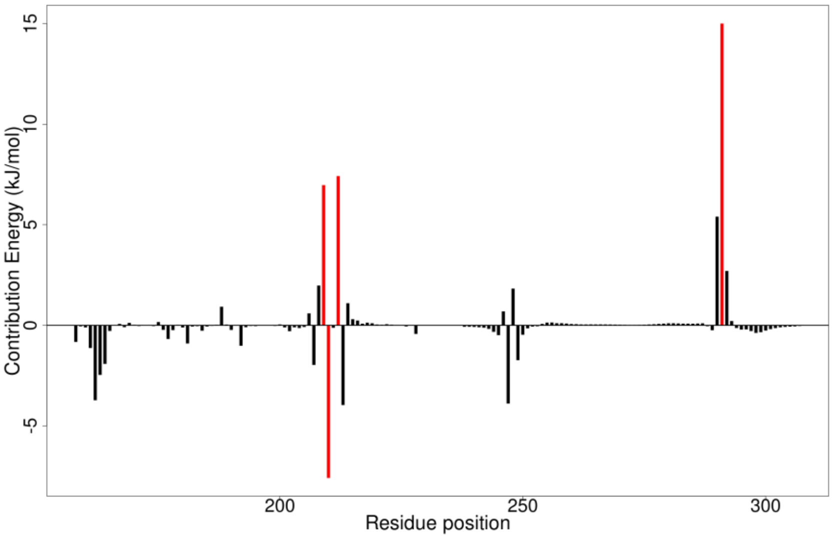

a)

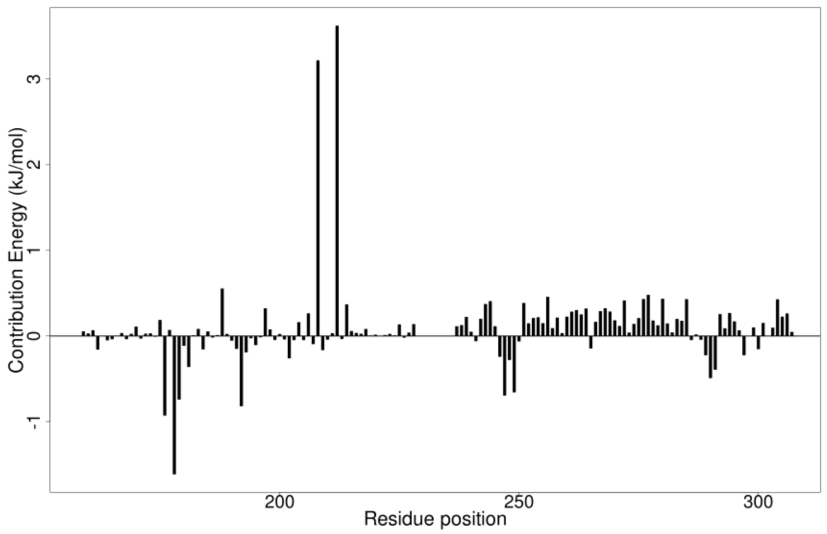

Contribution Energy (kJ/mol)
3
2
1
0
-1
200
250
300
Residue position

b)

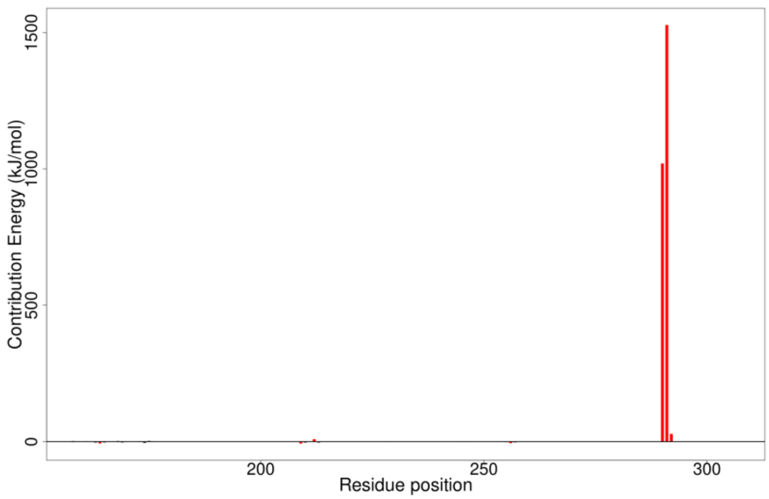

Contribution Energy (kJ/mol)
1500
1000
500
0
200
250
300
Residue position

c)

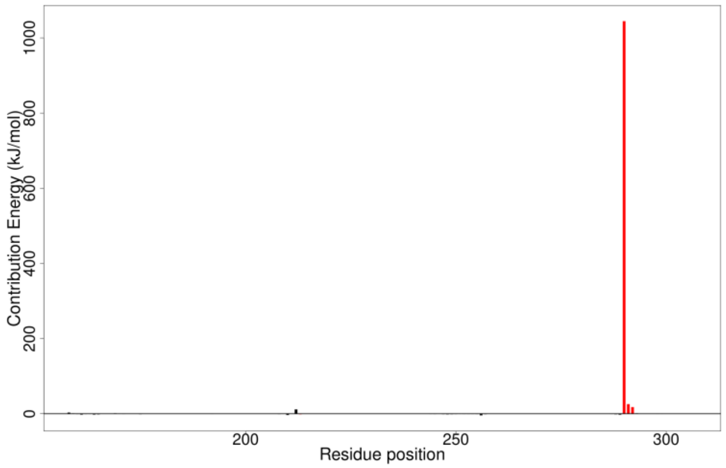

Contribution Energy (kJ/mol)
1000
800
600
400
200
0
200
250
300
Residue position

d)

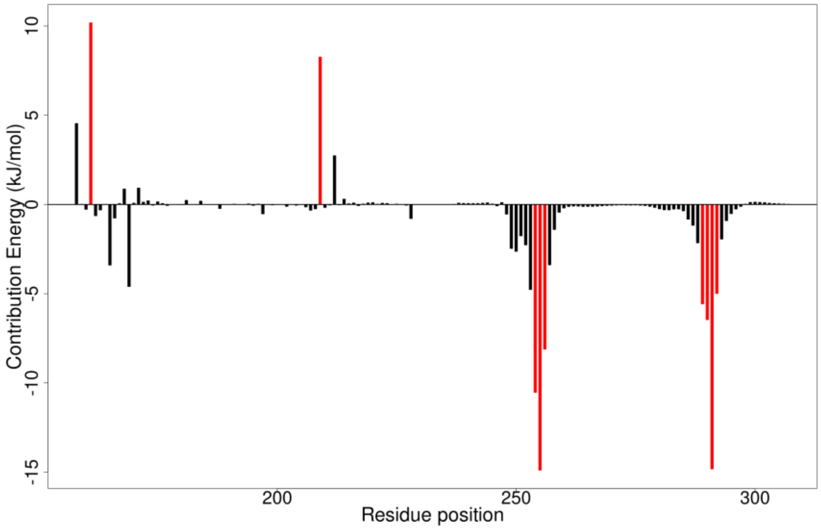

Contribution Energy (kJ/mol)
10
5
0
-5
-10
-15
200
250
300
Residue position

e)

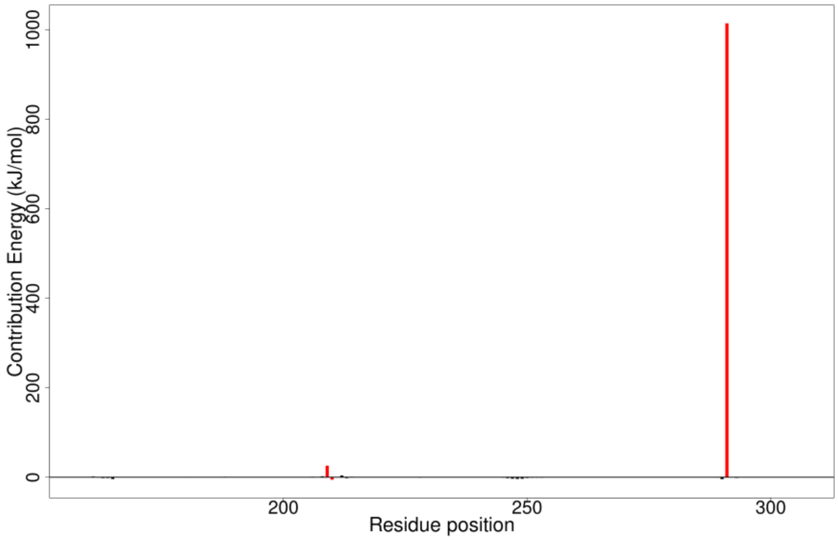

Contribution Energy (kJ/mol)
1000
800
600
400
200
0
200
250
300
Residue position

f)

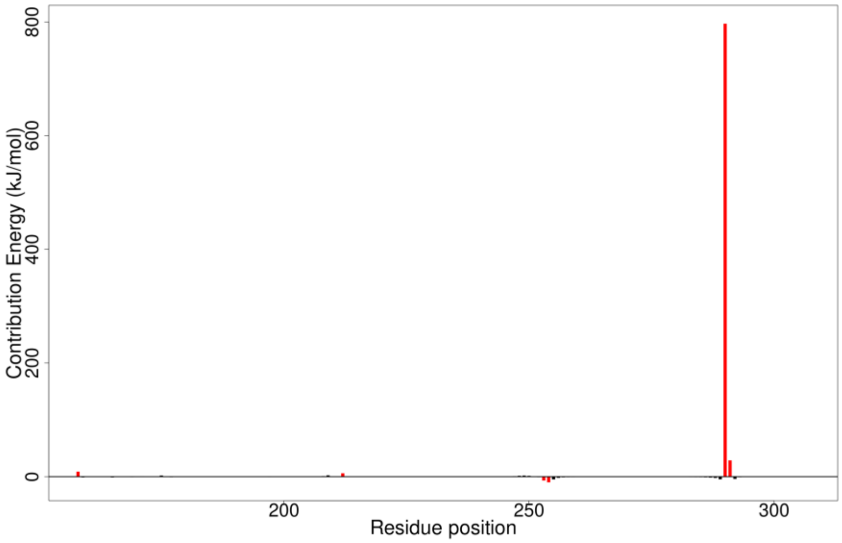

Contribution Energy (kJ/mol)
800
600
400
200
0
200
250
300
Residue position

g)

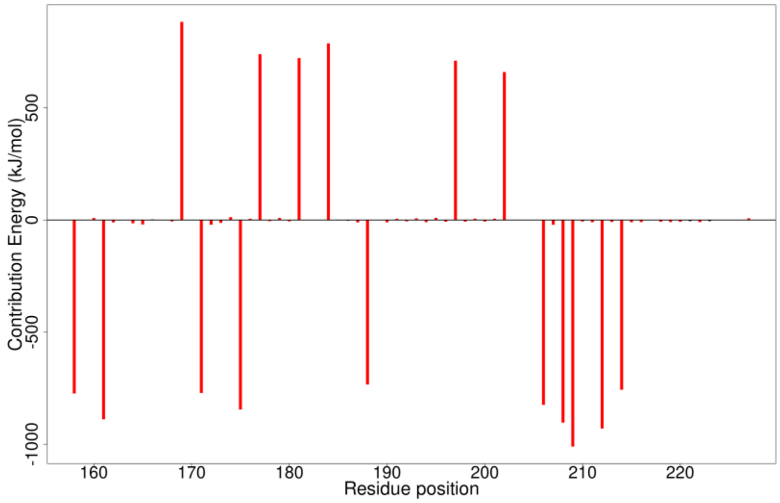

h)

Supplementary Figure 4. Per residue energy contribution maps from RNA-protein-ligand MD simulations between dsRBD2 and compounds **a)** ZINC000014649947, **b)** ZINC000034512861, **c)** ZINC000070454124, **d)** ZINC000085594944, **e)** ZINC000085597263, **f)** ZINC000085633008, **g)** ZINC000095909822, **h)** GluR-2 RNA.

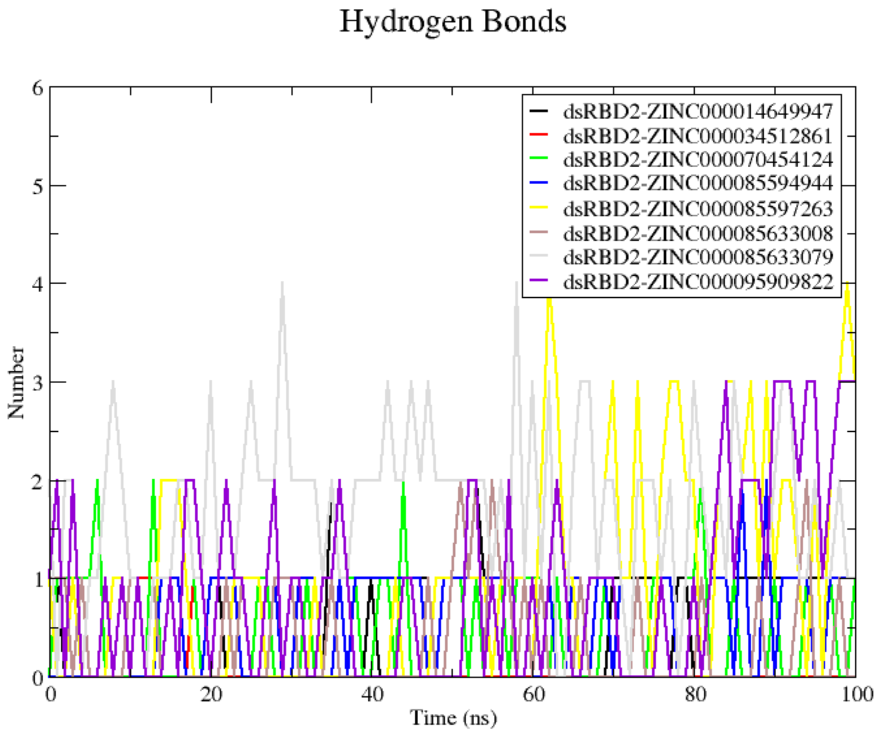

a)

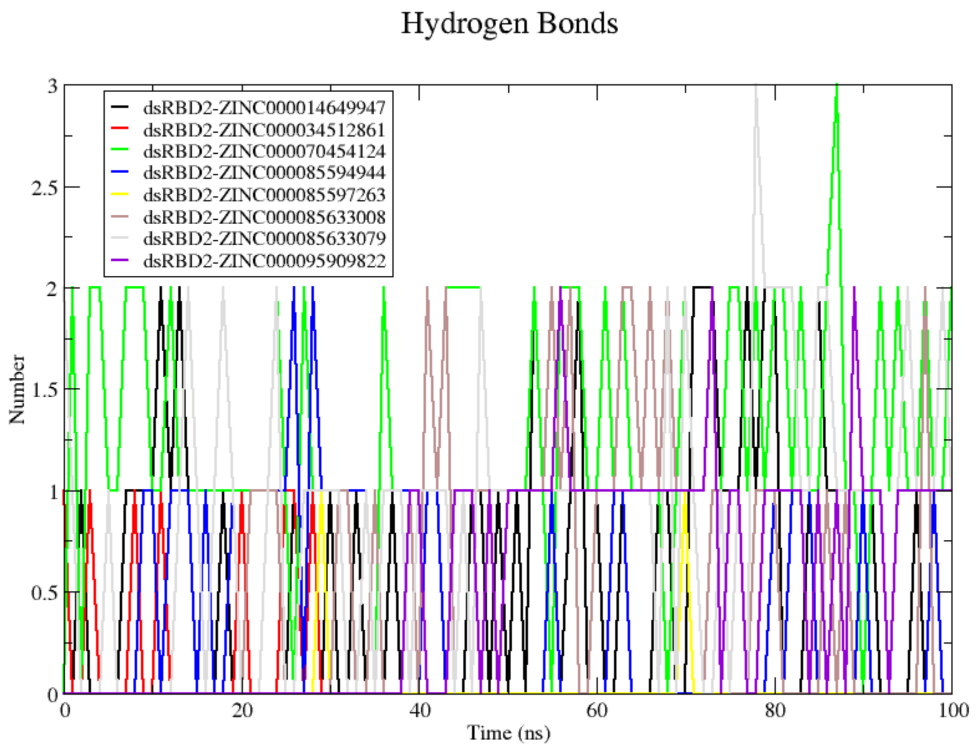

b)

Supplementary Figure 5. Hydrogen bonds calculated between dsRBD2 and compounds ZINC000014649947, ZINC000034512861, ZINC000070454124, ZINC000085594944, ZINC000085597263, ZINC000085633008, ZINC000085633079, and ZINC000095909822 using gmx hbond for **a)** protein-ligand simulations and **b**) RNA-protein-ligand simulations. Hbonds pairs are within 0.35 nm.

Supplementary Table 10. PASS prediction for compound ZINC000014649947.

**ZINC000014649947**		
**Biologic Activity**	**Pa**	**Pi**
Steroid N-acetylglucosaminyltransferase inhibitor	0.787	0.003
Preneoplastic conditions treatment	0.757	0.005
Glutathione thiolesterase inhibitor	0.749	0.012
Methylenetetrahydrofolate reductase (NADPH) inhibitor	0.597	0.038
Peroxidase inhibitor	0.741	0.008
G-protein-coupled receptor kinase inhibitor	0.658	0.027
Beta-adrenergic receptor kinase inhibitor	0.634	0.004
Thioredoxin inhibitor	0.646	0.019
Pin1 inhibitor	0.633	0.014
Antimutagenic	0.628	0.009
Protein-disulfide reductase (glutathione) inhibitor	0.646	0.029
Oxidoreductase inhibitor	0.641	0.025
Lysase inhibitor	0.636	0.035
P-benzoquinone reductase (NADPH) inhibitor	0.598	0.011
NADPH-ferrihemoprotein reductase inhibitor	0.600	0.014
Formate-dihydrofolate ligase inhibitor	0.525	0.009

Caspase 8 stimulant	0.497	0.018
TNF expression inhibitor	0.483	0.033
Superoxide dismutase inhibitor	0.487	0.041
Caspase 3 stimulant	0.452	0.036
Thymidylate 5'-phosphatase inhibitor	0.401	0.053
Glutathione-disulfide reductase inhibitor	0.359	0.012
Apoptosis agonist	0.407	0.070
Glutathione dehydrogenase (ascorbate) inhibitor	0.344	0.038
Anticarcinogenic	0.340	0.045
Proliferative diseases treatment	0.301	0.048
Antineoplastic (non-Hodgkin's lymphoma)	0.308	0.213

Supplementary Table 11. Results from structural similarity search for compound ZINC000014649947.

ZINC000014649947	
Similar Compound (Drug Bank Accession #)	Similarity Score
5-pentyl-2-phenoxyphenol (DB07178)	0.785
3-(3,4-dimethoxyphenyl)propanoic acid (DB04208)	0.653
Terameprocol (DB12226)	0.652
CA4P (DB05284)	0.644
Combretastatin (DB12596)	0.644
Zingerone (DB15589)	0.64
3,4,5-Trimethoxyamphetamine (DB01516)	0.634
5-[2-(4-hydroxyphenyl)ethyl]benzene-1,3-diol (DB08466)	0.631
Secoisolariciresinol (DB12179)	0.626

Supplementary Table 12. PASS prediction for compound ZINC000034512861.

ZINC000034512861		
Biologic Activity	Pa	Pi
Caspase 3 stimulant	0.946	0.003
Antineoplastic	0.922	0.005
Chemopreventive	0.915	0.002
Apoptosis agonist	0.911	0.004
Caspase 8 stimulant	0.874	0.001
Antineoplastic (lung cancer)	0.826	0.004
Antiulcerative	0.824	0.003
Antineoplastic (breast cancer)	0.742	0.005
Antineoplastic (colon cancer)	0.735	0.005
Antineoplastic (colorectal cancer)	0.734	0.005
Antineoplastic (ovarian cancer)	0.726	0.004
Antineoplastic (melanoma)	0.680	0.004
Antitoxic	0.680	0.005
Antineoplastic (thyroid cancer)	0.634	0.001

Antineoplastic (endocrine cancer)	0.603	0.002
Antineoplastic (carcinoma)	0.553	0.004
Antineoplastic (cervical cancer)	0.552	0.005
Antimetastatic	0.540	0.012
Prostate cancer treatment	0.524	0.008
Lysase inhibitor	0.431	0.085
Protein-disulfide reductase (glutathione) inhibitor	0.429	0.105
Antineoplastic (liver cancer)	0.324	0.014
Proliferative diseases treatment	0.336	0.038
Anticarcinogenic	0.319	0.051

Supplementary Table 13. Results from structural similarity search for compound ZINC000034512861.

ZINC000034512861	
Similar Compound (Drug Bank Accession #)	Similarity Score
Trestolone acetate (DB13958)	0.776
Terpinyl acetate (DB15957)	0.761
Testosterone propionate (DB01420)	0.758
Acetoxolone (DB13540)	0.754
Nandrolone decanoate (DB08804)	0.74
Testosterone cypionate (DB13943)	0.74
Testosterone enanthate (DB13944)	0.74
Testosterone undecanoate (DB13846)	0.74
Testosterone decanoate (DB16001)	0.74
Testosterone isocaproate (DB16002)	0.74
Dimethandrolone Undecanoate (DB16141)	0.74
Medroxyprogesterone acetate (DB00603)	0.724
Norgestomet (DB11440)	0.724
Pregnenolone acetate (DB14626)	0.723
SUCCINIC ACID MONO-(13-METHYL-3-OXO-2,3,6,7,8,9,10,11,12,13,14,15,16,17-TETRADECAHYDRO-1H-CYCLOPENTA[A]PHENANTHREN-17-YL) ESTER (DB08308)	0.713
Testosterone succinate (DB08619)	0.713
Drospirenone (DB01395)	0.708
Carbenoxolone (DB02329)	0.705

Supplementary Table 14. PASS prediction for compound ZINC000085597263.

ZINC000085597263		
Biologic Activity	Pa	Pi
Chemopreventive	0.805	0.004
Antineoplastic	0.623	0.040
Antineoplastic (breast cancer)	0.559	0.014
Antineoplastic (lung cancer)	0.486	0.014
Apoptosis agonist	0.479	0.044

Prostate cancer treatment	0.433	0.017
Free radical scavenger	0.430	0.015
Caspase 8 stimulant	0.424	0.040
Antineoplastic (carcinoma)	0.313	0.013
Microtubule formation inhibitor	0.308	0.014
Proliferative diseases treatment	0.329	0.039
Anticarcinogenic	0.328	0.048
Antineoplastic (non-Hodgkin's lymphoma)	0.383	0.124
Caspase 3 stimulant	0.312	0.114

Supplementary Table 15. Results from structural similarity search for compound ZINC000085597263.

ZINC000085597263	
Similar Compound (Drug Bank Accession #)	Similarity Score
Erteberel (DB07933)	0.618
(3AS,4R,9BR)-4-(4-HYDROXYPHENYL)-6-(METHOXYMETHYL)-1,2,3,3A,4,9B-HEXAHYDROCYCLOPENTA[C]CHROMEN-8-OL (DB08020)	0.606
(3AS,4R,9BR)-4-(4-HYDROXYPHENYL)-1,2,3,3A,4,9B-HEXAHYDROCYCLOPENTA[C]CHROMEN-9-OL (DB08737)	0.602
Epigallocatechin gallate (DB12116)	0.601
Epigallo Catechin Gallate (DB16120)	0.601

Supplementary Table 16. PASS prediction for compound ZINC000085633079.

ZINC000085633079		
Biologic Activity	Pa	Pi
Antineoplastic enhancer	0.997	0.001
Antineoplastic	0.927	0.005
Chemopreventive	0.651	0.008
Apoptosis agonist	0.422	0.063
Antimetastatic	0.382	0.052
Antileukemic	0.330	0.037

Supplementary Table 17. Results from structural similarity search for compound ZINC000085633079.

ZINC000085633079	
Similar Compound (Drug Bank Accession #)	Similarity Score
Elsamitrucin (DB05129)	0.640
Calanolide A (DB04886)	0.637
Icariin (DB12052)	0.635

Coumermycin A1 (DB13912)	0.634
Troxerutin (DB13124)	0.626
Novobiocin (DB01051)	0.625
Monoxerutin (DB13764)	0.619
Clorobiocin (DB03966)	0.616
Telavancin (DB06402)	0.607
Hidrosmin (DB13490)	0.606
Oritavancin (DB04911)	0.606
4-epi-vancosaminyl derivative of vancomycin (DB04431)	0.605
Diosmin (DB08995)	0.605
Rifalazil (DB04934)	0.603
Rutin (DB01698)	0.603
PEN-866 (DB16186)	0.601
Vancomycin (DB00512)	0.600

Supplementary Table 18. Summary table of quality assessment from pre-MD ADAR2 models including results of molpdf rankings from MODELLER and results from SAVESv6.1.

Pre- MD Simulations ADAR2							
**Model**	**Molpdf**	**ERRAT**	**VERIFY3D**	**PROCHECK**			
				**Favored**	**Allowed**	**Generously Allowed**	**Disallowed**
Model 1	48846.43359	57.4315	79.03%	84.1%	11.8%	2.7%	1.3%
Model 2	46960.29297	56.1328	82.45%	84.7%	10.8%	2.2%	2.4%
Model 3	48832.88281	57.1429	76.89%	85.2%	9.9%	2.7%	2.2%
Model 4	46036.54688	60.1732	77.60%	86.7%	9.9%	2.5%	0.8%
Model 5	44881.14844	63.0592	79.89%	85.2%	8.9%	2.7%	3.2%

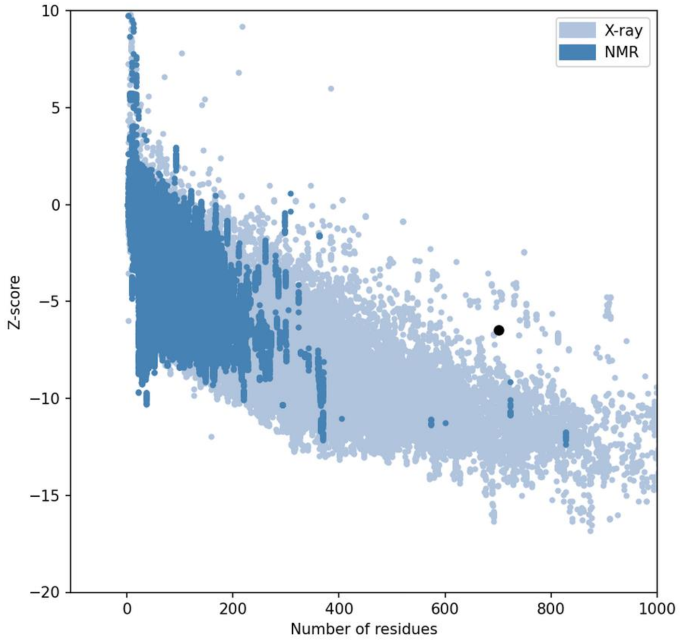

X-ray
NMR
Z-score
Number of residues
10
5
0
−5
−10
−15
−20
0
200
400
600
800
1000

a)

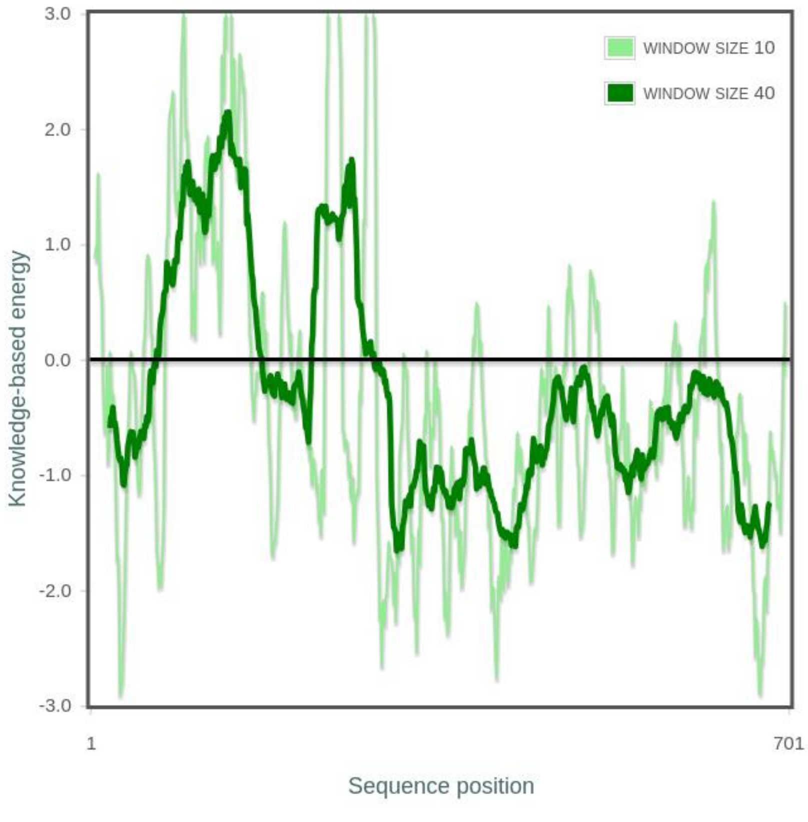

WINDOW SIZE 10
WINDOW SIZE 40
Knowledge-based energy
Sequence position
3.0
2.0
1.0
0.0
-1.0
-2.0
-3.0
1
701

b)

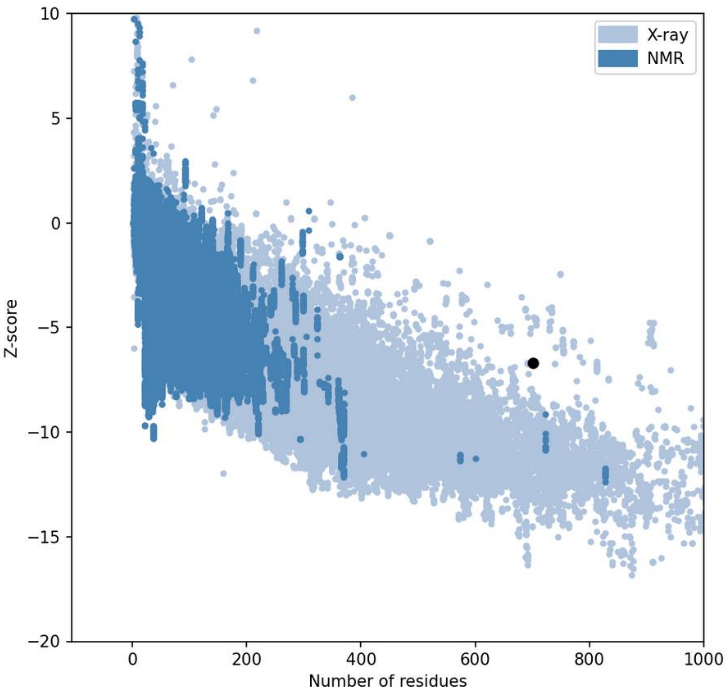
X-ray
NMR
Z-score
Number of residues
10
5
0
−5
−10
−15
−20
0
200
400
600
800
1000

c)

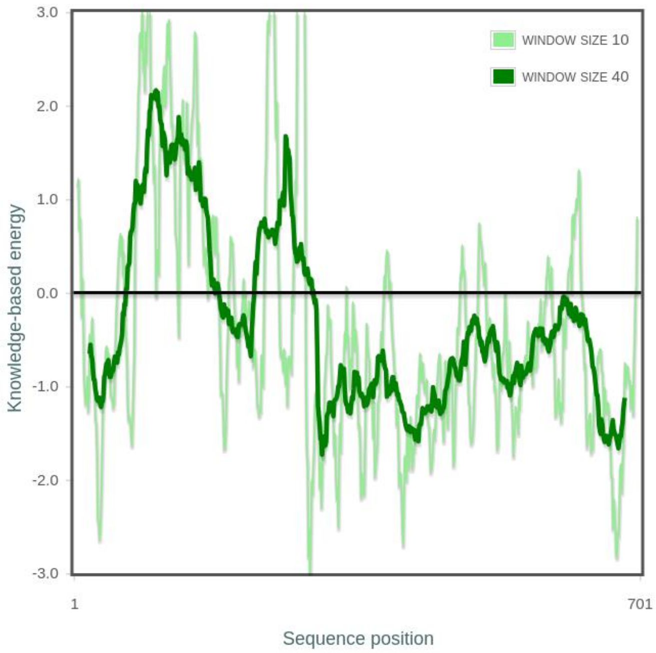
WINDOW SIZE 10
WINDOW SIZE 40
Knowledge-based energy
Sequence position
3.0
2.0
1.0
0.0
-1.0
-2.0
-3.0
1
701

d)

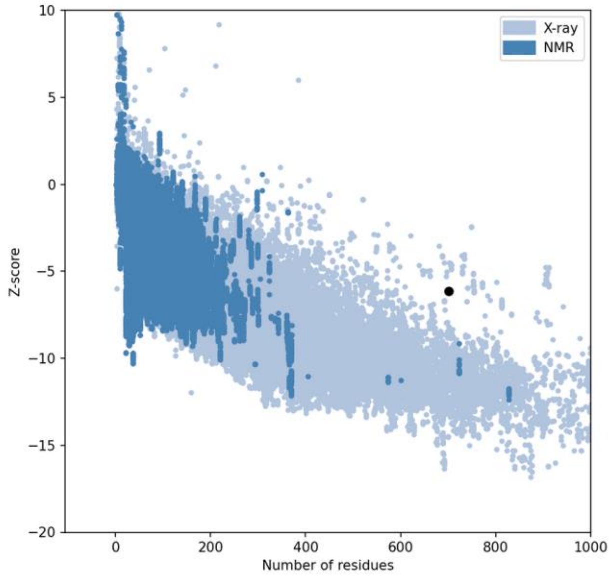
X-ray
NMR
Z-score
Number of residues
10
5
0
−5
−10
−15
−20
0
200
400
600
800
1000

e)

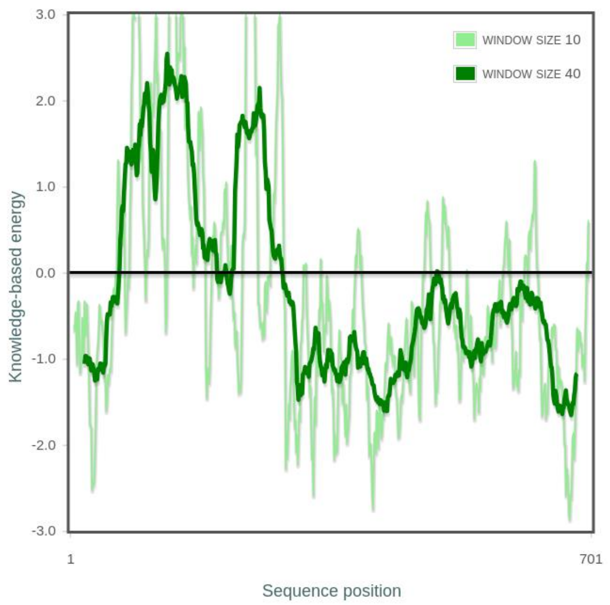
WINDOW SIZE 10
WINDOW SIZE 40
Knowledge-based energy
Sequence position
3.0
2.0
1.0
0.0
-1.0
-2.0
-3.0
1
701

f)

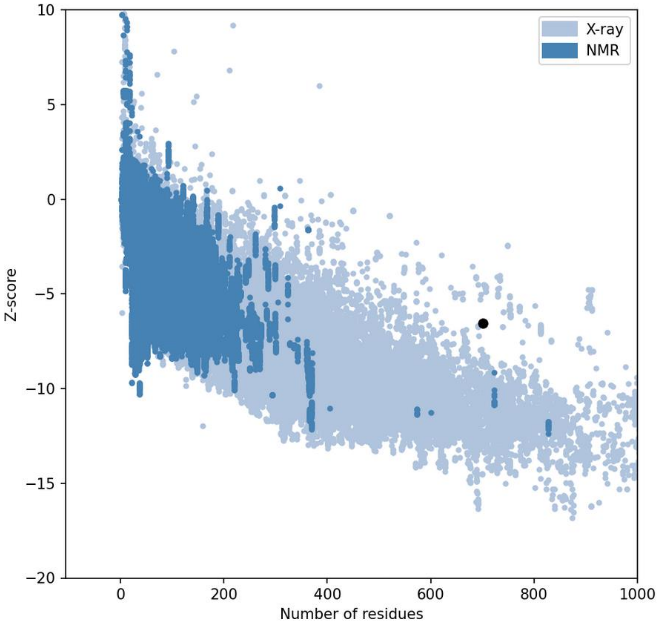
X-ray
NMR
Z-score
Number of residues

g)

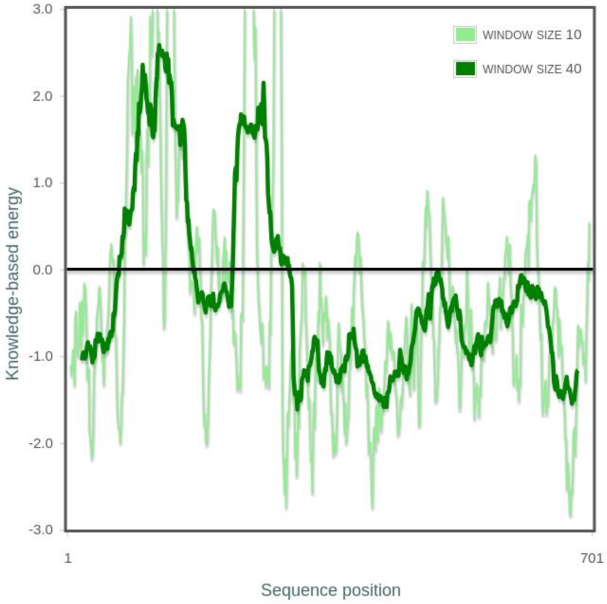
WINDOW SIZE 10
WINDOW SIZE 40
Knowledge-based energy
Sequence position

h)

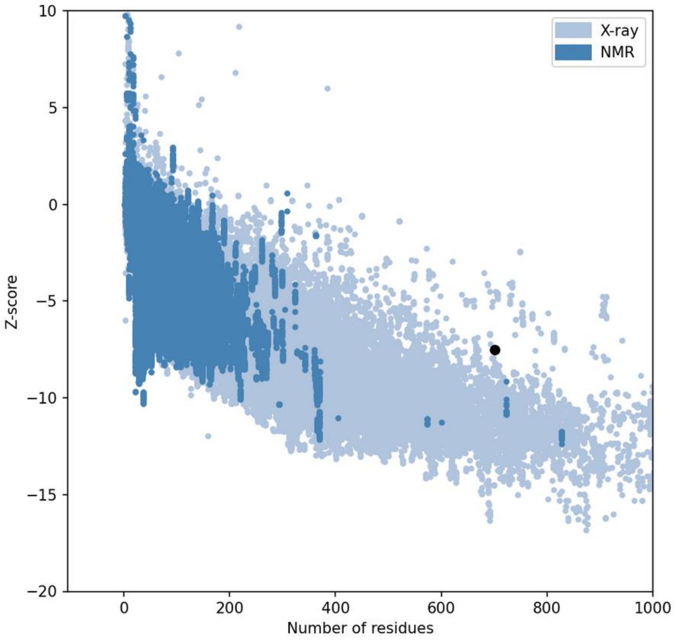

i)

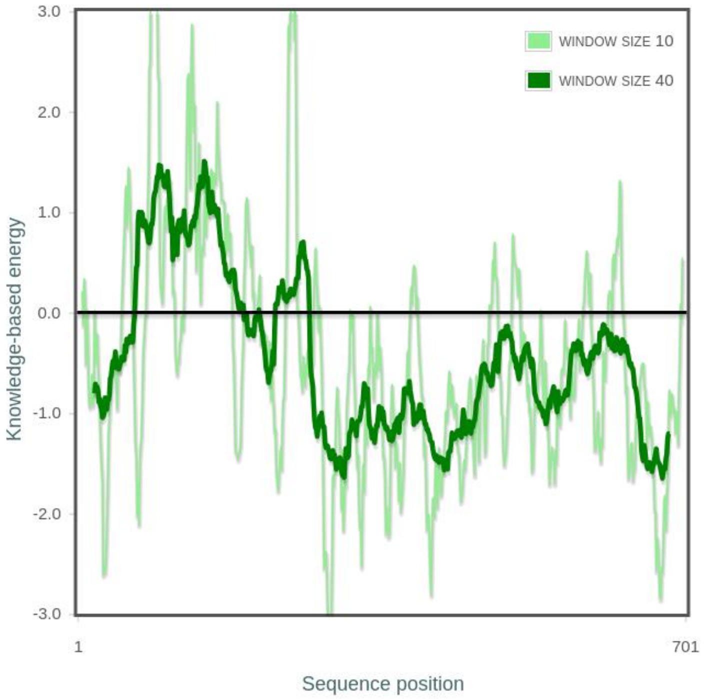

j)

Supplementary Figure 6. From ProSA, (**a**, **c**, **e**, **g**, and **i**) overall model quality plots and (**b**, **d**, **f**, **h**, and **j**) local model quality plots of 5 ADAR2 models.

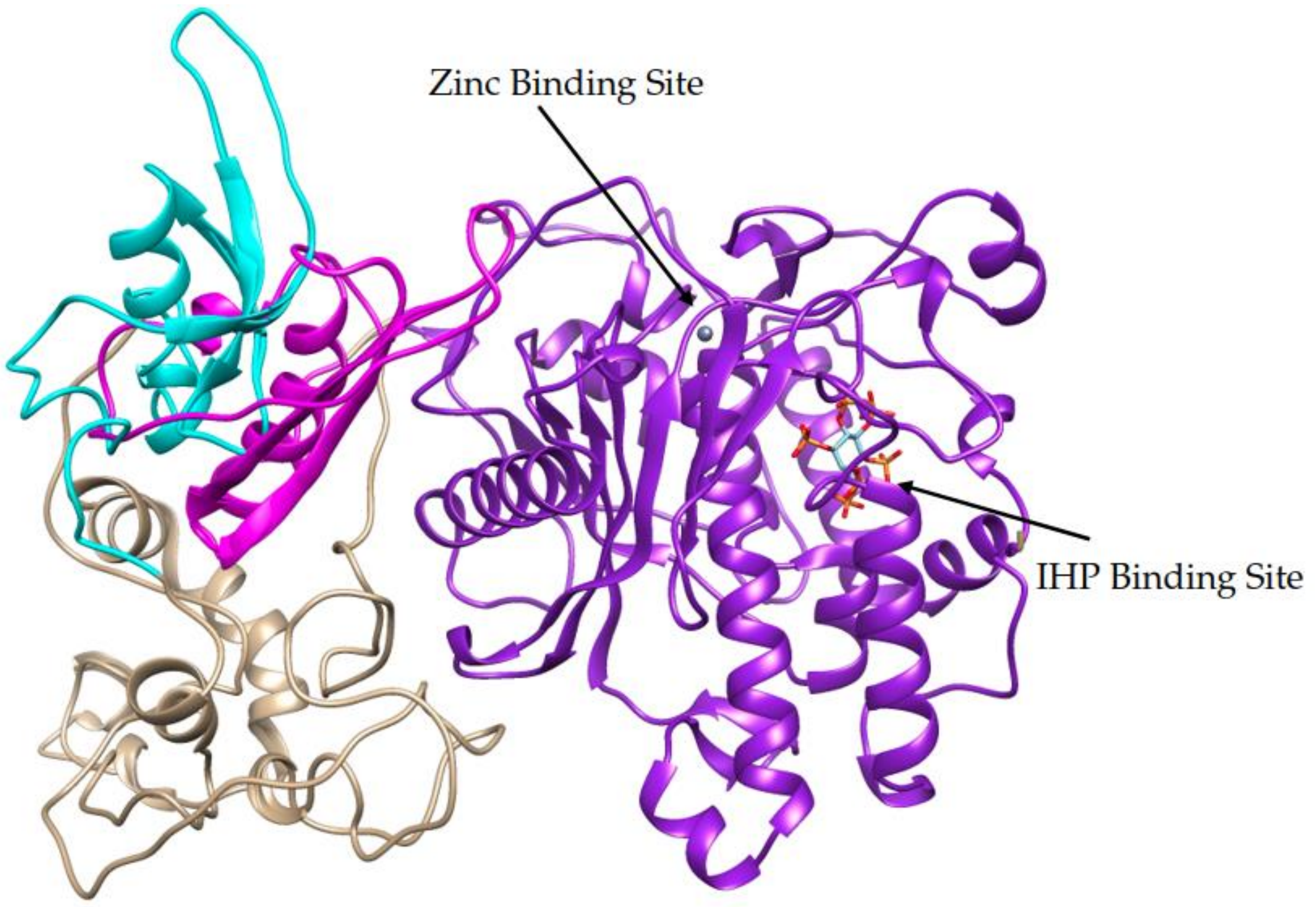

Supplementary Figure 7. Image of ADAR2 model 1 homology model. The dsRBD1 is in cyan, dsRBD2 is in magenta, and the CDD is in purple.

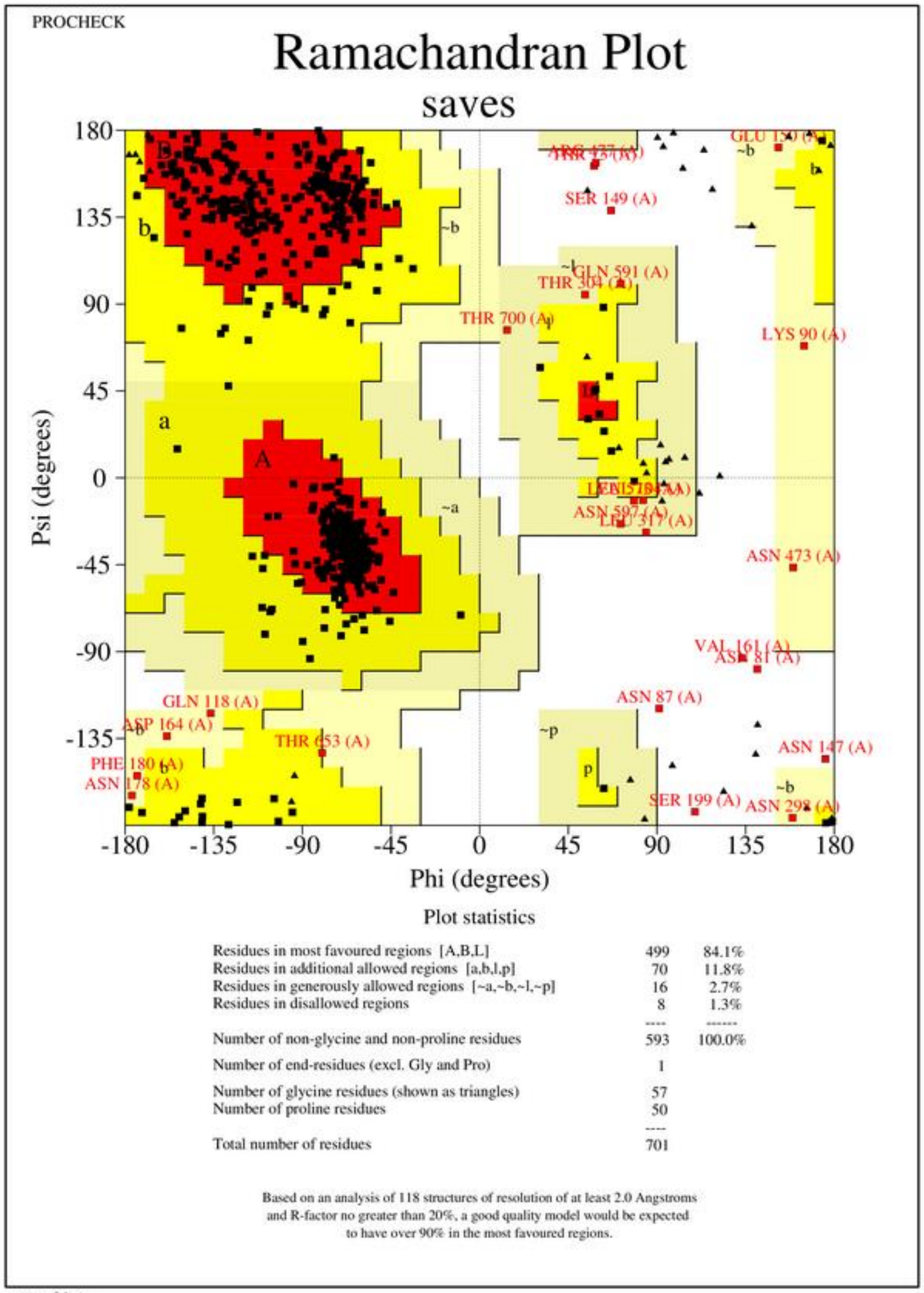
PROCHECK
Ramachandran Plot
saves
Psi (degrees)
Phi (degrees)
180
135
90
45
0
-45
-90
-135
-180
-135
-90
-45
0
45
90
135
180
SER 149 (A)
GLN 591 (A)
THR 700 (A)
LYS 90 (A)
ASN 473 (A)
ASN 87 (A)
GLN 118 (A)
THR 653 (A)
ASN 147 (A)
SER 199 (A)
ASN 178 (A)
Plot statistics
Residues in most favoured regions [A,B,L] 499 84.1%
Residues in additional allowed regions [a,b,l,p] 70 11.8%
Residues in generously allowed regions [~a,~b,~l,~p] 16 2.7%
Residues in disallowed regions 8 1.3%
Number of non-glycine and non-proline residues 593 100.0%
Number of end-residues (excl. Gly and Pro) 1
Number of glycine residues (shown as triangles) 57
Number of proline residues 50
Total number of residues 701
Based on an analysis of 118 structures of resolution of at least 2.0 Angstroms and R-factor no greater than 20%, a good quality model would be expected to have over 90% in the most favoured regions.

a) saves_01.ps

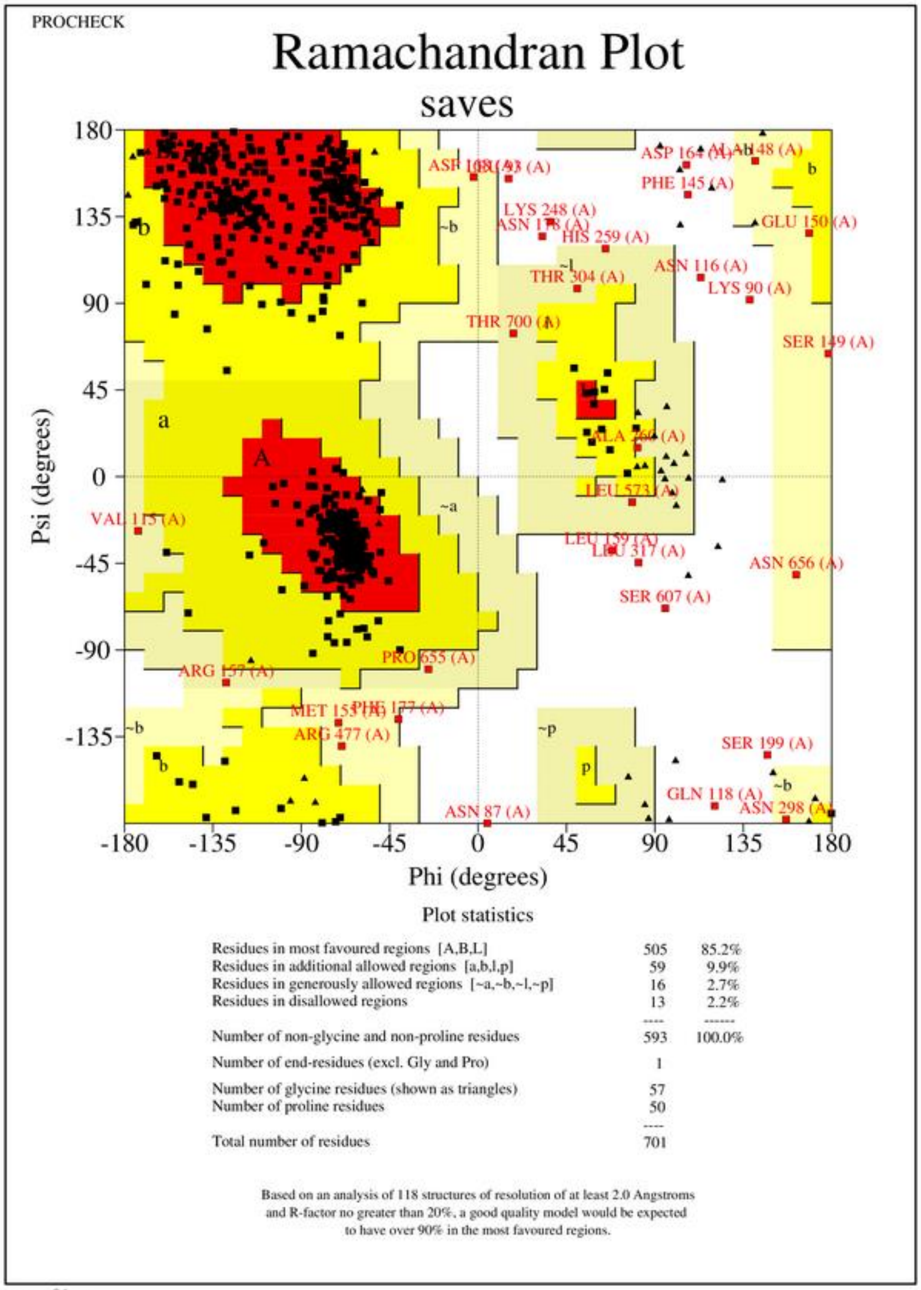
PROCHECK
Ramachandran Plot
saves
Psi (degrees)
Phi (degrees)
Plot statistics
Residues in most favoured regions [A,B,L] 505 85.2%
Residues in additional allowed regions [a,b,l,p] 59 9.9%
Residues in generously allowed regions [~a,~b,~l,~p] 16 2.7%
Residues in disallowed regions 13 2.2%
Number of non-glycine and non-proline residues 593 100.0%
Number of end-residues (excl. Gly and Pro) 1
Number of glycine residues (shown as triangles) 57
Number of proline residues 50
Total number of residues 701
Based on an analysis of 118 structures of resolution of at least 2.0 Angstroms
and R-factor no greater than 20%, a good quality model would be expected
to have over 90% in the most favoured regions.

saves_01.ps

b)

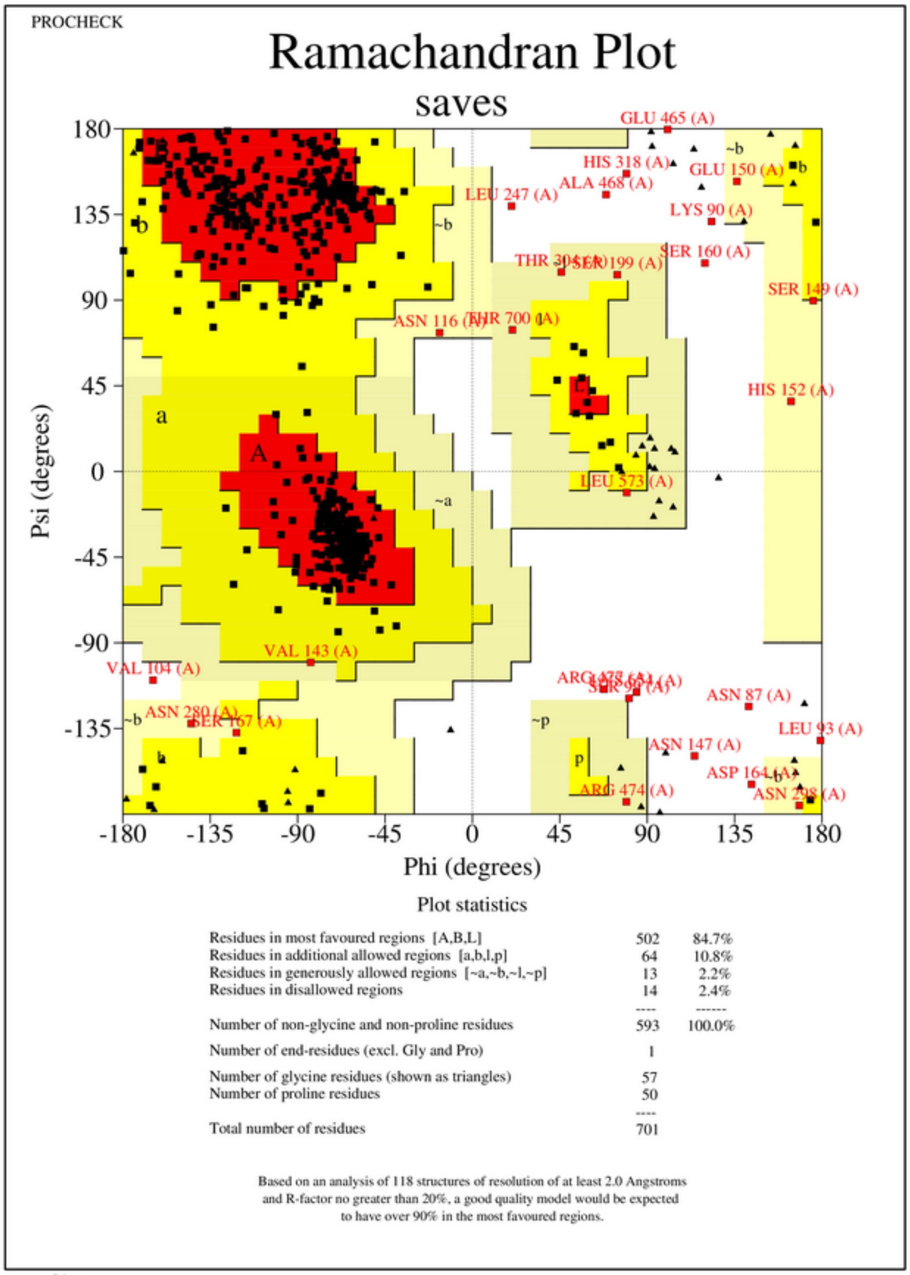
PROCHECK
Ramachandran Plot
saves
Psi (degrees)
Phi (degrees)
GLU 465 (A)
HIS 318 (A)
ALA 468 (A)
GLU 150 (A)
LEU 247 (A)
LYS 90 (A)
SER 160 (A)
SER 149 (A)
ASN 116 (A)
HIS 152 (A)
LEU 573 (A)
VAL 143 (A)
VAL 104 (A)
ASN 87 (A)
LEU 93 (A)
ASN 147 (A)
ASP 164 (A)
ARG 474 (A)
Plot statistics
Residues in most favoured regions [A,B,L] 502 84.7%
Residues in additional allowed regions [a,b,l,p] 64 10.8%
Residues in generously allowed regions [~a,~b,~l,~p] 13 2.2%
Residues in disallowed regions 14 2.4%
Number of non-glycine and non-proline residues 593 100.0%
Number of end-residues (excl. Gly and Pro) 1
Number of glycine residues (shown as triangles) 57
Number of proline residues 50
Total number of residues 701
Based on an analysis of 118 structures of resolution of at least 2.0 Angstroms
and R-factor no greater than 20%, a good quality model would be expected
to have over 90% in the most favoured regions.

c)

saves_01.ps

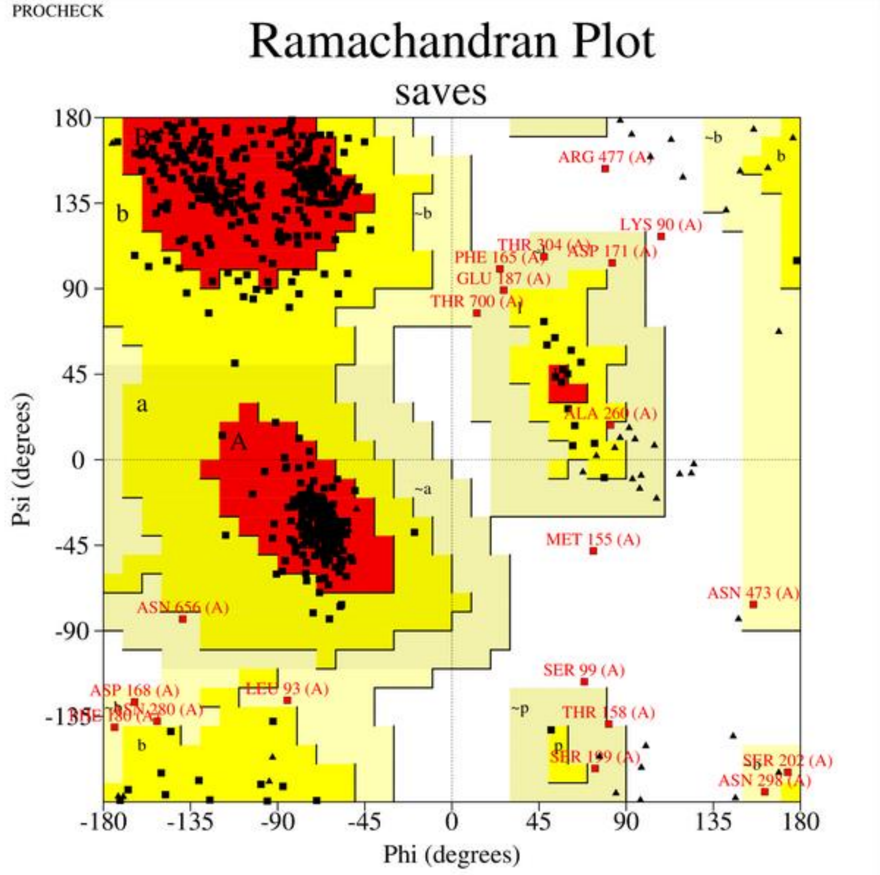

Residues in most favoured regions [A,B,L]	514	86.7%
Residues in additional allowed regions [a,b,l,p]	59	9.9%
Residues in generously allowed regions [~a,~b,~l,~p]	15	2.5%
Residues in disallowed regions	5	0.8%
	----	------
Number of non-glycine and non-proline residues	593	100.0%
Number of end-residues (excl. Gly and Pro)	1	
Number of glycine residues (shown as triangles)	57	
Number of proline residues	50	

Total number of residues	701	

Based on an analysis of 118 structures of resolution of at least 2.0 Angstroms and R-factor no greater than 20%, a good quality model would be expected to have over 90% in the most favoured regions.

d) saves_01.ps

e)

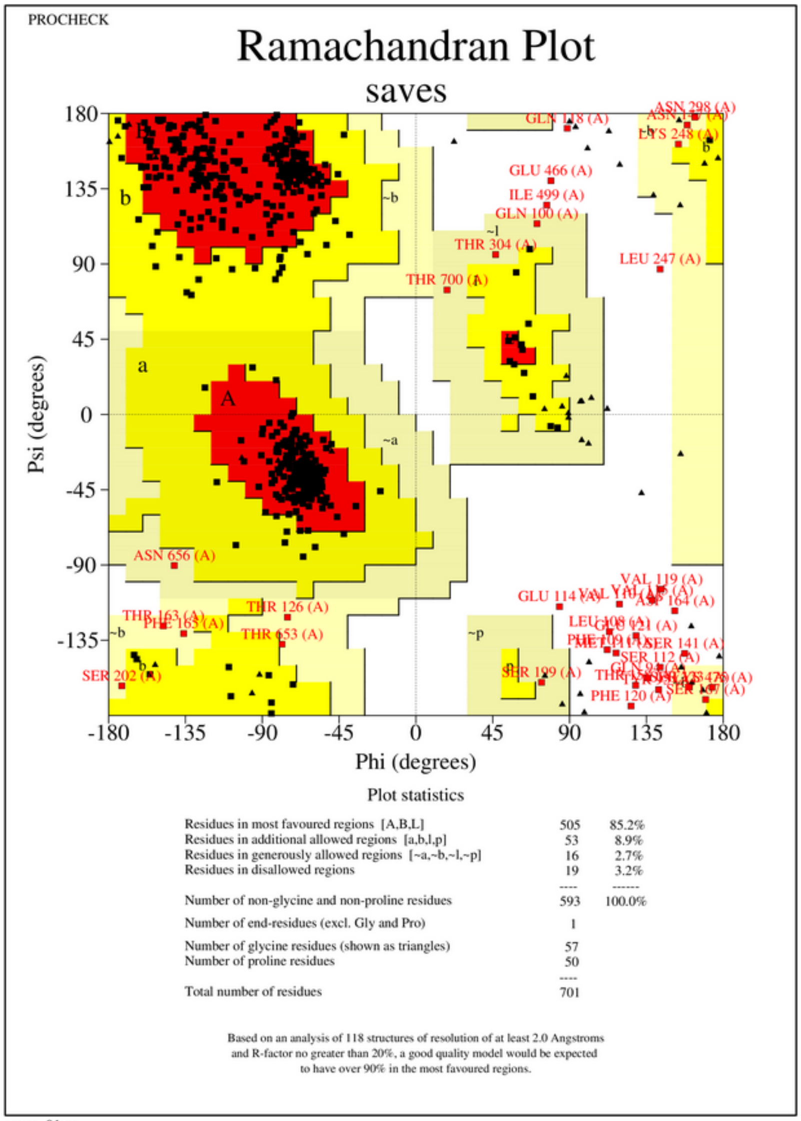
PROCHECK
Ramachandran Plot
saves
Psi (degrees)
Phi (degrees)
Plot statistics
Residues in most favoured regions [A,B,L] 505 85.2%
Residues in additional allowed regions [a,b,l,p] 53 8.9%
Residues in generously allowed regions [~a,~b,~l,~p] 16 2.7%
Residues in disallowed regions 19 3.2%
Number of non-glycine and non-proline residues 593 100.0%
Number of end-residues (excl. Gly and Pro) 1
Number of glycine residues (shown as triangles) 57
Number of proline residues 50
Total number of residues 701
Based on an analysis of 118 structures of resolution of at least 2.0 Angstroms and R-factor no greater than 20%, a good quality model would be expected to have over 90% in the most favoured regions.

saves_01.ps

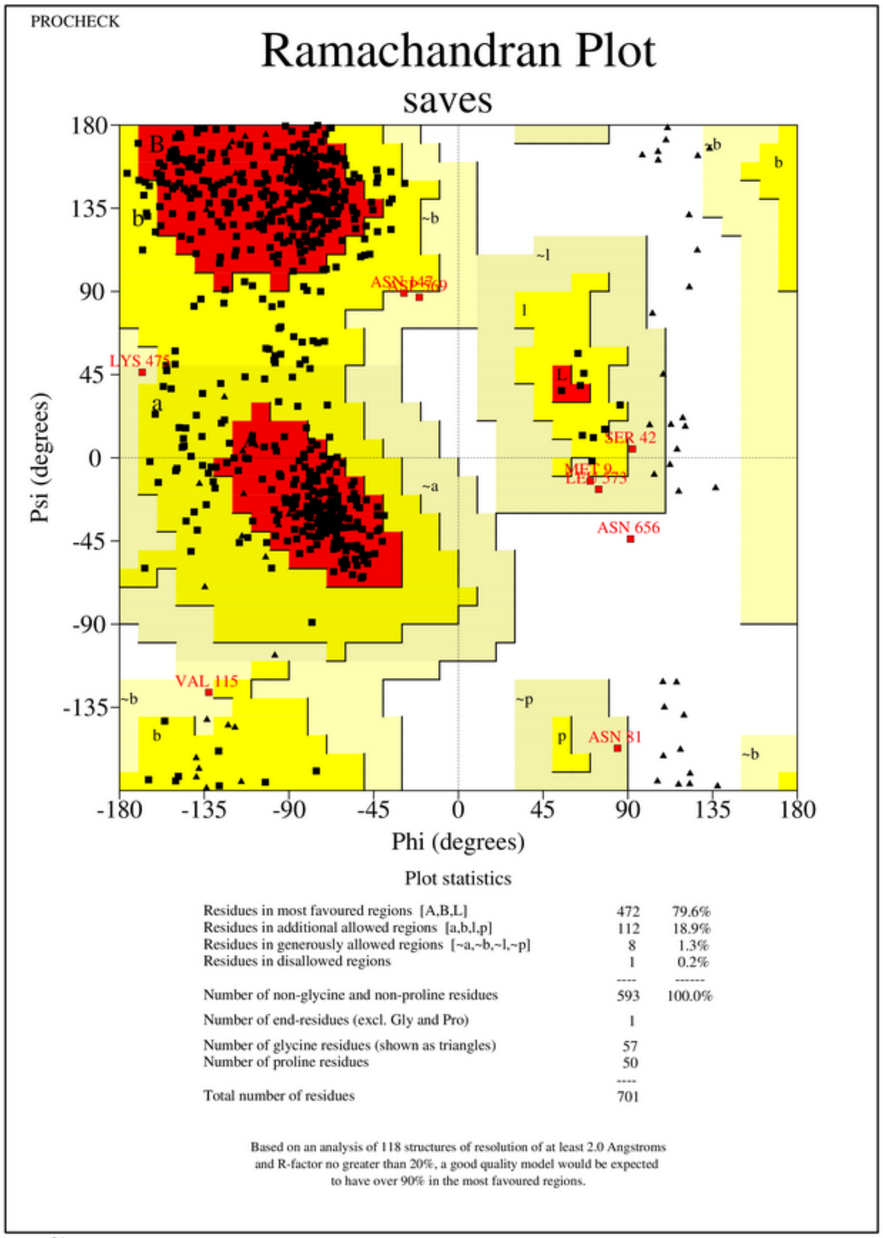
PROCHECK
Ramachandran Plot
saves
Psi (degrees)
Phi (degrees)
180
135
90
45
0
-45
-90
-135
-180
B
b
~b
~l
l
L
a
~a
~p
p
LYS 475
SER 42
MET 9
ASN 656
VAL 115
ASN 81
Plot statistics
Residues in most favoured regions [A,B,L] 472 79.6%
Residues in additional allowed regions [a,b,l,p] 112 18.9%
Residues in generously allowed regions [~a,~b,~l,~p] 8 1.3%
Residues in disallowed regions 1 0.2%
Number of non-glycine and non-proline residues 593 100.0%
Number of end-residues (excl. Gly and Pro) 1
Number of glycine residues (shown as triangles) 57
Number of proline residues 50
Total number of residues 701
Based on an analysis of 118 structures of resolution of at least 2.0 Angstroms
and R-factor no greater than 20%, a good quality model would be expected
to have over 90% in the most favoured regions.

saves_01.ps

f)

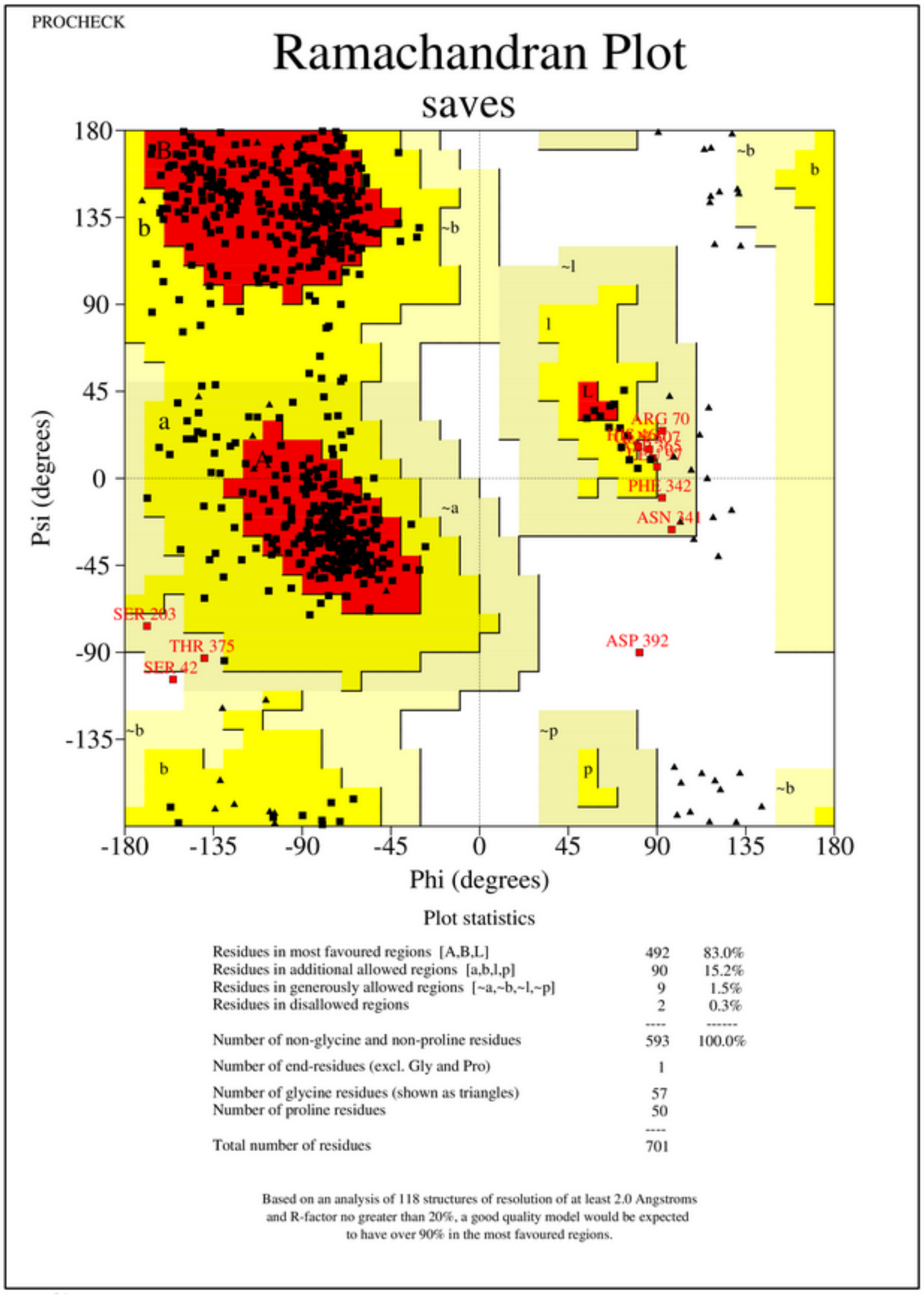
PROCHECK
Ramachandran Plot
saves
Psi (degrees)
Phi (degrees)
180
135
90
45
0
-45
-90
-135
-180
-135
-90
-45
0
45
90
135
180
B
b
~b
a
A
~a
~l
l
L
~p
p
ARG 70
PHE 342
ASN 241
SER 203
THR 375
SER 42
ASP 392
Plot statistics
Residues in most favoured regions [A,B,L] 492 83.0%
Residues in additional allowed regions [a,b,l,p] 90 15.2%
Residues in generously allowed regions [~a,~b,~l,~p] 9 1.5%
Residues in disallowed regions 2 0.3%
---- ------
Number of non-glycine and non-proline residues 593 100.0%
Number of end-residues (excl. Gly and Pro) 1
Number of glycine residues (shown as triangles) 57
Number of proline residues 50

Total number of residues 701
Based on an analysis of 118 structures of resolution of at least 2.0 Angstroms
and R-factor no greater than 20%, a good quality model would be expected
to have over 90% in the most favoured regions.

g) saves_01.ps

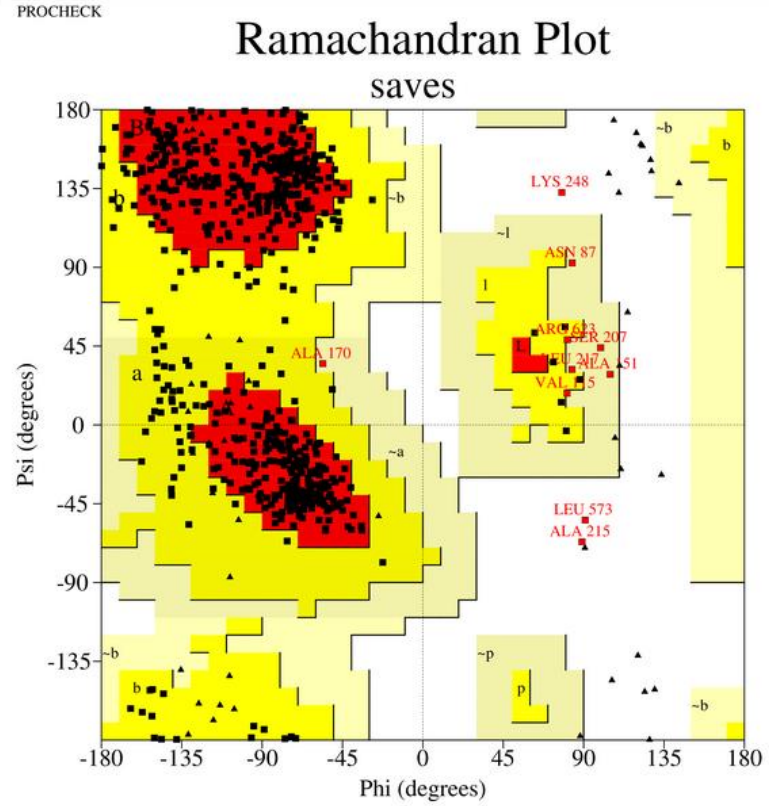

Plot statistics

Residues in most favoured regions [A,B,L]	472	79.6%
Residues in additional allowed regions [a,b,l,p]	111	18.7%
Residues in generously allowed regions [~a,~b,~l,~p]	7	1.2%
Residues in disallowed regions	3	0.5%
	----	------
Number of non-glycine and non-proline residues	593	100.0%
Number of end-residues (excl. Gly and Pro)	1	
Number of glycine residues (shown as triangles)	57	
Number of proline residues	50	

Total number of residues	701	

Based on an analysis of 118 structures of resolution of at least 2.0 Angstroms and R-factor no greater than 20%, a good quality model would be expected to have over 90% in the most favoured regions.

h) saves_01.ps

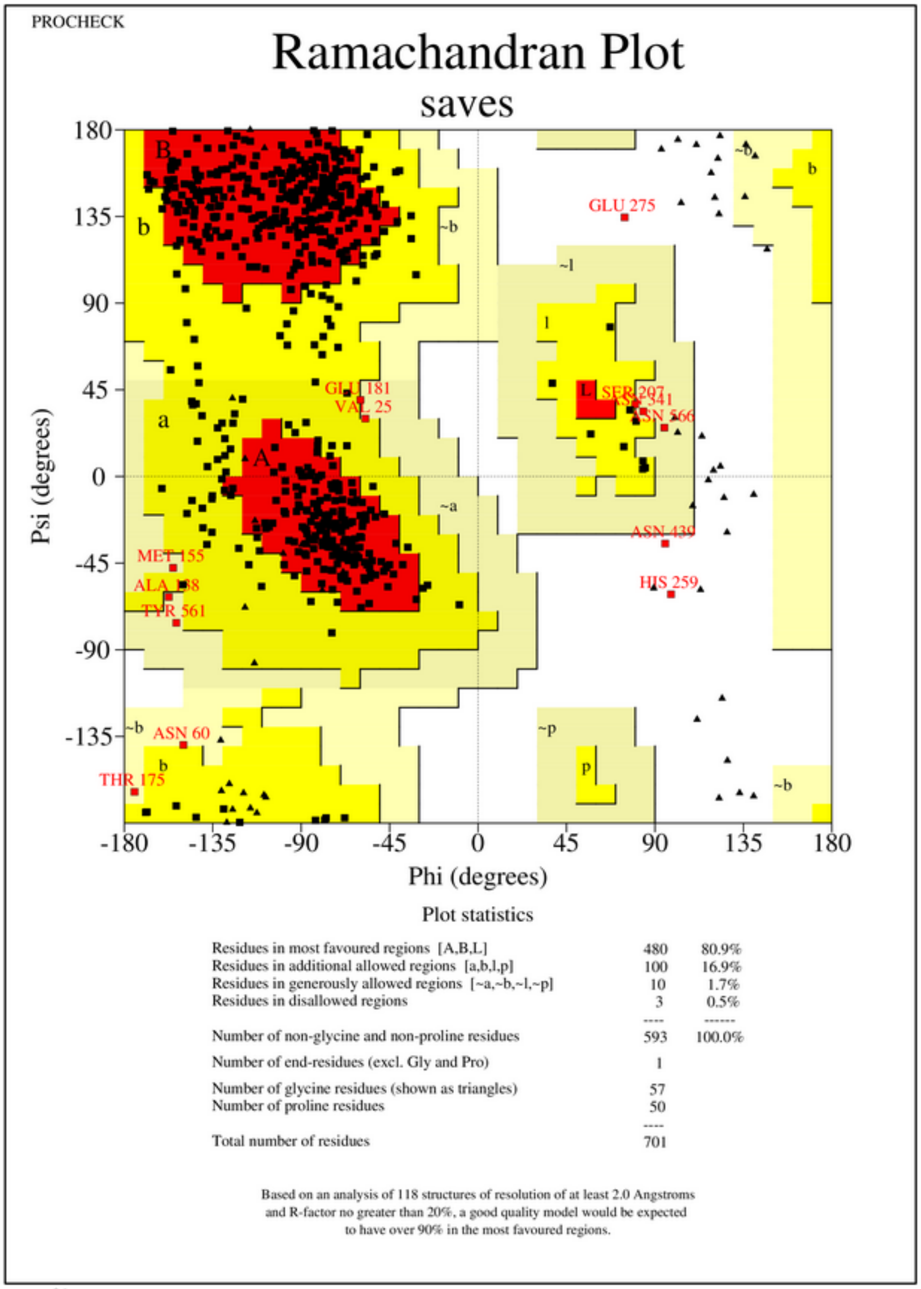

i) saves_01.ps

Supplementary Figure 8. Ramachandran plots generated by PROCHECK: **a-e** models 1-5 from pre-MD simulations, and **f-i** models 1-4 from post-MD simulations. Favored regions (A, B, and L) in red, additional allowed regions (a, b, l, and p) in yellow, generously allowed regions (~a, ~b, ~l, and ~p) in tan, and disallowed regions in white. Squares represent non-glycine residues, and triangles represent non-end glycine residues.

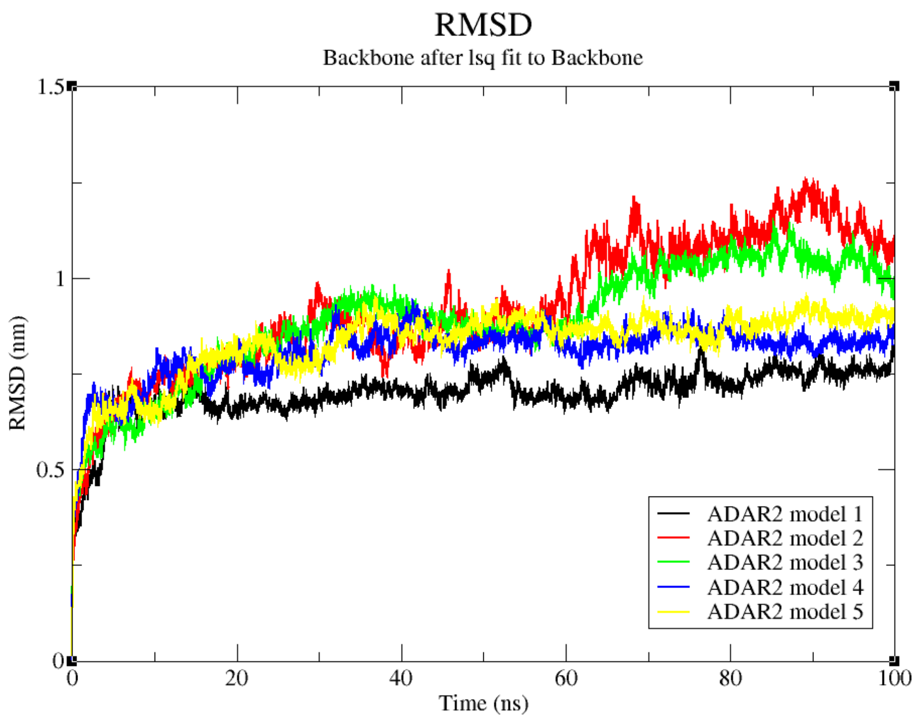
RMSD
Backbone after lsq fit to Backbone
RMSD (nm)
Time (ns)
ADAR2 model 1
ADAR2 model 2
ADAR2 model 3
ADAR2 model 4
ADAR2 model 5

a)

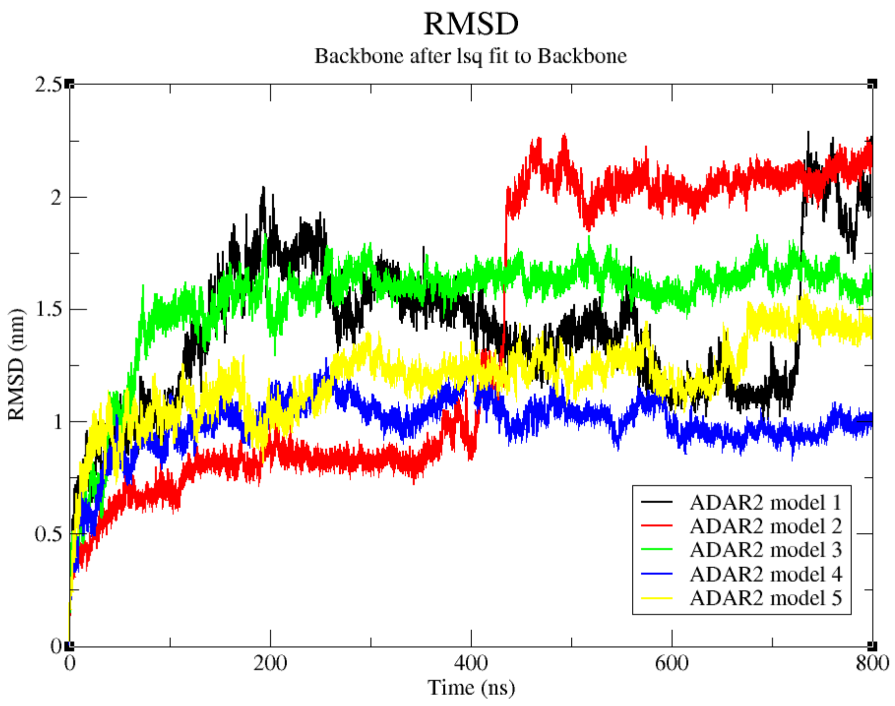
RMSD
Backbone after lsq fit to Backbone
RMSD (nm)
Time (ns)
ADAR2 model 1
ADAR2 model 2
ADAR2 model 3
ADAR2 model 4
ADAR2 model 5

b)

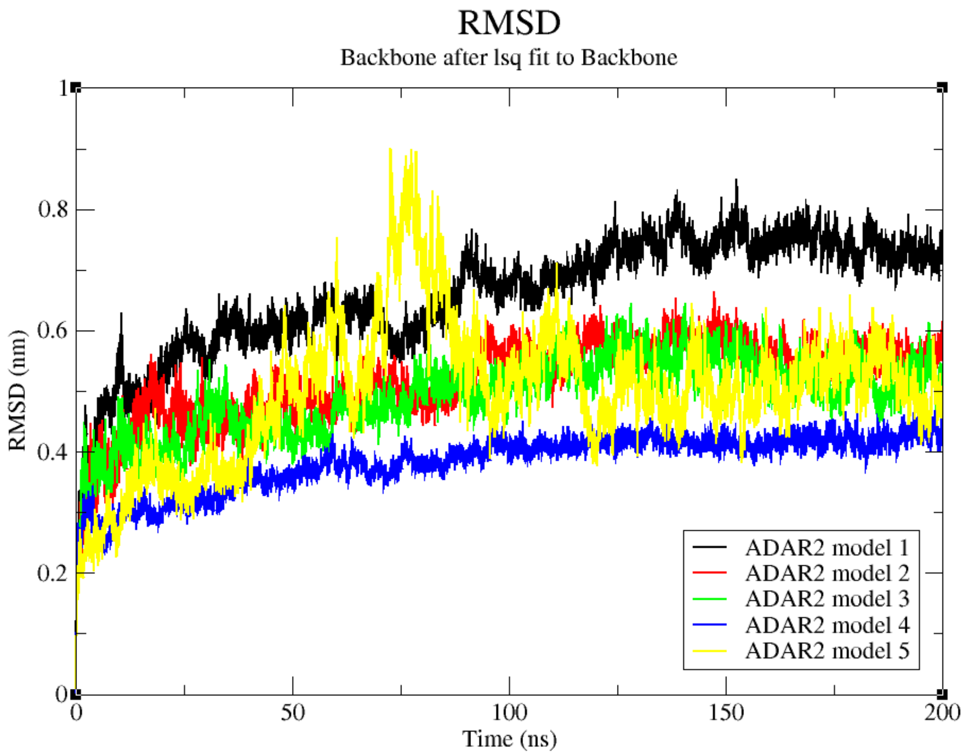

c)

Supplementary Figure 9. RMSD graphs of ADAR2 models during 1.1 μs MD simulation **a)** first 100 ns **b)** 800 ns, and **c)** last 200 ns.

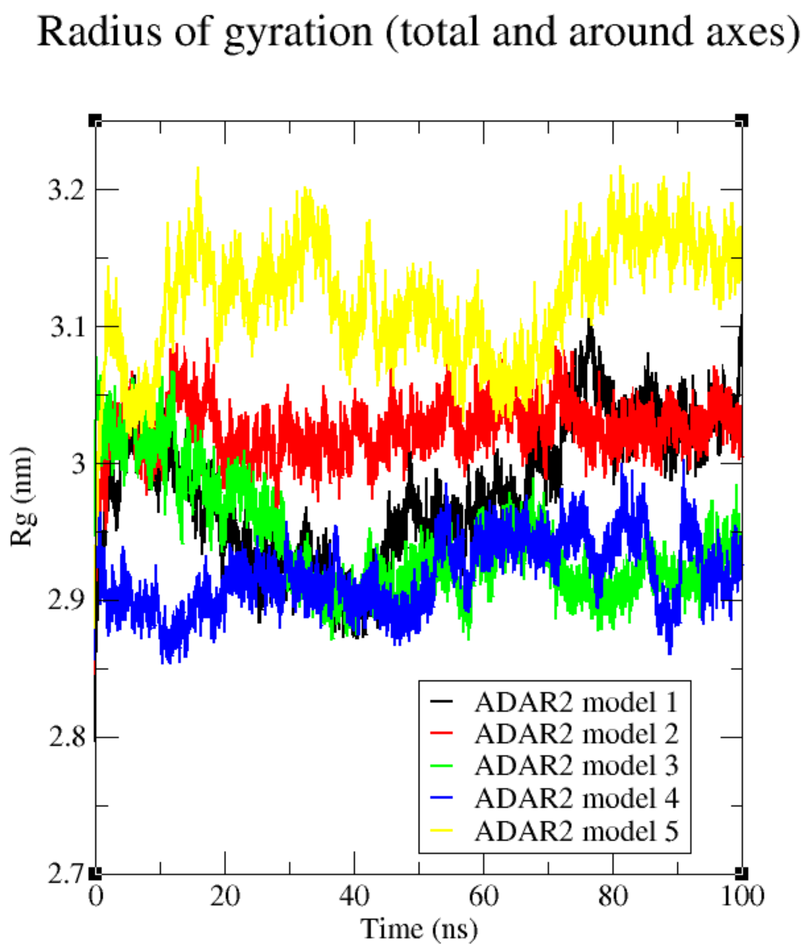
Radius of gyration (total and around axes)
Rg (nm)
Time (ns)
3.2
3.1
3
2.9
2.8
2.7
0
20
40
60
80
100
ADAR2 model 1
ADAR2 model 2
ADAR2 model 3
ADAR2 model 4
ADAR2 model 5

a)

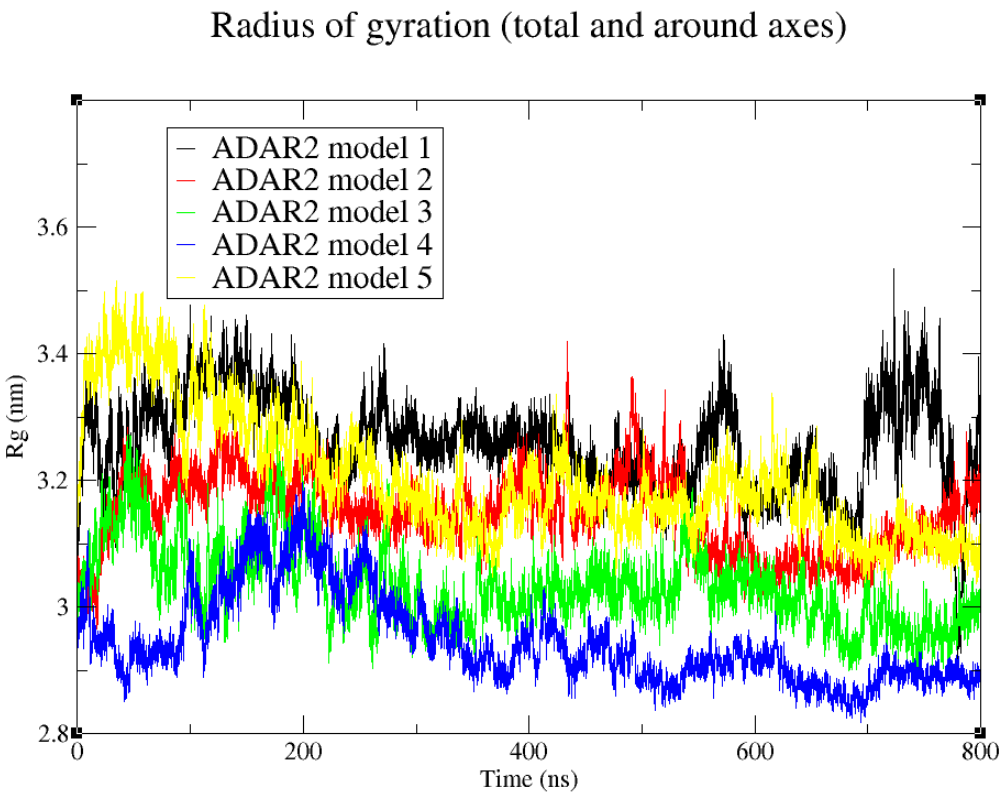
Radius of gyration (total and around axes)
Rg (nm)
Time (ns)
3.6
3.4
3.2
3
2.8
0
200
400
600
800
ADAR2 model 1
ADAR2 model 2
ADAR2 model 3
ADAR2 model 4
ADAR2 model 5

b)

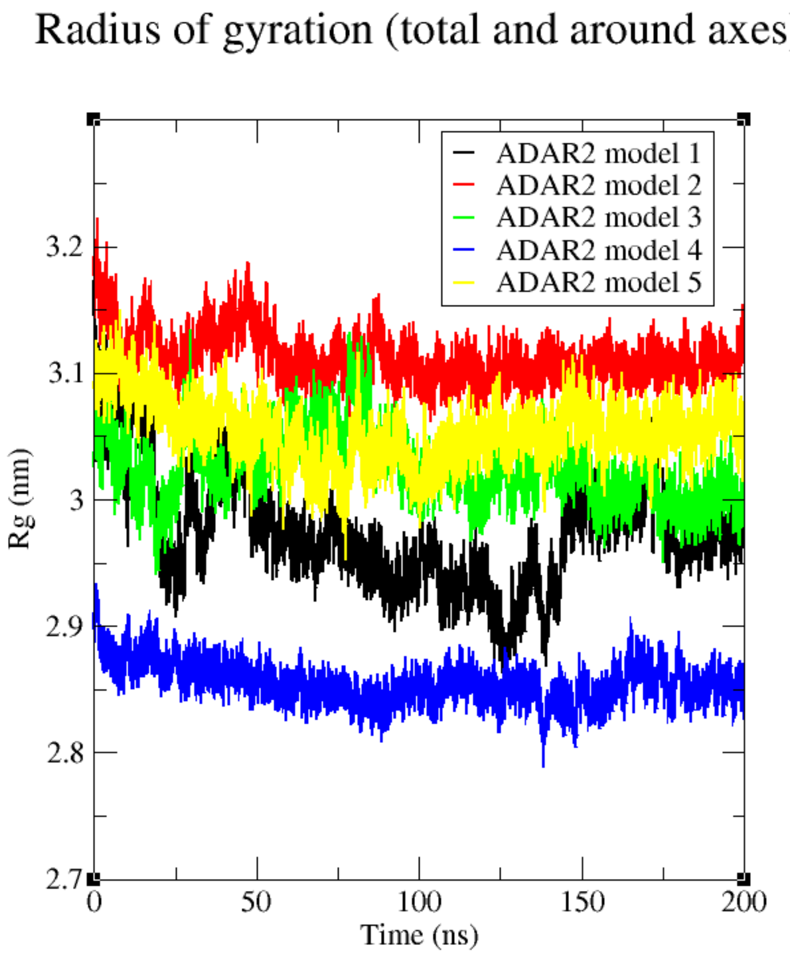

c)

Supplementary Figure 10. Radius of gyration graphs of ADAR2 models during 1.1 μs MD simulation **a)** first 100 ns **b)** 800 ns, and **c)** last 200 ns.

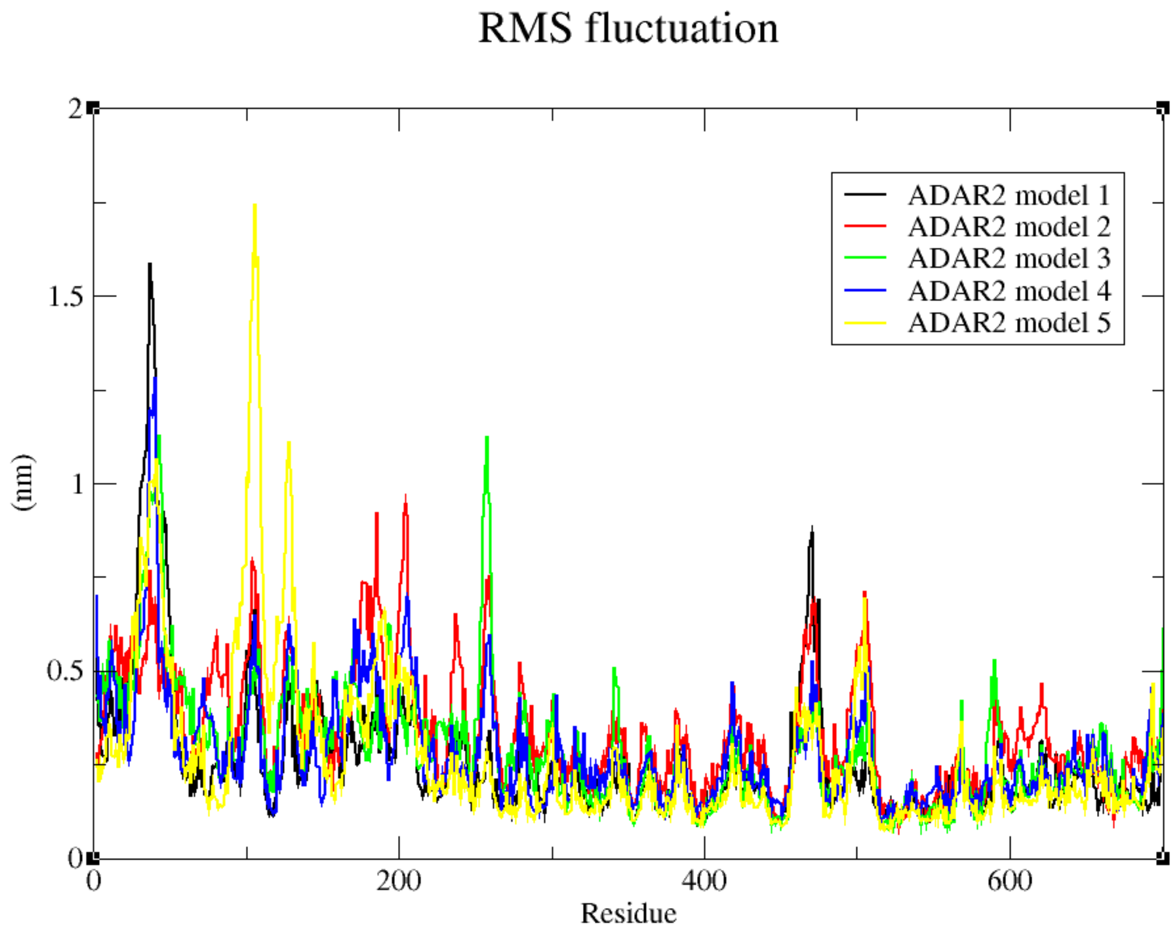
RMS fluctuation
(nm)
Residue
ADAR2 model 1
ADAR2 model 2
ADAR2 model 3
ADAR2 model 4
ADAR2 model 5
0
0.5
1
1.5
2
0
200
400
600

a)

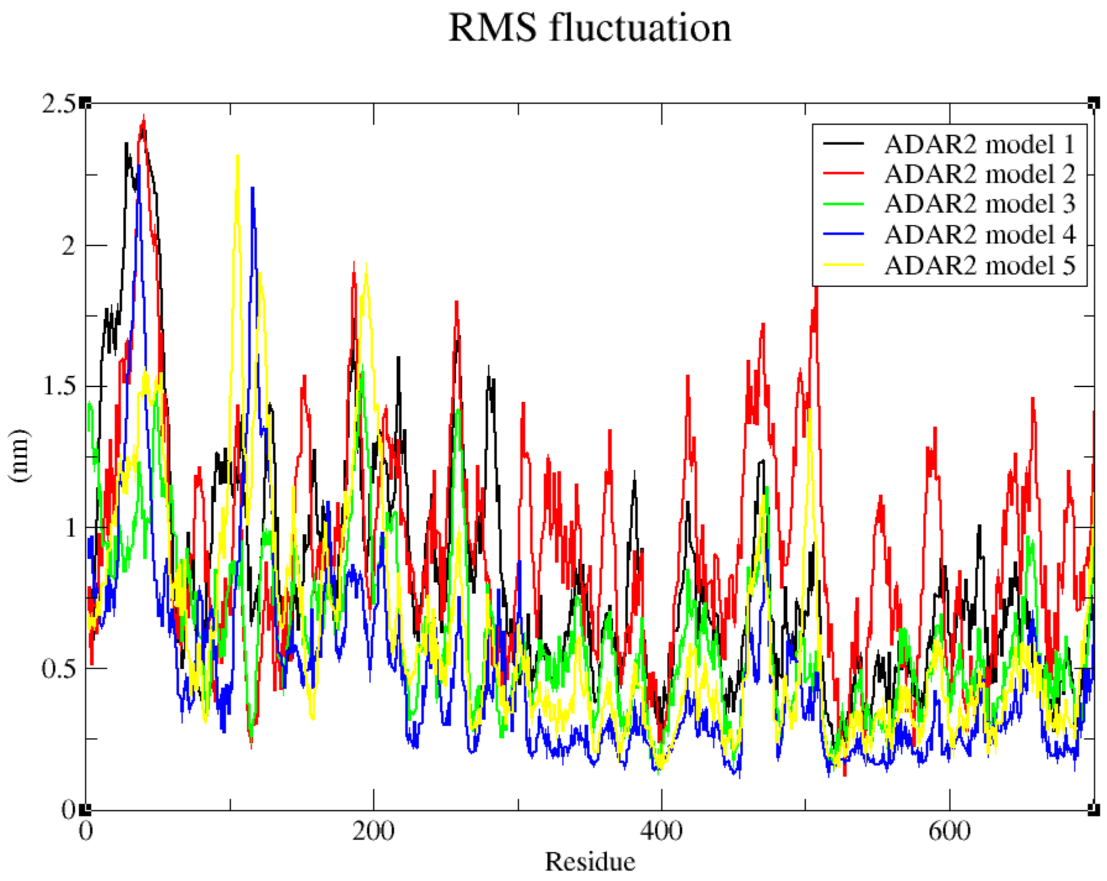
RMS fluctuation
(nm)
Residue
ADAR2 model 1
ADAR2 model 2
ADAR2 model 3
ADAR2 model 4
ADAR2 model 5
0
0.5
1
1.5
2
2.5
0
200
400
600

b)

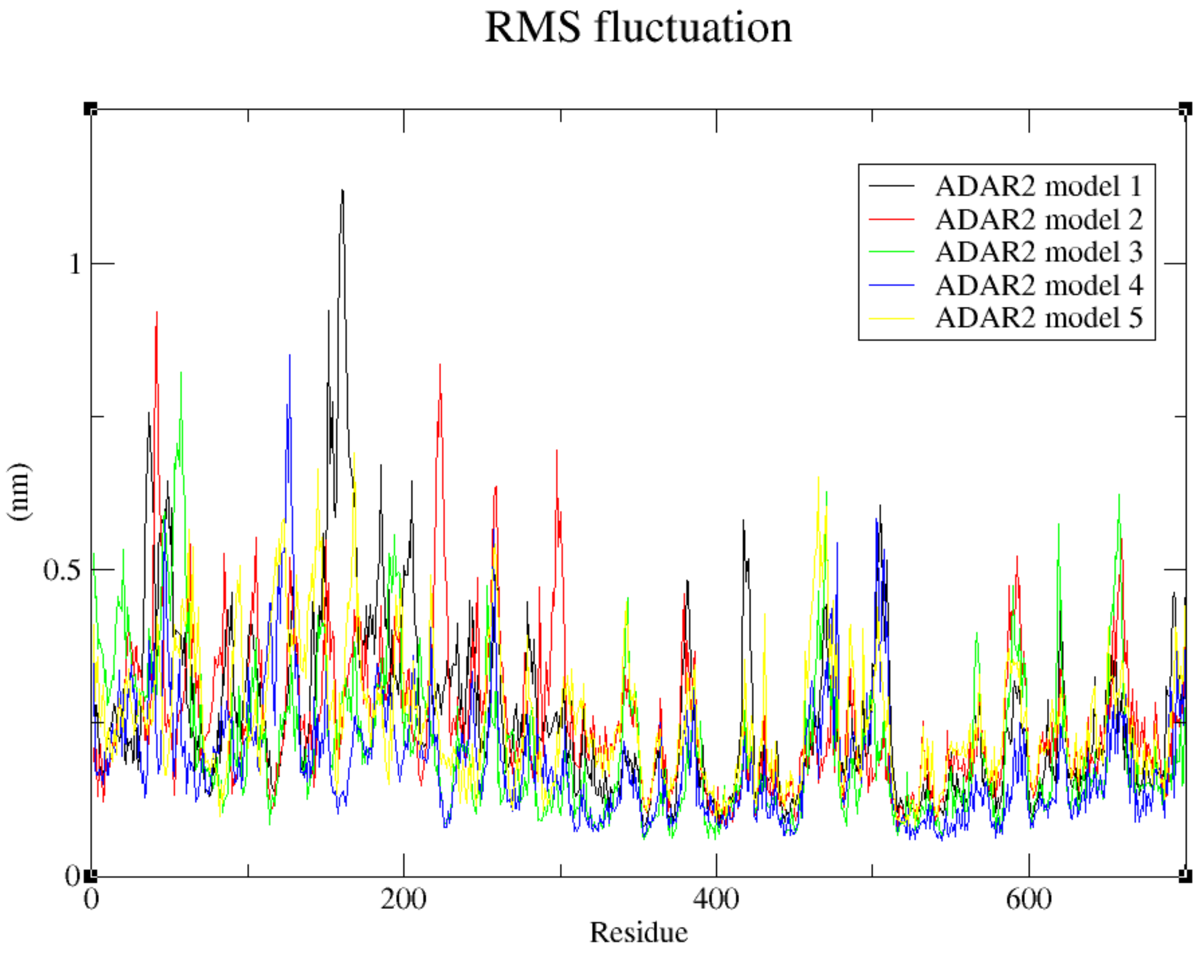

c)

Supplementary Figure 11. RMSF graphs of ADAR2 models during 1.1 μs MD simulation **a)** first 100 ns **b)** 800 ns, and **c)** last 200 ns.

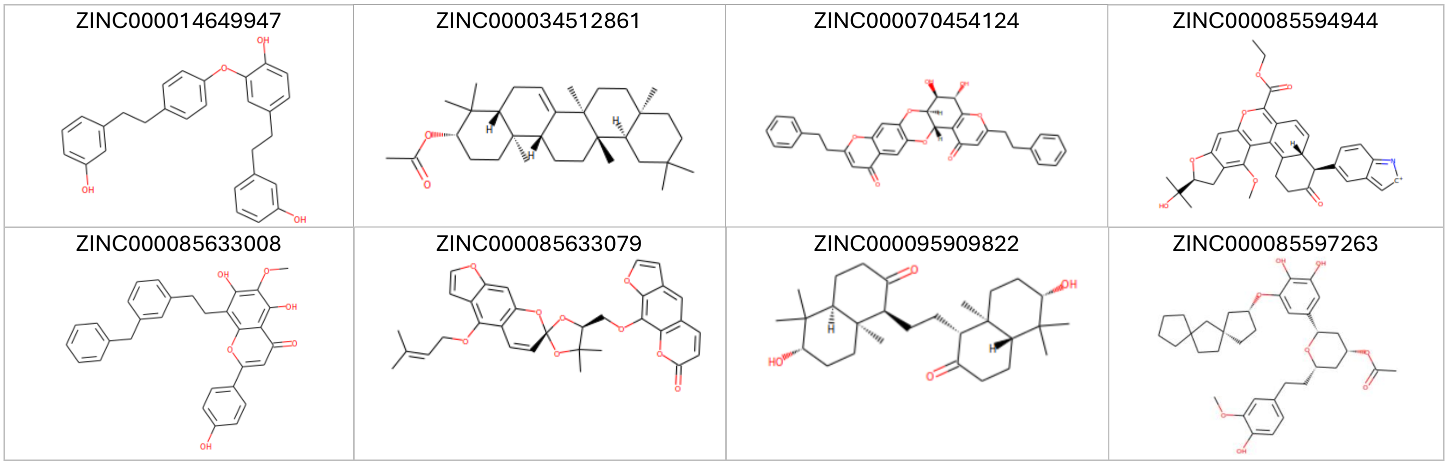

Supplementary Figure 12. Scaffolds of compounds predicted to bind the dsRBD2.
